# Potential for homoacetogenesis via the Wood–Ljungdahl pathway in Korarchaeia lineages from marine hydrothermal vents

**DOI:** 10.1111/1758-2229.13168

**Published:** 2023-05-22

**Authors:** Francesca Vulcano, Petra Hribovšek, Emily Olesin Denny, Ida H. Steen, Runar Stokke

**Affiliations:** ^1^ Department of Biological Sciences, Centre for Deep Sea Research University of Bergen Bergen Norway; ^2^ Department of Earth Science, Centre for Deep Sea Research University of Bergen Bergen Norway; ^3^ Department of Informatics, Computational Biological Unit University of Bergen Bergen Norway

## Abstract

The Wood–Ljungdahl pathway (WLP) is a key metabolic component of acetogenic bacteria where it acts as an electron sink. In Archaea, despite traditionally being linked to methanogenesis, the pathway has been found in several Thermoproteota and Asgardarchaeota lineages. In Bathyarchaeia and Lokiarchaeia, its presence has been linked to a homoacetogenic metabolism. Genomic evidence from marine hydrothermal genomes suggests that lineages of Korarchaeia could also encode the WLP. In this study, we reconstructed 50 Korarchaeia genomes from marine hydrothermal vents along the Arctic Mid‐Ocean Ridge, substantially expanding the Korarchaeia class with several taxonomically novel genomes. We identified a complete WLP in several deep‐branching lineages, showing that the presence of the WLP is conserved at the root of the Korarchaeia. No methyl‐CoM reductases were encoded by genomes with the WLP, indicating that the WLP is not linked to methanogenesis. By assessing the distribution of hydrogenases and membrane complexes for energy conservation, we show that the WLP is likely used as an electron sink in a fermentative homoacetogenic metabolism. Our study confirms previous hypotheses that the WLP has evolved independently from the methanogenic metabolism in Archaea, perhaps due to its propensity to be combined with heterotrophic fermentative metabolisms.

## INTRODUCTION

The Wood–Ljungdahl pathway (WLP) is considered the oldest carbon fixation pathway (Adam et al., 2018; Martin & Russell, 2007). It consists of a methyl branch and a carbonyl branch. In the methyl branch, one molecule of CO_2_ is reduced to one methyl group in a multistep process catalysed by a conserved set of enzymes. In the carbonyl branch, CO_2_ is reduced to CO. The methyl group, the CO, and a CoA are condensed into an acetyl‐CoA molecule by the key enzyme carbon monoxide dehydrogenase (CODH) (Ljungdhal, 1986; Wood 1991; Ragsdale & Pierce, 2008). The WLP is not only used for CO_2_ fixation. In acetogenic bacteria it is combined with phosphotransacetylase (Pta) and acetate kinase (Ack) for substrate‐level phosphorylation via acetate production. In addition, the WLP acts as an electron sink and oxidizes the soluble cofactors NADH and reduced ferredoxin (Fd_red_) derived from the catabolism of a wide spectrum of organic molecules (Ragsdale & Pierce, 2008; Schuchmann & Müller, 2016). In Archaea, the WLP has long been considered restricted to methanogenic Euryarchaeota. In methanogens, it is linked to methane generation via the tetrahydromethanopterin S‐methyltransferase (MTR) and methyl‐coenzyme M reductase (MCR) complexes (Thauer et al., 2008). The recent discovery of several novel archaeal lineages revealed that the enzymes of the WLP and the MCR complex are encoded by several archaeal lineages outside the Euryarchaeota phylum (Berghuis et al., 2019; Borrel et al., 2019; Evans et al., 2015; Vanwonterghem et al., 2016). This expansion within Archaea also pointed to a fragile association between the WLP and methanogenesis (Borrel et al., 2016). In the phylum Asgardarchaeota, an MCR‐independent WLP was found in Lokiarchaeia (Sousa et al., 2016), Thorarchaeia (Seitz et al., 2019), and Odinarchaeia (Macleod et al., 2019; Spang et al., 2019). In the phylum Thermoproteota, some Bathyarchaeia encode a complete WLP (He et al., 2016), while others encode the MCR complex (Evans et al., 2015). Lokiarchaeia and Bathyarchaeia genomes encoding the WLP also encoded an acetyl‐CoA synthetase (ADP‐forming) (Acd) (Schäfer et al., 1993) or the bacterial Pta and Ack. The presence of these enzymes, supported by transcriptomic evidence, suggested that the WLP in these lineages is used for energy conservation and restoration of reduced cofactors via acetate synthesis (He et al., 2016; Orsi et al., 2020). Like Bathyarchaeia and Lokiarchaeia, Korarchaeia have a heterotrophic fermentative metabolism that relies on ferredoxin (Fd) for oxidation of organic matter (Elkins et al., 2008; McKay et al., 2019). Since the WLP—in combination with Acd—mediates substrate‐level phosphorylation, granting a higher energetic yield to fermenters, it seems plausible that some Korarchaeia lineages might encode the WLP module.

In 2019, a complete MCR complex and a dissimilatory sulfate reductase (Dsr) complex were identified in genomes of the Korarchaeia species *Candidatus* (Ca.) Methanodesulfokores washburniensis, however, no associated WLP was detected (Borrel et al., 2019; McKay et al., 2019). Nevertheless, these studies showed that most known Korarchaeia encode a conserved Acd, indicating potential for acetogenesis (McKay et al., 2019). Interestingly, when Korarchaeia genomes from marine hydrothermal vents were analysed for the first time, the presence of the CODH subunits was reported in several of them, suggesting that marine Korarchaeia might encode the carbonyl branch of the WLP (Dombrowski et al., 2018). In this study, we collected 50 novel and diverse Korarchaeia genomes from marine hydrothermal vents and we looked for the presence of a complete WLP. We show that the most deeply branching Korarchaeia genomes with a marine origin encode a complete WLP, and we assess whether its presence could determine a homoacetogenic metabolism.

## RESULTS AND DISCUSSION

### Taxonomic diversity expanded at the root of the Korarchaeia class

**FIGURE 1:**
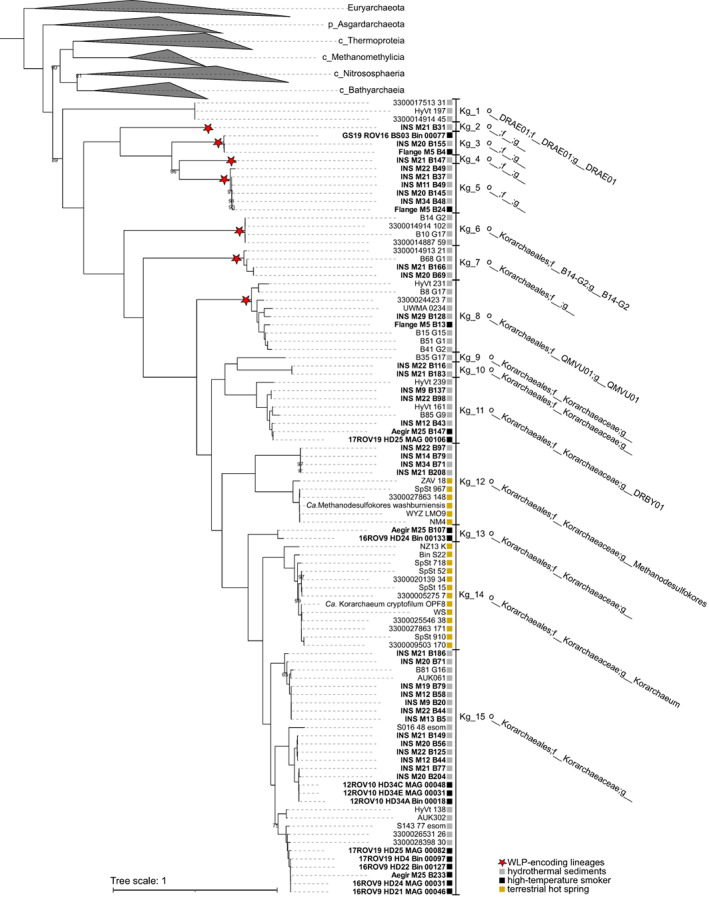
Phylogeny of 42‐concatenated marker genes from 97 Korarchaeia genomes and references. The tree is rooted at midpoint. Where branch support values based on ultrafast bootstrapping are not specified, the confidence for the branch is 100%. Genus‐level lineages defined in this study are indicated to the right (Kg_1 to Kg_15). When available, the GTDB‐tk nomenclature is reported.

So far, mostly Korarchaeia from terrestrial hot springs have been described in detail (Borrel et al., 2019; Elkins et al., 2008; McKay et al., 2019). To expand Korarchaeia taxonomy with genomes from marine hydrothermal environments, DNA was collected and sequenced from chimneys and sediments of several hydrothermal vents along the Arctic Mid‐Ocean Ridge (AMOR) (Table S1). Fifty medium‐quality MAGs (average 80% completeness and 2.5% contamination), were classified by Genome Taxonomy Database‐toolkit (GTDB‐Tk) (r202) as of the class Korarchaeia, phylum Thermoproteota (Table S1B). A phylogenomic analysis using 42 marker genes present in AMOR MAGs and 47 publicly available Korarchaeia MAGs (Table S1B) placed all Korarchaeia MAGs in a basal position within the phylum Thermoproteota (Figure 1 and Table S2). The phylogenetic position of Korarchaeia was confirmed by phylogeny with 115 marker genes (Figure S1 and Table S2). Even though such placement of Korarchaeia is supported by 89% confidence, it agrees with previous phylogenies (Dombrowski et al., 2018; Liu et al., 2021; Spang et al., 2015; Wang et al., 2021; Williams et al., 2017). Using a combination of phylogenomic analysis, Average Amino acid Identity (AAI) and GTDB‐tk classification, 15 genus‐level lineages were identified (Kg_1 to Kg_15) within Korarchaeia, of which only six (Kg_1, 6, 8, 11, 12, 14) were already defined in the GTDB (Figure 1). Phylogenomic analysis with 115 marker genes clustered the genus‐level lineage Kg_1 with Bathyarchaeia, indicating that the phylogenetic placement of this lineage is uncertain (Figure S1). Notably, the GTDB‐tk assigned four AMOR MAGs to genus Methanodesulfokores (Kg_12), that previously comprised only genomes with a terrestrial origin (Table S1B). However, based on the commonly accepted threshold for genus separation of >65% and < 95% AAI (Goris et al., 2007; Konstantinidis et al., 2017), the four AMOR genomes should represent a separate genus‐level marine lineage (Figure S2). We preferred the GTDB‐tk classification for consistency with the GTDB. The GTDB‐tk assigned all other MAGs with terrestrial origin to genus Korarchaeum (Kg_14). Nevertheless, its members shared 65%–66% AAI identity to MAGs within Kg_15 (Figure S2), suggesting that the boundaries between the two genera are ambiguous. The phylogenomic analysis placed four of the genus‐level lineages from this study (Kg_2,3,4,5) at the root of the Korarchaeia class with high support (Figure 1). Notably, Kg_2, 3, 4 and 5 could not be classified further than class level and likely represent one or multiple novel deep‐branching orders. One more genus‐level lineage from this study, Kg_7, unclassified at family level, occupied a rather basal position in the order Korarchaeales. Of the 15 genus‐level lineages recognized, 13 comprised genomes recovered uniquely from hydrothermal sediments, with representatives from both hydrothermal sediment and high‐temperature black smokers. Only two genera comprised genomes from terrestrial hot springs. These were genus Ca. Korarchaeum and genus Ca.*s* Methanodesulfokores, that respectively include the well‐characterized Ca. Korarchaeum cryptophilum (OPF8) (Elkins et al., 2008) and the MCR‐containing Ca. Methanodesulfokores washburniensis (Borrel et al., 2019; McKay et al., 2019). The phylogenetic placement of Ca. Korarchaeum and Ca. Methanodesulfokores suggests that they likely have evolved from a marine ancestor after two independent events of invasion of terrestrial hot springs (Figure 1).

Overall, the phylogenetic analysis of genomes of marine hydrothermal origin substantially expanded the Korarchaeia class, as well as all previously known genera, revealing an undiscovered taxonomic richness, especially at the root of the class.

### Potential for homoacetogenesis in deep branching Korarchaeia

**FIGURE 2:**
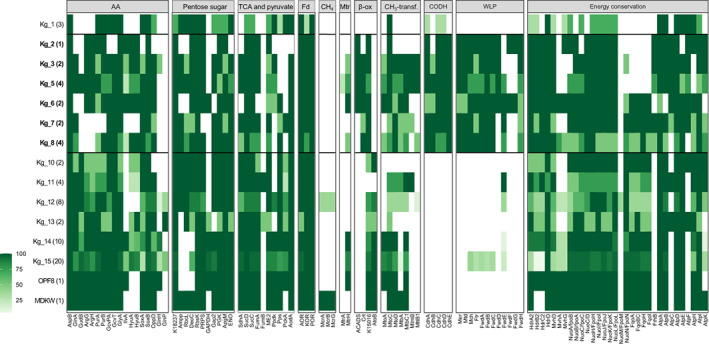
Distribution of key metabolic genes in Korarchaeia genera. The number of genomes in each genus is indicated in parenthesis. The WLP‐encoding lineages are surrounded by a black line. The list of the accession numbers used for the presence/absence matrix is given in Table S3.

Having revealed a high number of taxonomically unclassified and deep‐branching Korarchaeia, we reconstructed the metabolisms of all genera to identify metabolic novelties and screen for the presence of the WLP. By genome annotation, we confirmed that all genera shared potential for fermentative growth on amino acids. Aspartate, glycine and glutamate can be converted to pyruvate and acetyl‐CoA via the glycine dehydrogenase GcvPA and GcvT, the glycine hydroxymethyltransferase GlyA, the argininosuccinate synthase ArgG and argininosuccinate lyase ArgH, and the aspartate aminotransferase AspB, as previously described (Elkins et al., 2008; McKay et al., 2019) (Figure 2, Figure S3 and Table S3). The conservation of the oligopeptide transport system OppD, the glutamine transport system, and several peptidases further supports Korarchaeia capacity to grow on amino acids (Figure 2, Figure S3 and Table S3). All genera encoded the potential to convert nucleic acid‐derived pentoses into glycolysis intermediates and downstream to pyruvate. Deoxy‐ribose‐5‐phosphate and ribose‐5‐phosphate can be converted to 3‐phospho‐glycerate via the AMP phosphorylase DeoA, the ribose 1,5‐bisphosphate isomerase (K18237) and the ribulose‐bisphosphate carboxylase Rubisco III (Sato et al., 2007), or via the deoxyribose‐phosphate aldolase DeoC to glyceraldehyde 3‐phosphate. The pyruvate:ferrodoxin oxidoreductase POR was utterly conserved (Figure 2), suggesting that pyruvate is actively converted to acetyl‐CoA. Acetyl‐CoA is then converted to acetate by the conserved Acd (EC:6.2.1.13) and ATP is generated in this step via substrate‐level phosphorylation (Figure S3).

**FIGURE 3:**
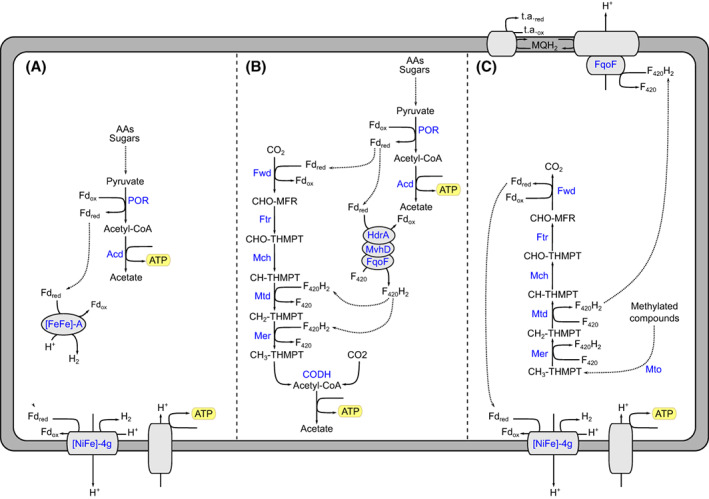
Reconstruction of the main putative metabolisms identified in Korarchaeia. The canonical fermentative metabolism (Elkins et al., 2008; McKay et al., 2019) (A) is compared to the putative WLP‐mediated homoacetogenic metabolism (B) in WLP‐encoding genera and to a putative methoxylated compounds‐dependent metabolism where the WLP is run in the oxidative direction (C).

In addition to the fermentative metabolism, a complete WLP was identified in several deep‐branching Korarchaeia lineages (Kg_2,3,4,5,6,7,8) (Figure 1, Figure 2, and Table S3). The wide distribution of the pathway suggests that Korarchaeia ancestor encoded the WLP and lost it during the evolution. In addition to a complete CODH complex (CdhABCDE), the genes of the methyl‐branch of the WLP were identified (Figure 2 and Table S4). These are the genes of the formyl‐methanofuran dehydrogenase complex (*fwd*), the genes formylmethanofuran: tetrahydromethanopterin formyltransferase (*ftr*), N^5^,N^10^‐methenyltetrahydromethanopterin cyclohydrolase (*mch*), F_420_H_2_‐dependent methylenetetrahydromethanopterin dehydrogenase (*mtd*) and F_420_H_2_‐dependent N^5^,N^10^‐methylenetetrahydromethanopterin reductase (*mer*). As proposed for Lokiarchaeia (Orsi et al., 2020), the WLP can act as an electron sink in a heterotrophic metabolism when used in the reductive direction. Microbes using this metabolism are defined as homoacetogens, as acetate is the only end‐product generated. The conversion of pyruvate to acetyl‐CoA produces Fd_red_. In fermentation, Fd_red_ can be recycled via cytoplasmic H_2_‐evolving hydrogenases. When the WLP is present, the complex Fwd can channel the reducing power of Fd_red_ for the reduction of CO_2_ to formylmethanofuran, in the first reaction of the pathway. Downstream, Mch and Mtd are used to further reduce CO_2_. These enzymes use the reduced form of F_420_ (F_420_H_2_) as cofactor. Since no F_420_H_2_ is produced during fermentation, F_420_ (E^0'^ = −340 mV, Ney et al., 2017) can be reduced instead by Fd_red_ (E^0'^ = −420 mV). This step can be performed via a soluble F_420_: oxidoreductase, FqoF, as proposed for *Archaeoglobus fulgidus* (Hocking et al., 2014). FqoF has high homology to other F_420_‐dependent enzymes such as the formate dehydrogenase *Methanobacterium formicicum* (FdhB) or FpoF of *Methanocaldococcus jannaschii* (Bäumer et al., 2000; Brüggemann et al., 2000). The gene coding for the F_420_ hydrogenase subunit beta *frhB* (K00125) was encoded in all Korarchaeia lineages carrying the WLP (Figure 2). The gene *frhB* often co‐located with the genes heterodisulfide reductase *hdrA* (K03388) and F_420_‐non‐reducing hydrogenase *mvhD* (K14127). These three genes could encode a complex where HdrA performs the oxidation of Fd_red_ and FqoF performs the reduction of the F_420_. The FeS cluster of the MvhD subunit could be used for electron transfer (Figure 3B). The end‐product of the WLP, acetyl‐CoA, can again be converted to acetate for generation of an additional molecule of ATP. Hence, homoacetogenesis grants Korarchaeia one additional ATP molecule compared to fermentation alone.

### Hydrogenases and proton motive force in Korarchaeia

The WLP‐encoding Korarchaeia shared with all other Korarchaeia one cytoplasmic [FeFe]‐hydrogenase group A. This hydrogenase typically couples the reoxidation of Fd_red_ to H_2_ evolution during fermentation. Notably, the hydrogenase database, HydDB (Søndergaard et al., 2016), identified a previously undescribed [NiFe]‐hydrogenase group 4 g, that was common to all Korarchaeia genera (Figure S4). Group 4 g hydrogenases are related to the *Pyrococcus furiosus* Mbh (Yu et al., 2020) (group 4d), a multi‐subunit membrane complex that couples the oxidation of Fd_red_ to H_2_ evolution combined with translocation of protons for generation of proton gradient across the membrane (Sapra et al., 2003). Furthermore, genes co‐locating with the [NiFe]‐hydrogenase group 4 g have high sequence similarity to subunits of the Nuo complex, including the NuoLM‐like transmembrane subunits (Figure S5) (Friedrich & Weiss, 1997; Schut et al., 2013), suggesting a role in proton translocation. The presence of these genes suggests that Korarchaeia could exploit the reducing power of Fd_red_ to retrieve additional ATP via proton motive force. A rather complete V‐type ATP synthase complex was identified in all Korarchaeia genera (Figure 2). Since this mechanism can grant Korarchaeia a greater energy yield than what is achieved by simple substrate‐level phosphorylation, the role of the [FeFe]‐hydrogenase is unclear. However, its conservation in all lineages argues in favour of its active role in Korarchaeia. The coexistence of these two hydrogenases might perhaps be linked to growth conditions and nutrients availability. Multi‐subunit membrane complexes such as the [NiFe]‐hydrogenase and the ATP‐synthase are probably synthetized at high energetic costs. This method of energy conservation might be an advantageous strategy only when nutrients are available, and high concentrations of Fd_red_ are produced by catabolism. The type of substrate utilized might also determine the amount of Fd_red_ produced per metabolic cycle. With reduced nutrients availability, the synthesis of a simple cytosolic hydrogenase might be less energy‐demanding and still sufficient to maintain an active metabolism.

In addition to the [FeFe]‐hydrogenase and the [NiFe]‐hydrogenase 4 g, the lineage Kg_8 encoded one [NiFe]‐hydrogenase group 1 h and one [NiFe]‐hydrogenase group 3c (Figure S4). Considering the presence of these hydrogenases, we evaluated the possibility that Korarchaeia of lineage Kg_8 could be chemolithotrophs. The [NiFe]‐hydrogenase group 1 h is described as a hydrogen‐uptaking hydrogenase putatively membrane‐associated prevalently found in Actinobacteria (Søndergaard et al., 2016). Its sequence showed high similarity with the domain TIGR03295 of the coenzyme_F_420__hydrogenase, suggesting a possible role of the [NiFe]‐hydrogenase 1 h in the reduction of the F_420_. The [NiFe]‐hydrogenase group 3c could instead donate electrons for the reduction of Fd to the electron bifurcating heterodisulfide reductase HdrABC. The coexistence of these two hydrogenases in combination with the [NiFe]‐hydrogenase group 4 g might allow Korarchaeia of lineage Kg_8 to grow on hydrogen and CO_2_ as acetogens, rather than peptide fermenters. It is worth mentioning that the WLP‐encoding Korarchaeia did not encode the key enzyme ATP citrate lyase, necessary for channelling the WLP‐derived acetyl‐CoA into the TCA cycle via addition to oxaloacetate and for the synthesis of key amino acids precursors such as α‐ketoglutarate. Genera Kg_3,4,5 encoded a citryl‐CoA lyase subunit beta (EC 4.1.3.34/ EC 4.1.3.6), but they lacked the citryl‐CoA synthase. Thus, autotrophic growth in WLP‐encoding Korarchaeia remains questionable.

Overall, the WLP‐encoding Korarchaeia genomes did not encode known pathways for respiration of inorganic compounds that could drive the WLP (Figure S6). Nevertheless, a membrane molybdopterin oxidoreductase PhsA was identified in lineages Kg_2 and Kg_3. This could represent a terminal reductase involved in thiosulfate or nitrate reduction. The potential for sulfite reduction by hydrogen via Dsr remains limited to genus Ca. Methanodesulfokores (McKay et al., 2019).

### Oxidative WL pathway

Recently, Welte and colleagues described a methoxydothrophic metabolism in *A. fulgidus* in which methyl groups from methoxylated compounds are channelled into the WLP by the gene cluster of methyltransferases MtoABCD (Welte et al., 2021). When *A. fulgidus* grows on methoxylated aromatic compounds, it binds the derived methyl groups directly to a tetrahydromethanopterin via Mto enzymes and converts the resulting methyl‐tetrahydromethanopterin to CO_2_. This is achieved in subsequent steps of oxidation by running the WLP in the oxidative direction. Redox balance is maintained by a combination of cytoplasmic and membrane‐bound hydrogenases (Hdr/Vhu – Vht) and the Fqo membrane complex. Welte and colleagues identified sequences related to MtoA, MtoC and MtoD in MAG B41_G2 of the WLP‐encoding genus QMVU01 (Kg_8). Such a finding suggests that WLP‐encoding Korarchaeia might also be able to fully oxidize methylated compounds to CO_2_ by running the WLP in the oxidative direction (Figure 3C). Mto‐like sequences were found in several WLP‐encoding Korarchaeia (Kg_4,5,6,7) (Figure 2). The oxidative WLP produces F_420_H_2_ and Fd_red_. In Korarchaeia, the Fd_red_ could be oxidized by the membrane [NiFe]‐hydrogenase 4 g and used for ATP production. The F_420_H_2_ could instead be oxidized by a membrane bound menaquinone reducing Fqo. Interestingly, the membrane components of the Fqo complex were identified in Kg_6,7,8, confirming the potential for electron transfer from F_420_H_2_ to a menaquinone (MQ), as in *A. fulgidus* (Brüggemann et al., 2000; Hocking et al., 2014). However, none of these genera encoded membrane complexes for reduction of a terminal electron acceptor. Only Kg_3 carried a complete membrane complex consisting of a menaquinone‐interacting subunit and a NarG‐like subunit (Figure S7). The terminal electron acceptor remains unknown. In conclusion, despite the presence of several genes involved in transfer of methyl groups, Korarchaeia do not seem to contain the complete protein apparatus necessary to maintain the redox balance and their ability to grow on methylated compounds requires further investigation.

### Similarities between Korarchaeia, Asgardarchaeia and Thermoproteota

Metabolically, WLP‐encoding deep‐branching Korarchaeia are highly similar to several classes of Asgardarchaeota, which have been recently proposed to have originated from a hydrogen‐dependent acetogen (Liu et al., 2021) and to intake carbon mainly heterotrophically (Spang et al., 2019). Besides Lokiarchaeia, whose capability to perform homoacetogenesis has been demonstrated (Orsi et al., 2020), lineages of Thorarchaeia, Odinarchaeia and several newly reconstructed Asgardarchaeota carry the genetic potential for a similar metabolism. Besides the key fermentative enzymes, they encode a full or partial archaeal WLP and a cytoplasmic module for reduction of F_420_ and Fd (Liu et al., 2021; Macleod et al., 2019; Spang et al., 2019). Since most Asgardarchaeota are heterotrophs and take part in remineralization of organic matter (Baker et al., 2020), the WLP might be linked to fermentation rather than being used for autotrophy in these lineages.

Even though our genomic analysis indicated that only the fermentative and homoacetogenic metabolisms are actively used in Korarchaeia, several components of oxidoreductases complexes, previously found in proposed methanogenic Thermoproteota, were conserved in all Korarchaeia genera. These complexes were found in genomes of Bathyarchaeia and Methanomethylicia (previously Ca. Verstraetearchaeota) where they could take part in methylotrophic methanogenesis (Evans et al., 2015; Vanwonterghem et al., 2016). They include a Fpo‐associated HdrD/GlcD (Heterodisulfide reductase/Glycolate oxidase) (Figure 2) suggested to couple the oxidation of lactate to the reduction of the MCR‐derived heterodisulfide CoM‐S‐S‐CoB, with corresponding proton translocation (Lang et al., 2015; Vanwonterghem et al., 2016). Bathyarchaeia and Methanomethylicia also encode a complete bifurcating Hdr/Mvh heterodisulfide reductase/hydrogenase ([NiFe]‐hydrogenase 3b) complex used for simultaneous reduction of Fd and heterodisulfide by H_2_. All Korarchaeia genera encode a partial HdrABC/MvhD complex instead. Only in a few lineages is the complex associated with the hydrogenase unit MvhA (McKay et al., 2019). Even though the role of the heterodisulfide reductases is unclear due to absence of the MCR in most Korarchaeia lineages, the high degree of conservation of HdrABC/MvhD and HdrD/GlcD units is puzzling. Such conservation further supports the hypothesis of a methylotrophic ancestor for Archaea (Wang et al., 2021), but makes vestigial nature of these genes less likely. These Fd‐dependent cytosolic units might have developed additional roles in the cell, perhaps not directly linked to energy conservation and generation of proton motive force. Alternatively, they could simply use heterodisulfide cofactors other than the CoM‐S‐S‐CoB.

Some WLP‐encoding Korarchaeia encoded the subunit H of the MTR complex (MtrH), proposed to participate in methylamine: coenzyme M methyl transfer activity in Methanomethylicia and Bathyarchaeia (Evans et al., 2015; Vanwonterghem et al., 2016). Furthermore, several methyltransferases were encoded in WLP‐encoding Korarchaeia (Figure 2). Altogether these findings, combined with the discovery of a Methanomethylicia‐like MCR in Ca. Methanodesulfokores washburniensis (Hua et al., 2019; McKay et al., 2019; Wang et al., 2021), indicate that ancient Korarchaeia might have been capable of methylotrophic methanogenesis, as proposed for the related Methanomethylicia. As discussed by Wang et al. (2021), in methylotrophic methanogens the WLP does not take part in methanogenesis, and it is instead used independently for carbon fixation. The Korarchaeia ancestor might have encoded a methylotrophic methanogenic pathway and a WLP for carbon fixation, that underwent parallel evolutionary paths, resulting in the loss of MCR and methylotrophic metabolism in most lineages and in the preservation and repurposing of the WLP as support to fermentation (Adam et al., 2022). Several recent studies revealed the great flexibility of methane‐based metabolisms (Berghuis et al., 2019; Borrel et al., 2019; Evans et al., 2015; Evans et al., 2019; Wang et al., 2019, 2021). It is now clear that these pathways have a modular nature, where different metabolic units can be alternatively combined generating a great metabolic variety (Garcia et al., 2022). In the absence of MTR, the WLP could behave as metabolic module and be combined to other metabolisms beside methanogenesis, such as fermentation. This versatility might explain why the WLP is common among heterotrophic, methane independent, Thermoproteota and Asgardarchaeota (Figure S8) (Spang et al., 2017, 2019).

## CONCLUSION

Our phylogenomic analysis agrees with other studies that place Korarchaeia at the root of the Thermoproteota phylum. Despite traces of a methylotrophic metabolism, Korarchaeia seem to rely only on fermentation of sugars and amino acids. Genetic evidence suggests that deep‐branching Korarchaeia can couple fermentation to acetate production and perform homoacetogenesis. This trait has disappeared in more recently evolved lineages, but it is not clear what ecological drivers determined this loss. The modular nature of the WLP and its propensity combine with other metabolisms is likely the reason for its preservation in Korarchaeia but may also be the reason it is easily lost when environmental conditions make it redundant and energetically unfavourable. Overall, our findings align with the current hypothesis of methylotrophic methanogenesis as the most ancient metabolism, but also suggest that the WLP might be widely used for homoacetogenesis in Archaea. In conclusion, expanding the tree of recently discovered archaeal lineages is key to fully grasping the metabolic potential of the poorly characterized Archaea, and to build a comprehensive evolutionary model for fundamental metabolic pathways such as the MCR‐mediated methanogenesis and the WLP.

## AUTHOR CONTRIBUTIONS

**Francesca Vulcano:** Conceptualization (lead); data curation (supporting); formal analysis (lead); investigation (lead); methodology (lead); visualization (lead); writing – original draft (lead); writing – review and editing (lead). **Petra Hribovšek:** Data curation (equal); writing – original draft (supporting). **Emily Olesin Denny:** Data curation (equal); writing – original draft (supporting). **Ida H. Steen:** Conceptualization (lead); formal analysis (lead); funding acquisition (lead); investigation (lead); methodology (supporting); supervision (lead); writing – original draft (supporting); writing – review and editing (supporting). **Runar Stokke:** Conceptualization (supporting); data curation (lead); formal analysis (lead); funding acquisition (supporting); investigation (supporting); methodology (lead); supervision (supporting); writing – original draft (supporting); writing – review and editing (supporting).

## CONFLICT OF INTEREST STATEMENT

The authors declare that they have no conflict of interest.

## Supporting information


**Data S1.** Supporting information.

**Figure S1.** Comparison between 115‐markers (left) and 42‐markers (right) concatenated phylogenies. Classes within phylum Thermoproteota are indicated in black.

**Figure S2.** Heatmap representing AAI values for species‐level lineages representatives in the Korarchaeia class. OPF8 represents the genome of Ca. Korarchaeum cryptophilum and MDKW represents the genomes of Ca. Methanodesulfokores washburniensis. Values above 65% AAI are in red, values above 95% are in blue. The marine species in the genus‐level lineage Kg_12 (including MAGs: INS_M22_B97, INS_M14_B79, INS_M34_B71, INS_M21_B208) is indicated by a black arrow and shares less than 65% AAI with Ca. Methanodesulfokores washburniensis (MDKW).

**Figure S3.** Map of the metabolic pathways conserved in Korarchaeia. Genes in blue are conserved in all genomes, genes in green are shared by approximately half of the genomes analysed.

**Figure S4.** Distribution of hydrogenases in Korarchaeia genus‐level lineages. The number of genomes in each genus is indicated in parenthesis. The WLP‐encoding genera are surrounded by a black line.

**Figure S5.** Organization of the gene locus of [NiFe]_group_4g hydrogenases in representative genomes of Korarchaeia genera, based on arCOG annotations. [NiFe]_group_4g in HydDB corresponds to Nuo in arCOG. The large and small putative catalytic subunits are in light and dark orange, respectively. The hydrogenases loci for model organisms *Desulfosporosinus orientis* (WP_014183752.1) given in the HydDB database are also reported for comparison. Multicopy hydrogenases are indicated by ** and ''.

**Figure S6.** Distribution of genes for chemolithotrophy in Korarchaeia genus‐level lineages. The WLP‐encoding lineages are surrounded by a black line. Lineages that do not encode any of the screened genes were not reported in the final table. The list of genes considered for the analysis is reported below the heatmap.

**Figure S7.** Model of the putative terminal electron acceptor complex in Kg_3. All ORF belong to the same contig. The cellular location of each subunit was predicted by PSORTb v.3.0 (Yu et al., 2010).

**Figure S8.** Overview of the distribution of the WLP and key genes of methane (alkane)‐based metabolisms in archaeal phyla.

**Table S1A.** Geographic origin and metadata for genomes reconstructed in this study (*Baumberger et al., 2016; **Dahle et al., 2015; ***Stokke et al., 2020).
**Table S1B.** Statistics and classification of Korarchaeia genomes included in this study. The database from which the genomes have been retrieved from is indicated (GEM: Genomes from Earth's Microbiome, Nayfach et al., 2021); NCBI: National Center for Biotechnology Information, Sayers et al., (2020). Bins from this study have been uploaded to NCBI. Bin_ID Database GTDB‐tk classification completeness (%) completeness – CheckM2 (%) contamination (%) contamination – CheckM2 (%) strain heterogeneity (%) GC_content Genome size (Mb) # ambiguous bases # scaffolds # contigs N50 (scaffolds) N50 (contigs) Mean scaffold length (bp) Mean contig length (bp) Longest scaffold (bp) Longest contig (bp) GC std (scaffolds > 1kbp) Coding density Translation table.

**Table S2.** List of genes used for buiding concatenated phylogenies with 42 and 115 markers.

**Table S3.** List of genes accession numbers used to compile the survey presented in Figure 2, through the pipeline presented in Dombrowski et al. (2018).

**Table S4.** List of genes of the WLP identified in the genomes of deep‐branching Korarchaeia. Top hits in the NCBI_COG, arCOG, KO, PFAM, TIGR databases and related e‐values are given for all translated sequences. NCBI taxonomic affiliation is also indicated.

## Data Availability

All MAGs in the study were deposited in NCBI and accession numbers with associated BioProjects and BioSamples are listed in Table S1.
