# Supplementary material for: Potential for homoacetogenesis via the Wood–Ljungdahl pathway in Korarchaeia lineages from marine hydrothermal vents
Source: Environ Microbiol Rep. 2023 May 22;15(6):698–707. doi: 10.1111/1758-2229.13168 (PMC10667645; doi:10.1111/1758-2229.13168)
Supplement: Supplementary file 1 — Data S1. Supporting information. [file EMI4-15-698-s005.docx]

**Potential for acetogenesis via the Wood-Ljungdahl pathway in Korarchaeia lineages from marine hydrothermal vents**

**Supplementary Materials**

**Environmental samples and DNA extraction**

Genomic DNA was extracted from sediment samples and rock pieces collected during several research cruises between 2010 and 2021 (Dahle *et al.,* 2015; Stokke *et al.,* 2020). DNA was extracted with FastDNA^TM^SPIN Kit for Soil (MP Biomedicals, Santa Ana, CA, USA) and sequenced at the NSC Norwegian Sequencing Center in Oslo.

**Sequencing, assembly, binning, and annotation**

Short description for metagenomes INS_M11, INS_M12, INS_M13 and INS_M14:

Genomic DNA was sequenced by Illumina MiSeq 300 paired-end. Filtering and assembly of Illumina MiSeq reads was performed as previously described in (Fredriksen *et al.,* 2019). Metagenome-assembled genomes (MAGs) were reconstructed using the single binning tool MetaBat (Kang *et al.,* 2015). Metagenome INS_M9 were assembled using SPAdes v3.13.0 (Prjibelski *et al.,* 2020) with the –meta option enabled for metagenomic datasets (Nurk *et al.,* 2017), and MAGs were reconstructed using the binning and refinement tool MetaWrap v1.3.2 (Uritskiy *et al.,* 2018) which included the binning algorithms MetaBat v2.12.1 (Kang *et al.,* 2015), Concoct v.1.1.0 (Alneberg *et al.,* 2014) and MaxBin v2.2.7 (Wu *et al.,* 2014).

Short description for metagenomes INS_M19, INS_M20, INS_M21, INS_M22, INS_M29:

Genomic DNA was sequenced with Illumina NovaSeq 6000 150 paired-end using the S4 reagents.

Quality filtering of paired-end reads was performed using fastp v.0.23.2 (Chen *et al.,* 2018) and subsequently assembled into contigs using MEGAHIT v1.2.9 (Li *et al.,* 2015) with a minimum kept contiguous sequence length of 2000 base pairs. Subsequently, MAGs were reconstructed using the binning and refinement tool MetaWrap v1.3.2 (Uritskiy *et al.,* 2018).

Short description for metagenomes 12ROV10_HD34A, 12ROV10_HD34C, 12ROV10_HD34E, 16ROV9_HD21, 16ROV9_HD22, 16ROV9_HD24, 17ROV19_HD25 and 17ROV19_HD4:

Genomic DNA was sequenced with Illumina NovaSeq 6000 150 paired-end using the S4 reagents. Quality filtering of paired-end reads using illumina-utils v2.10 (Eren *et al.,* 2013) codebase script iu-filter-quality-minoche with default settings. Reads were de novo assembled into contigs using MEGAHIT v1.2.9 (Li *et al.,* 2015) with a minimum kept contiguous sequence length of 2000 base pairs. Binning was then performed using the wrapper script anvi-cluster-contigs in Anvi’o v7 (Eren *et al.,* 2015) using the following binning software: Concoct v.1.1.0, MetaBat v2.12.1, and MaxBin v2.2.7. DASTOOL v1.1.2 (Sieber *et al.,* 2018) was used for bin refinement and picking of a final optimized set of non-redundant bins gleaned from individual assemblies.

In addition to MAGs constructed in the current study, reference genomes were downloaded from the Assembly database at NCBI (March 2022). Contamination and completeness of the MAGs from this study and references were assessed with CheckM v1.0.7 (Parks *et al.,* 2015) and CheckM2 (Chklovski et al., 2022) (Supplementary Table 5). All MAGs with estimated completeness of >70% based on CheckM2 were functionally annotated by with the automated pipeline by Dombrowski *et al.,* 2020. This performs individual database searches against Prokka, NCBI COG, arCOG, KEGG, Pfam, TIGR, CAZy, HydDB. For metabolic reconstruction presence/absence of genes of interest was determined in all Korarchaeia genomes, mainly based on KO and arCOG annotations. Hydrogenases were identified using HydDB (Søndergaard et., al 2016). The subunits of the putative terminal electron acceptor complex in Kg_3 was identified using Pfam annotations (Supplementary Figure 4).

Metagenomic computations were performed on resources provided by Sigma2 - the National Infrastructure for High Performance Computing and Data Storage in Norway.

**Taxonomic classification, phylogenetic and phylogenomic analysis**

Classification of MAGs was performed using the GTDB toolkit (GTDB-Tk) (Chaumeil *et al.,* 2020) and the GTDB version R06-RS202 (Parks *et al.,* 2018; Parks *et al.,* 2021).

Phylogenomic analysis was performed on 97 Korarchaeia genomes and 254 archaeal genomes as references. Amino acid sequences from 42 and 115 selected single-copy marker genes (Supplementary Table 2) were identified and extracted using the HMM source GTDB_ar122_r202 (from GTDB release 202) in Anvi’o (Eren *et al.,* 2021). Genes were selected as markers when present in at least 75% of the Korarchaeia MAGs and not present in more than two copies per MAG. The single-copy marker genes sequences were aligned using MAFFT L-INS-i v7.397 (2018/Apr/16) (Katoh*,* 2002), trimmed with TrimAL (TrimAL v 1.4. rev15, -gappyout) (Capella-Gutiérrez *et al.,* 2009) and concatenated with catfasta2phyml (https://github.com/nylander/catfasta2phyml/blob/master/catfasta2phyml.pl). A maximum-likelihood tree of the concatenated sequences was calculated with IQ-TREE (multicore version 2.0.3) (Nguyen *et al.,* 2015) with LG+F+R10 model (automatic model selection -m MFP), 1000 bootstraps and ultrafast bootstrapping. EzAAI (Kim *et al.,* 2021) was used to calculate Average Amino Acid Identity (AAI). A cut-off of 65% AAI was used for genus-level clustering of genomes (Goris et al., 2007; Konstantinidis, 2017).
