# Supplementary figures and images for: Potential for homoacetogenesis via the Wood–Ljungdahl pathway in Korarchaeia lineages from marine hydrothermal vents
Source: Environ Microbiol Rep. 2023 May 22;15(6):698–707. doi: 10.1111/1758-2229.13168 (PMC10667645; doi:10.1111/1758-2229.13168)

Tree scale: 1

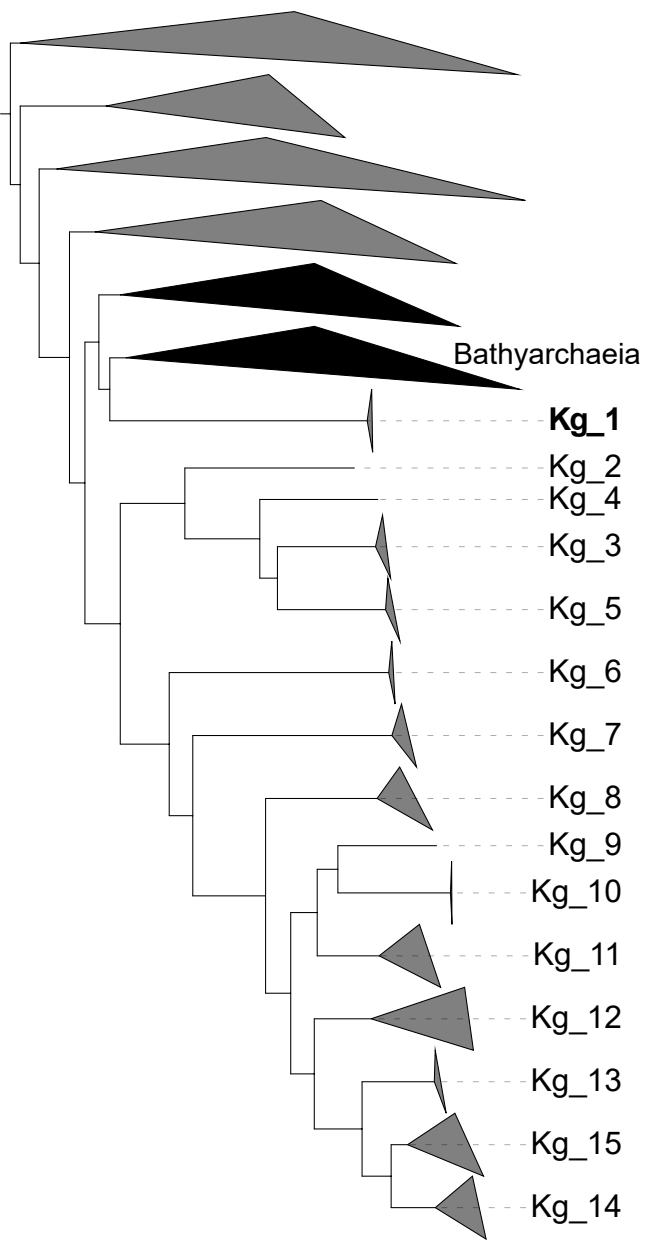

115 markers

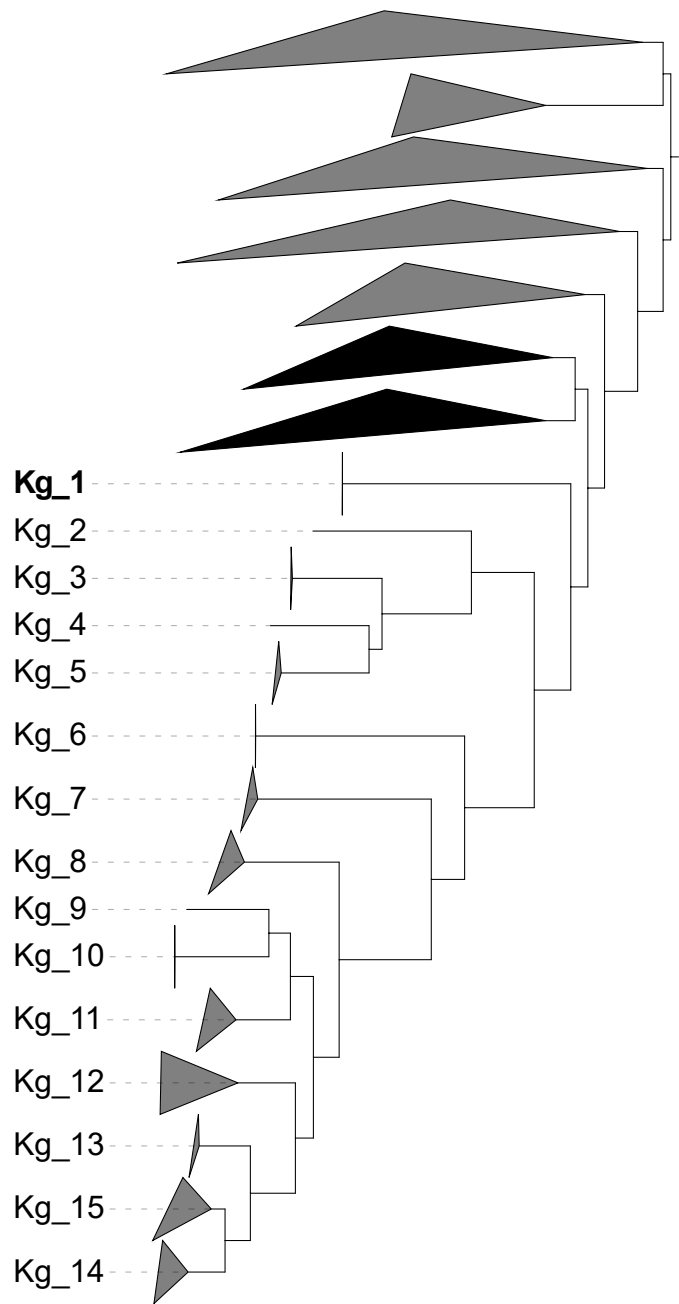

42 markers

Supplement: Supplementary file 2 — Figure S1. Comparison between 115‐markers (left) and 42‐markers (right) concatenated phylogenies. Classes within phylum Thermoproteota are indicated in black. [file EMI4-15-698-s011.pdf]

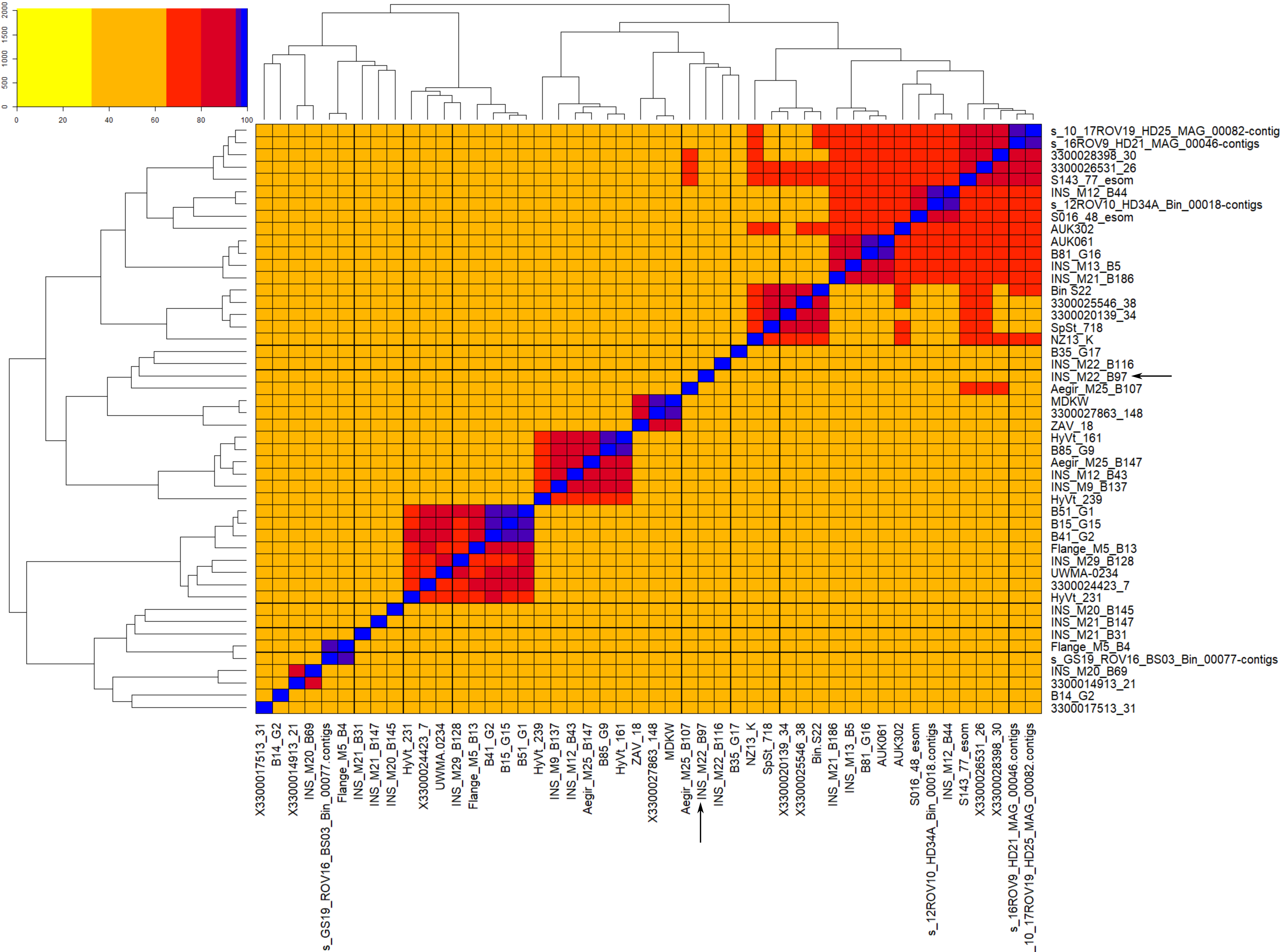

Supplement: Supplementary file 3 — Figure S2. Heatmap representing AAI values for species‐level lineages representatives in the Korarchaeia class. OPF8 represents the genome of Ca. Korarchaeum cryptophilum and MDKW represents the genomes of Ca. Methanodesulfokores washburniensis. Values above 65% AAI are in red, values above 95% are in blue. The marine species in the genus‐level lineage Kg_12 (including MAGs: INS_M22_B97, INS_M14_B79, INS_M34_B71, INS_M21_B208) is indicated by a black arrow and shares less than 65% AAI with Ca. Methanodesulfokores washburniensis (MDKW). [file EMI4-15-698-s010.pdf]

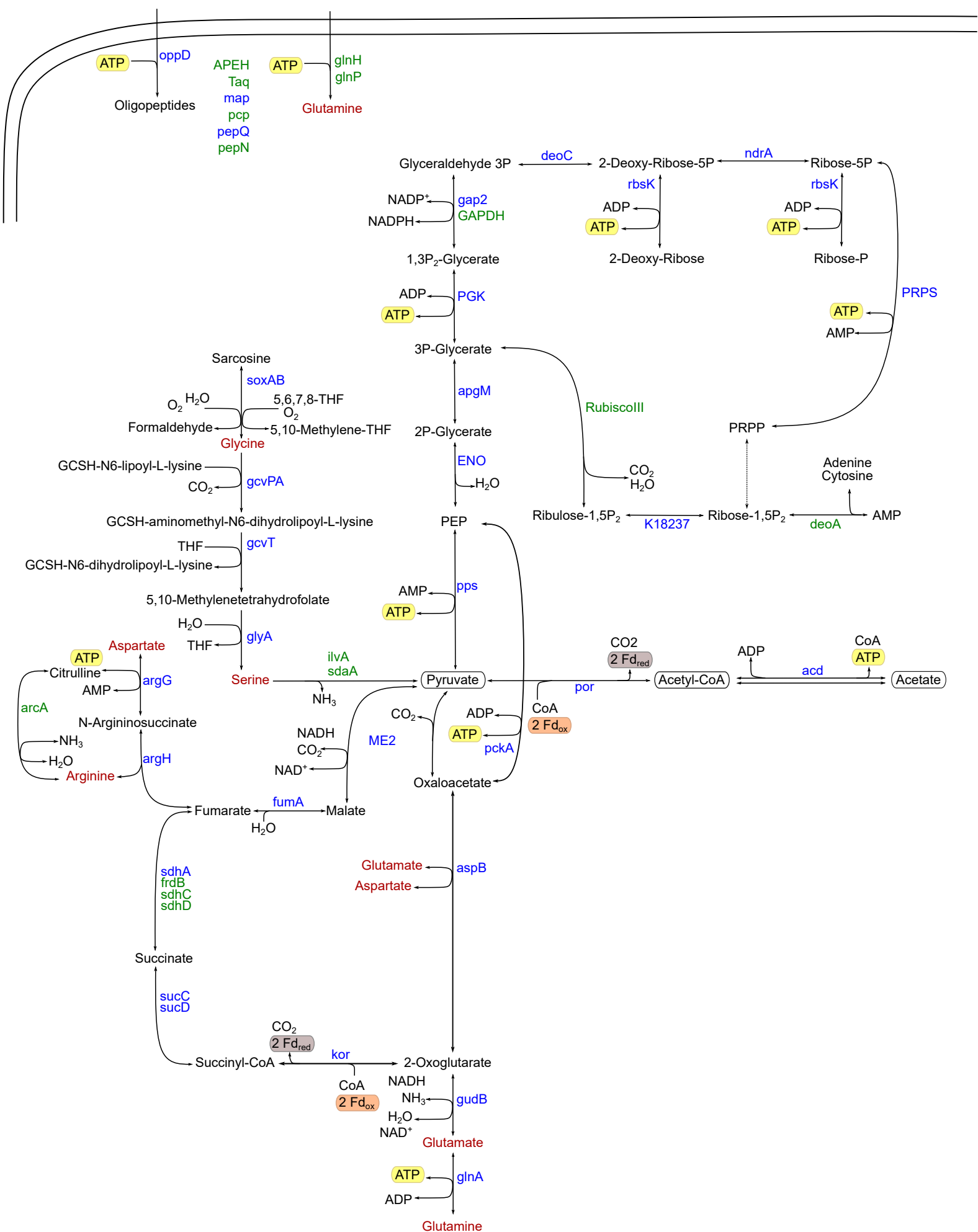

Supplement: Supplementary file 4 — Figure S3. Map of the metabolic pathways conserved in Korarchaeia. Genes in blue are conserved in all genomes, genes in green are shared by approximately half of the genomes analysed. [file EMI4-15-698-s006.pdf]

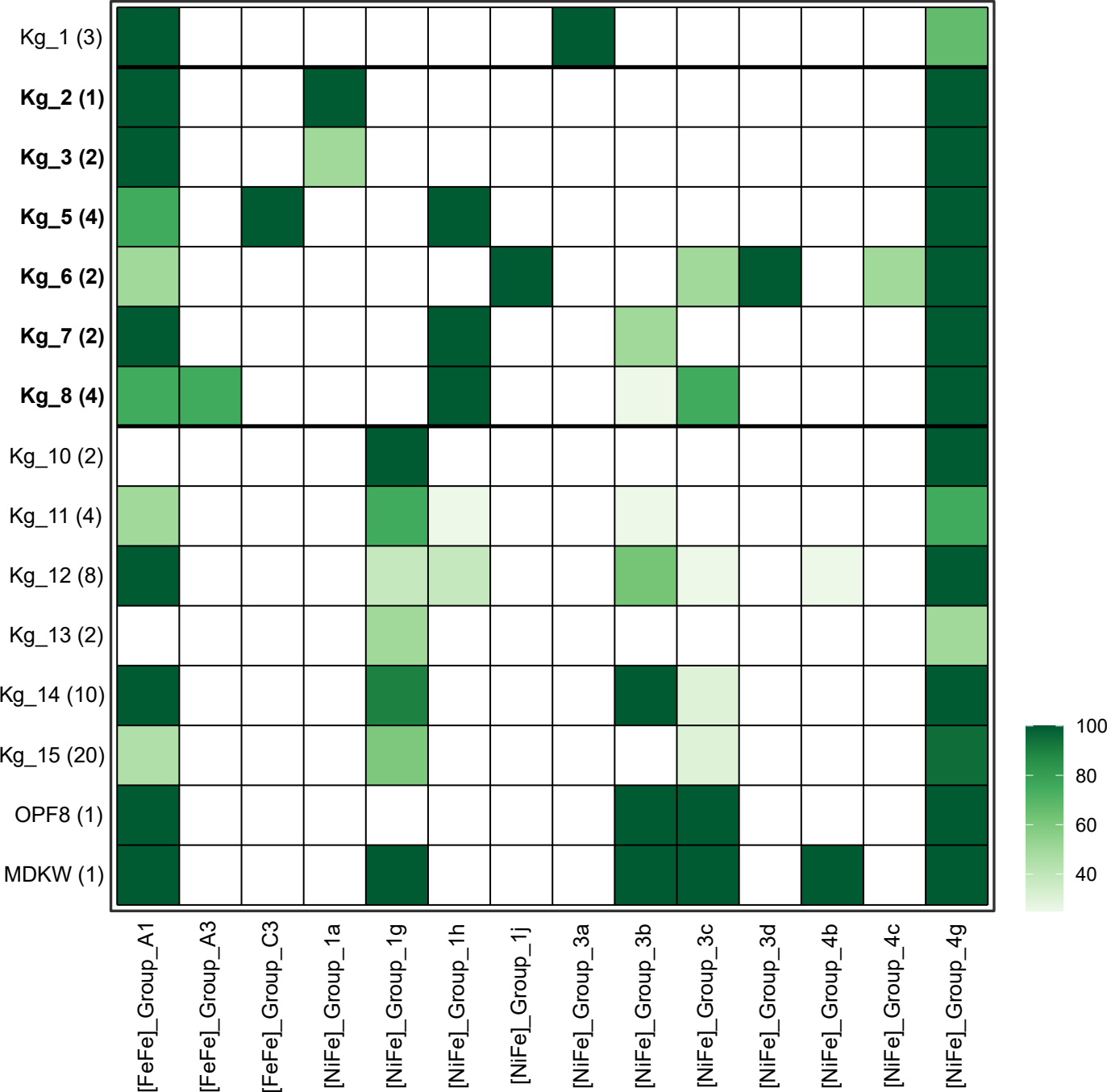

Supplement: Supplementary file 5 — Figure S4. Distribution of hydrogenases in Korarchaeia genus‐level lineages. The number of genomes in each genus is indicated in parenthesis. The WLP‐encoding genera are surrounded by a black line. [file EMI4-15-698-s008.pdf]

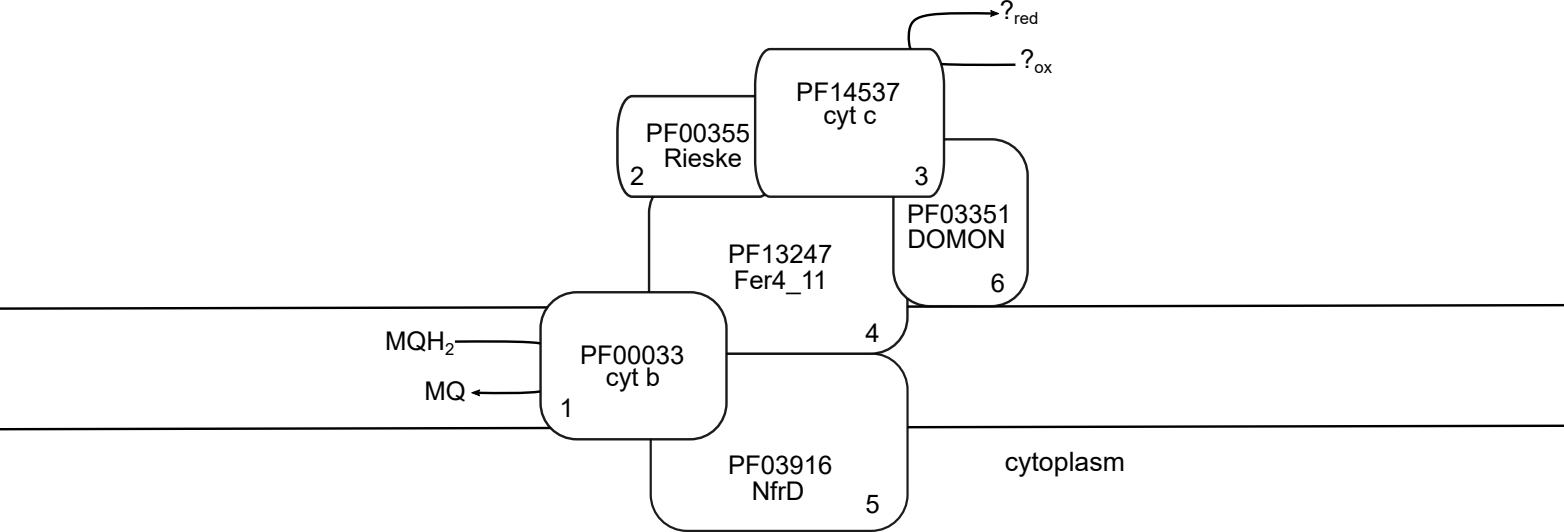

Supplement: Supplementary file 8 — Figure S7. Model of the putative terminal electron acceptor complex in Kg_3. All ORF belong to the same contig. The cellular location of each subunit was predicted by PSORTb v.3.0 (Yu et al., 2010). [file EMI4-15-698-s004.pdf]

● TACK-MCR  
 ● WLP  
 ● MTR  
 ● Euryarchaeota-MCR  
 ● ACR  
 ● Predicted heterotrophs

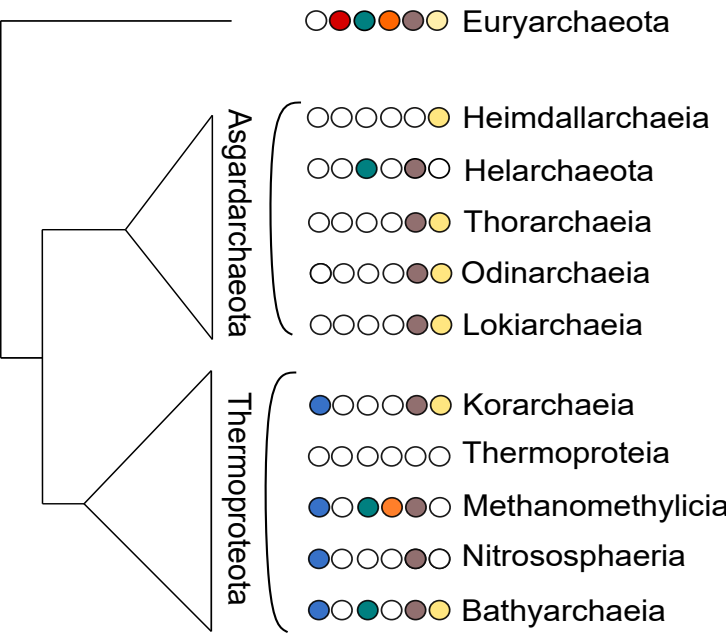

Supplement: Supplementary file 9 — Figure S8. Overview of the distribution of the WLP and key genes of methane (alkane)‐based metabolisms in archaeal phyla. [file EMI4-15-698-s002.pdf]
