# Supplementary material for: Potential for homoacetogenesis via the Wood–Ljungdahl pathway in Korarchaeia lineages from marine hydrothermal vents
Source: Environ Microbiol Rep. 2023 May 22;15(6):698–707. doi: 10.1111/1758-2229.13168 (PMC10667645; doi:10.1111/1758-2229.13168)
Supplement: Supplementary file 6 — Figure S5. Organization of the gene locus of [NiFe]_group_4g hydrogenases in representative genomes of Korarchaeia genera, based on arCOG annotations. [NiFe]_group_4g in HydDB corresponds to Nuo in arCOG. The large and small putative catalytic subunits are in light and dark orange, respectively. The hydrogenases loci for model organisms Desulfosporosinus orientis (WP_014183752.1) given in the HydDB database are also reported for comparison. Multicopy hydrogenases are indicated by ** and ''. [file EMI4-15-698-s012.pdf]

[NiFe]_group_4g

	Small subunit	Large subunit ([NiFe]-group-4g)									
D. orientis	**nuoB**	**nuoD**	nuoC	nuoH	HP	HP	nuoL	antiporter			
	Large subunit ([NiFe]-group-4g)										
Kg_2	**nuoD**	HypF									
Kg_3	**nuoB**	nuoC	**nuoD**	nuoH	nuoI	nuoJ	mnhB	nuoK	nuoL		
Kg_5	nuoA	**nuoB**	nuoC	**nuoD**	nuoH	nuoI	nuoJ	antiporter	nuoK	nuoL	
Kg_6	nuoA	**nuoB**	nuoC	**nuoD**	nuoH	nuoI	nuoJ	nuoK	nuoM	nuoL	
Kg_7	nuoA	**nuoB**	nuoC	**nuoD**	nuoH	nuoI	nuoJ	nuoK	nuoM	nuoL	nuoN
Kg_8	nuoA	**nuoB**	nuoC	**nuoD**	nuoH	nuoI	nuoJ	nuoK	nuoM	nuoL	nuoN
Kg_10	nuoA	**nuoB**	nuoC	**nuoD**	nuoH	nuoI	nuoJ	nuoK	nuoM	nuoL	nuoN
Kg_11	nuoA	**nuoB**	nuoC	**nuoD**	nuoH	nuoI	nuoJ	nuoK	nuoM	nuoL	nuoN
Kg_12*	nuoA	**nuoB**	**nuoD**	nuoH	nuoI	nuoJ	nuoK		nuoM	nuoN	
Kg_12**	nuoA	putative small sub.	nuoC	**nuoD**	nuoH	nuoI	nuoJ	nuoK		nuoM	
Kg_14'	nuoA	**nuoB**	nuoC	**nuoD**	nuoH	nuoI	nuoJ	UP			
Kg_14''	nuoA	putative small sub.	nuoC	**nuoD**	nuoH	nuoI	nuoJ	nuoK	nuoM	nuoL	nuoN
