# Supplementary material for: Potential for homoacetogenesis via the Wood–Ljungdahl pathway in Korarchaeia lineages from marine hydrothermal vents
Source: Environ Microbiol Rep. 2023 May 22;15(6):698–707. doi: 10.1111/1758-2229.13168 (PMC10667645; doi:10.1111/1758-2229.13168)
Supplement: Supplementary file 7 — Figure S6. Distribution of genes for chemolithotrophy in Korarchaeia genus‐level lineages. The WLP‐encoding lineages are surrounded by a black line. Lineages that do not encode any of the screened genes were not reported in the final table. The list of genes considered for the analysis is reported below the heatmap. [file EMI4-15-698-s003.pdf]

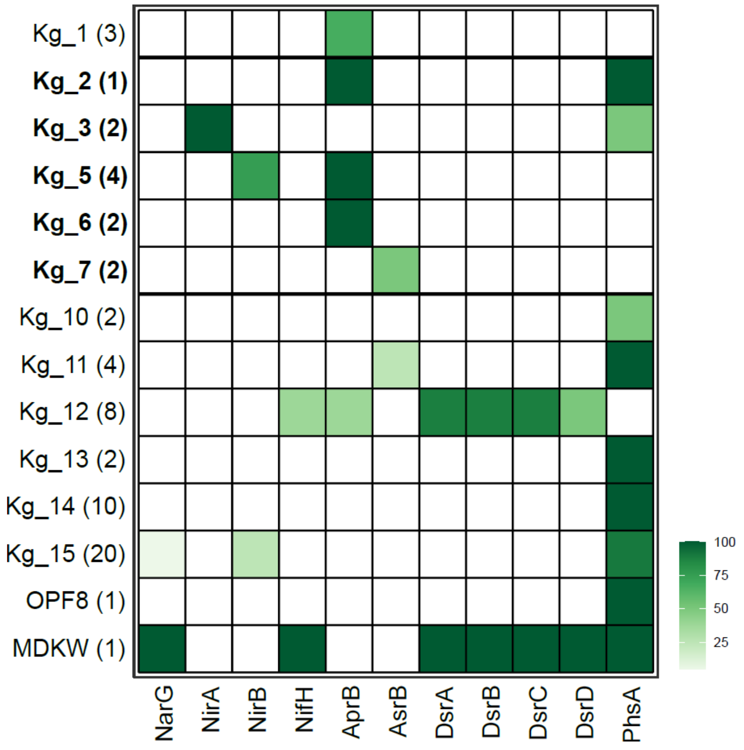

GeneID	Gene	Gene_description
K02567	NapA	Periplasmic nitrate reductase
K02568	NapB	Periplasmic nitrate reductase, electron transfer subunit
K02569	NapC	Cytochrome c-type protein NapC
arCOG01497	NarG	Nitrate reductase alpha subunit (NarG)
K00371	NarH	Respiratory nitrate reductase subunit beta
K00366	NirA	Ferredoxin nitrite reductase protein
K00362	NirB	Nitrite reductase (NADH) large subunit
K00368	NirK	Copper-containing nitrite reductase
arCOG00598	NifD	Nitrogenase molybdenum-iron protein alpha chain
arCOG00590	NifH	Nitrogenase iron protein1
arCOG00594	NifK	Nitrogenase molybdenum-iron protein beta chain
arCOG00591	Nif	Nitrogenase
arCOG00592	Nif	Nitrogenase
arCOG00593	Nif	Nitrogenase
arCOG00595	Nif	Nitrogenase
K04561	NorB	nitric oxide reductase subunit B [EC:1.7.2.5]
K02305	NorC	nitric oxide reductase subunit C
K00376	NosZ	nitrous-oxide reductase [EC:1.7.2.4]
K00394	AprA	adenylylsulfate reductase, subunit A [EC:1.8.99.2]
K00395	AprB	adenylylsulfate reductase, subunit B [EC:1.8.99.2]
K16950	AsrA	anaerobic sulfite reductase subunit A
K16951	AsrB	anaerobic sulfite reductase subunit B
arCOG02057	DsrA	Sulfite reductase, dissimilatory-type subunit alpha
arCOG02058	DsrA	Dissimilatory sulfite reductase alpha subunit
K11180	DsrA	Dissimilatory sulfite reductase alpha subunit
K11181	DsrB	Dissimilatory sulfite reductase beta subunit
K23077	DsrC	Sulfite reductase, dissimilatory-type subunit gamma
arCOG10384	DsrD	Protein DsrD
K08352	PhsA	thiosulfate reductase / polysulfide reductase chain A
K08353	PhsB	Thiosulfate reductase electron transfer subunit PhsB
K08354	PhsC	Thiosulfate reductase cytochrome B subunit PhsC
