# Supplementary material for: Potential for homoacetogenesis via the Wood–Ljungdahl pathway in Korarchaeia lineages from marine hydrothermal vents
Source: Environ Microbiol Rep. 2023 May 22;15(6):698–707. doi: 10.1111/1758-2229.13168 (PMC10667645; doi:10.1111/1758-2229.13168)
Supplement: Supplementary file 10 — Table S1A. Geographic origin and metadata for genomes reconstructed in this study (*Baumberger et al., 2016; **Dahle et al., 2015; ***Stokke et al., 2020). Table S1B. Statistics and classification of Korarchaeia genomes included in this study. The database from which the genomes have been retrieved from is indicated (GEM: Genomes from Earth's Microbiome, Nayfach et al., 2021); NCBI: National Center for Biotechnology Information, Sayers et al., (2020). Bins from this study have been uploaded to NCBI. Bin_ID Database GTDB‐tk classification completeness (%) completeness – CheckM2 (%) contamination (%) contamination – CheckM2 (%) strain heterogeneity (%) GC_content Genome size (Mb) # ambiguous bases # scaffolds # contigs N50 (scaffolds) N50 (contigs) Mean scaffold length (bp) Mean contig length (bp) Longest scaffold (bp) Longest contig (bp) GC std (scaffolds > 1kbp) Coding density Translation table. [file EMI4-15-698-s013.pdf]

Supplementary Table 1A. Geographic origin and metadata for genomes reconstructed in this study. (*Baumberger et al., 2016; **Dahle et al., 2015; ***Stokke et al., 2020)

**Bin_ID**	**Location**	**Type of sample**	**Description**	BioProject acc.	BioSample acc.
12ROV10_HD34A_Bin_00018	Jan Mayen- Soria Moria	High-temperature smoker - chimney	NA	PRJNA949439	SAMN34520783
12ROV10_HD34C_MAG_00048	Jan Mayen- Soria Moria	High-temperature smoker - chimney	NA	PRJNA949439	SAMN34510431
12ROV10_HD34E_MAG_00031	Jan Mayen- Soria Moria	High-temperature smoker - chimney	NA	PRJNA949439	SAMN34510483
16ROV9_HD21_MAG_00046	Ægir Vent Field	High-temperature smoker - chimney	NA	PRJNA949439	SAMN34511026
16ROV9_HD22_Bin_00127	Ægir Vent Field	High-temperature smoker - chimney	NA	PRJNA949439	SAMN34511145
16ROV9_HD24_Bin_00133	Ægir Vent Field	High-temperature smoker - chimney	NA	PRJNA949439	SAMN34510853
16ROV9_HD24_MAG_00031	Ægir Vent Field	High-temperature smoker - chimney	NA	PRJNA949439	SAMN34511170
17ROV19_HD25_MAG_00082	Loki's Castle Vent Field*	High-temperature smoker - chimney	NA	PRJNA803957	SAMN34377303
17ROV19_HD25_MAG_00106	Loki's Castle Vent Field*	High-temperature smoker - chimney	NA	PRJNA803957	SAMN34377303
17ROV19_HD4_Bin_00097	Loki's Castle Vent Field*	High-temperature smoker - chimney	NA	PRJNA803957	SAMN34376232
Aegir_M25_B107	Ægir Vent Field	High-temperature smoker - chimney	NA	PRJNA881934	SAMN31014040
Aegir_M25_B147	Ægir Vent Field	High-temperature smoker - chimney	NA	PRJNA881934	SAMN31014041
Aegir_M25_B233	Ægir Vent Field	High-temperature smoker - chimney	NA	PRJNA881934	SAMN31014042
Flange_M5_B13	Jan Mayen- Soria Moria**	High-temperature smoker -flange structure	70°C - 72°C	PRJNA785783	SAMN31014043
Flange_M5_B24	Jan Mayen- Soria Moria**	High-temperature smoker -flange structure	70°C - 72°C	PRJNA785783	SAMN31014044
Flange_M5_B4	Jan Mayen- Soria Moria**	High-temperature smoker -flange structure	70°C - 72°C	PRJNA785783	SAMN31014045
GS19_ROV16_BS03_Bin_00077	Fåvne Vent Field	High-temperature smoker - chimney	NA	PRJNA949439	SAMN34510739
INS_M11_B49	Jan Mayen- Bruse Vent Field***	Hydrothermal sediments (13.5 - 16 cmbsf)	approx. 49°C - 74°C	PRJNA801110	SAMN31014046
INS_M12_B43	Jan Mayen- Bruse Vent Field***	Hydrothermal sediments (4.5 - 7 cmbsf)	approx. 0°C - 24°C	PRJNA801110	SAMN31014047
INS_M12_B44	Jan Mayen- Bruse Vent Field***	Hydrothermal sediments (4.5 - 7 cmbsf)	approx. 0°C - 24°C	PRJNA801110	SAMN31014048
INS_M12_B58	Jan Mayen- Bruse Vent Field***	Hydrothermal sediments (4.5 - 7 cmbsf)	approx. 0°C - 24°C	PRJNA801110	SAMN31014049
INS_M13_B5	Jan Mayen- Bruse Vent Field***	Hydrothermal sediments (9 - 11.5 cmbsf)	approx. 25°C - 48°C	PRJNA801110	SAMN31014050
INS_M14_B79	Jan Mayen- Bruse Vent Field***	Hydrothermal sediments (4.5 - 7 cmbsf)	approx. 0°C - 24°C	PRJNA801110	SAMN31014051
INS_M19_B79	Jan Mayen- Bruse Vent Field***	Hydrothermal sediments (13.5 - 16 cmbsf)	approx. 49°C - 74°C	PRJNA801110	SAMN31014052
INS_M20_B145	Jan Mayen- Bruse Vent Field***	Hydrothermal sediments (9 - 11.5 cmbsf)	approx. 25°C - 48°C	PRJNA801110	SAMN31014053
INS_M20_B155	Jan Mayen- Bruse Vent Field***	Hydrothermal sediments (9 - 11.5 cmbsf)	approx. 25°C - 48°C	PRJNA801110	SAMN31014054
INS_M20_B204	Jan Mayen- Bruse Vent Field***	Hydrothermal sediments (9 - 11.5 cmbsf)	approx. 25°C - 48°C	PRJNA801110	SAMN31014055
INS_M20_B56	Jan Mayen- Bruse Vent Field***	Hydrothermal sediments (9 - 11.5 cmbsf)	approx. 25°C - 48°C	PRJNA801110	SAMN31014056
INS_M20_B69	Jan Mayen- Bruse Vent Field***	Hydrothermal sediments (9 - 11.5 cmbsf)	approx. 25°C - 48°C	PRJNA801110	SAMN31014057
INS_M20_B71	Jan Mayen- Bruse Vent Field***	Hydrothermal sediments (9 - 11.5 cmbsf)	approx. 25°C - 48°C	PRJNA801110	SAMN31014058
INS_M21_B147	Jan Mayen- Bruse Vent Field***	Hydrothermal sediments (13.5 - 16 cmbsf)	approx. 49°C - 74°C	PRJNA801110	SAMN31014059
INS_M21_B149	Jan Mayen- Bruse Vent Field***	Hydrothermal sediments (13.5 - 16 cmbsf)	approx. 49°C - 74°C	PRJNA801110	SAMN31014060
INS_M21_B166	Jan Mayen- Bruse Vent Field***	Hydrothermal sediments (13.5 - 16 cmbsf)	approx. 49°C - 74°C	PRJNA801110	SAMN31014061
INS_M21_B183	Jan Mayen- Bruse Vent Field***	Hydrothermal sediments (13.5 - 16 cmbsf)	approx. 49°C - 74°C	PRJNA801110	SAMN31014062
INS_M21_B186	Jan Mayen- Bruse Vent Field***	Hydrothermal sediments (13.5 - 16 cmbsf)	approx. 49°C - 74°C	PRJNA801110	SAMN31014063
INS_M21_B208	Jan Mayen- Bruse Vent Field***	Hydrothermal sediments (13.5 - 16 cmbsf)	approx. 49°C - 74°C	PRJNA801110	SAMN31014064
INS_M21_B31	Jan Mayen- Bruse Vent Field***	Hydrothermal sediments (13.5 - 16 cmbsf)	approx. 49°C - 74°C	PRJNA801110	SAMN31014065
INS_M21_B37	Jan Mayen- Bruse Vent Field***	Hydrothermal sediments (13.5 - 16 cmbsf)	approx. 49°C - 74°C	PRJNA801110	SAMN31014066
INS_M21_B77	Jan Mayen- Bruse Vent Field***	Hydrothermal sediments (13.5 - 16 cmbsf)	approx. 49°C - 74°C	PRJNA801110	SAMN31014067
INS_M22_B116	Jan Mayen- Bruse Vent Field***	Hydrothermal sediments (13.5 - 16 cmbsf)	approx. 49°C - 74°C	PRJNA801110	SAMN31014068

INS_M22_B125	Jan Mayen- Bruse Vent Field***	Hydrothermal sediments (13.5 - 16 cmbsf)	approx. 49°C - 74°C	PRJNA801110	SAMN31014069
INS_M22_B44	Jan Mayen- Bruse Vent Field***	Hydrothermal sediments (13.5 - 16 cmbsf)	approx. 49°C - 74°C	PRJNA801110	SAMN31014070
INS_M22_B49	Jan Mayen- Bruse Vent Field***	Hydrothermal sediments (13.5 - 16 cmbsf)	approx. 49°C - 74°C	PRJNA801110	SAMN31014071
INS_M22_B97	Jan Mayen- Bruse Vent Field***	Hydrothermal sediments (13.5 - 16 cmbsf)	approx. 49°C - 74°C	PRJNA801110	SAMN31014072
INS_M22_B98	Jan Mayen- Bruse Vent Field***	Hydrothermal sediments (13.5 - 16 cmbsf)	approx. 49°C - 74°C	PRJNA801110	SAMN31014073
INS_M29_B128	Jan Mayen- Bruse Vent Field***	Hydrothermal sediments (4.5 - 7 cmbsf)	approx. 0°C - 24°C	PRJNA881940	SAMN31014074
INS_M34_B48	Jan Mayen- Bruse Vent Field***	Hydrothermal sediments (4.5 - 7 cmbsf)	approx. 0°C - 24°C	PRJNA801110	SAMN31014075
INS_M34_B71	Jan Mayen- Bruse Vent Field***	Hydrothermal sediments (4.5 - 7 cmbsf)	approx. 0°C - 24°C	PRJNA801110	SAMN31014076
INS_M9_B137	Jan Mayen- Bruse Vent Field***	Hydrothermal sediments (9 - 11.5 cmbsf)	approx. 25°C - 48°C	PRJNA801110	SAMN31014077
INS_M9_B20	Jan Mayen- Bruse Vent Field***	Hydrothermal sediments (9 - 11.5 cmbsf)	approx. 25°C - 48°C	PRJNA801110	SAMN31014078

Supplementary Table 1B. Statistics and classification of Korarchaeia genomes included in this study. The database from which the genomes have been retrieved from is indicated (GEM: Genomes from Earth's Microbiome, Nayfach et al., 2021); NCBI: National Center for Biotechnology Information, Sayers et al., 2022). Bins from this studiy have been uploaded to NCBI.

Bin_ID	Database	GTDB-tk classification	completeness (%)	completeness - CheckM2 (%)	contamination (%)	contamination - CheckM2 (%)	strain heterogeneity (%)	GC_content	Genome size (Mb)	# ambiguous bases	# scaffolds	# contigs	N50 (scaffolds)	N50 (contigs)	Mean scaffold length (bp)	Mean contig length (bp)	Longest scaffold (bp)	Longest contig (bp)	GC std (scaffolds > 1kbp)	Coding density	Translation table
3300005275_7	GEM	d__Archaea;p__Thermoproteota;c__Korarchaeia;o__Korarchaeales;f__Korarchaeaceae;g__Korarchaeum;s__Korarchaeum sp011056255	91.5	92.9	1.9	1.3	0	47.8	1.5	551	82	82	26065	26058	17936	17929	133468	133446	1.25	90.73	11
3300009503_170	GEM	d__Archaea;p__Thermoproteota;c__Korarchaeia;o__Korarchaeales;f__Korarchaeaceae;g__Korarchaeum;s__Korarchaeum cryptofilum	89.3	88.8	1.4	1.5	50	49.2	1.3	0	130	130	12638	12638	9948	9948	85597	85597	1.72	93.13	11
3300014887_59	GEM	d__Archaea;p__Thermoproteota;c__Korarchaeia;o__Korarchaeales;f__B14-G2;g__B14-G2;s__B14-G2 sp003661385	79.0	51.7	0.9	0.2	0	48.6	0.5	0	52	52	10089	10089	8892	8892	39125	39125	1.51	93.1	11
3300014913_21	GEM	d__Archaea;p__Thermoproteota;c__Korarchaeia;o__Korarchaeales;f__;g__;s__	68.9	70.1	3.7	9	0	45.7	1.6	0	116	116	21571	21571	13556	13556	82556	82556	2.37	90.76	11
3300014914_102	GEM	d__Archaea;p__Thermoproteota;c__Korarchaeia;o__Korarchaeales;f__B14-G2;g__B14-G2;s__B14-G2 sp003661385	76.2	43.3	0.9	0.5	0	48.2	0.4	0	29	29	16400	16400	12420	12420	46451	46451	1.19	92.71	11
3300014914_45	GEM	d__Archaea;p__Thermoproteota;c__Korarchaeia;o__DRAE01;f__DRAE01;g__DRAE01;s__DRAE01 sp011041895	68.2	70.4	2.8	5.2	0	40.9	1.0	0	87	87	15731	15731	11109	11109	60373	60373	1.18	94.86	11
3300017513_31	GEM	d__Archaea;p__Thermoproteota;c__Korarchaeia;o__DRAE01;f__DRAE01;g__DRAE01;s__DRAE01 sp011041895	87.9	81.5	0.9	0.1	0	41	1.0	0	54	54	77866	77866	19248	19248	134711	134711	1.17	93.9	11
3300020139_34	GEM	d__Archaea;p__Thermoproteota;c__Korarchaeia;o__Korarchaeales;f__Korarchaeaceae;g__Korarchaeum;s__Korarchaeum sp011056255	93.4	100	3.7	0.7	0	47.8	1.5	0	68	68	47469	47469	22587	22587	261151	261151	1.42	90.17	11
3300021498_7	GEM	d__Archaea;p__Thermoproteota;c__Korarchaeia;o__;f__;g__;s__	61.2	74.1	0.0	1	0	39.9	1.2	0	205	205	6143	6143	5749	5749	17850	17850	1.29	88.76	11
3300024423_7	GEM	d__Archaea;p__Thermoproteota;c__Korarchaeia;o__Korarchaeales;f__QMVU01;g__QMVU01;s__	85.9	90.2	3.3	3.4	40	42.6	1.8	0	58	58	58509	58509	31400	31400	138493	138493	1.44	86.52	11
3300025546_38	GEM	d__Archaea;p__Thermoproteota;c__Korarchaeia;o__Korarchaeales;f__Korarchaeaceae;g__Korarchaeum;s__Korarchaeum cryptofilum	92.9	98.1	2.8	1.7	0	49	1.6	0	93	93	30055	30055	17339	17339	93637	93637	1.73	90.84	11
3300026488_20	GEM	d__Archaea;p__Thermoproteota;c__Korarchaeia;o__;f__;g__;s__	69.0	72.7	1.4	0.1	0	50.3	0.9	0	89	89	13472	13472	9852	9852	35040	35040	1.15	95.38	11
3300026531_26	GEM	d__Archaea;p__Thermoproteota;c__Korarchaeia;o__Korarchaeales;f__Korarchaeaceae;g__;s__	94.3	97.6	1.9	1.3	0	54.9	1.6	0	99	99	24992	24992	16388	16388	71293	71293	2.31	92.45	11
3300027863_148	GEM	d__Archaea;p__Thermoproteota;c__Korarchaeia;o__Korarchaeales;f__Korarchaeaceae;g__Methanodesulfokores;s__Methanodesulfokores washburnensis	92.1	92.5	1.9	1	0	43.3	1.7	0	154	154	17215	17215	10997	10997	140237	140237	1.58	90.49	11
3300027863_171	GEM	d__Archaea;p__Thermoproteota;c__Korarchaeia;o__Korarchaeales;f__Korarchaeaceae;g__Korarchaeum;s__Korarchaeum cryptofilum	88.7	99.1	1.9	1.2	0	48.9	1.5	0	97	97	33282	33282	15689	15689	120610	120610	1.96	91.77	11
3300028398_30	GEM	d__Archaea;p__Thermoproteota;c__Korarchaeia;o__Korarchaeales;f__Korarchaeaceae;g__;s__	94.3	99.7	2.8	2.5	0	52.9	1.9	0	11	11	504152	504152	173770	173770	883081	883081	1.73	89.65	11
AUK061	NCBI	d__Archaea;p__Thermoproteota;c__Korarchaeia;o__Korarchaeales;f__Korarchaeaceae;g__;s__	80.3	78.9	2.8	2.6	33	45.1	1.5	0	230	230	7634	7634	6369	6369	30140	30140	1.7	89.83	11
AUK302	NCBI	d__Archaea;p__Thermoproteota;c__Korarchaeia;o__Korarchaeales;f__Korarchaeaceae;g__;s__	64.8	58.7	1.9	1.8	0	50.8	1.0	0	218	218	4692	4692	4491	4491	15391	15391	2.19	89.79	11
B10_G17	NCBI	d__Archaea;p__Thermoproteota;c__Korarchaeia;o__Korarchaeales;f__B14-G2;g__B14-G2;s__B14-G2 sp003661385	89.5	82.5	3.3	0.8	20	52.8	1.7	0	275	275	7166	7166	6091	6091	39125	39125	3.26	90.86	11
B14_G2	NCBI	d__Archaea;p__Thermoproteota;c__Korarchaeia;o__Korarchaeales;f__B14-G2;g__B14-G2;s__B14-G2 sp003661385	90.7	91.1	2.3	1.9	0	52.8	1.7	0	206	206	9711	9711	8445	8445	48614	48614	3.12	90.72	11
B15_G15	NCBI	d__Archaea;p__Thermoproteota;c__Korarchaeia;o__Korarchaeales;f__QMVU01;g__QMVU01;s__QMVU01 sp003661365	77.8	78.5	2.8	1.9	25	43.6	1.8	0	255	255	8833	8833	7151	7151	40943	40943	2.06	89.89	11
B35_G17	NCBI	d__Archaea;p__Thermoproteota;c__Korarchaeia;o__Korarchaeales;f__Korarchaeaceae;g__;s__	65.0	64.4	6.5	6.2	44	65	0.8	0	199	199	4098	4098	4192	4192	21767	21767	2.71	93.74	11
B41_G2	NCBI	d__Archaea;p__Thermoproteota;c__Korarchaeia;o__Korarchaeales;f__QMVU01;g__QMVU01;s__	57.4	66.7	8.6	10	20	44	1.5	0	337	337	4577	4577	4436	4436	26389	26389	2.79	91.08	11
B51_G1	NCBI	d__Archaea;p__Thermoproteota;c__Korarchaeia;o__Korarchaeales;f__QMVU01;g__QMVU01;s__QMVU01 sp003661365	75.4	68.2	6.5	1.6	33	43.9	1.7	0	390	390	4675	4675	4393	4393	24841	24841	4.35	90.54	11
B68_G1	NCBI	d__Archaea;p__Thermoproteota;c__Korarchaeia;o__Korarchaeales;f__;g__;s__	63.7	65.2	2.8	0.9	33	42.3	1.3	0	229	229	7239	7239	5528	5528	82556	82556	6.89	90.74	11
B8_G17	NCBI	d__Archaea;p__Thermoproteota;c__Korarchaeia;o__Korarchaeales;f__QMVU01;g__QMVU01;s__QMVU01 sp003661265	72.7	71.4	3.3	3.4	20	46.9	1.5	0	294	294	5645	5645	5071	5071	18914	18914	3	89.99	11
B81_G16	NCBI	d__Archaea;p__Thermoproteota;c__Korarchaeia;o__Korarchaeales;f__Korarchaeaceae;g__;s__	63.2	59.9	0.0	0.3	0	43.8	1.1	0	257	257	4271	4271	4294	4294	17841	17841	3.08	90.2	11
B85_G9	NCBI	d__Archaea;p__Thermoproteota;c__Korarchaeia;o__Korarchaeales;f__Korarchaeaceae;g__DRBY01;s__	64.1	73.5	3.3	3	20	59.4	1.4	0	339	339	4137	4137	4096	4096	22573	22573	10.1	89.99	11
Bin_S22	NCBI	d__Archaea;p__Thermoproteota;c__Korarchaeia;o__Korarchaeales;f__Korarchaeaceae;g__Korarchaeum;s__	92.5	99.5	1.9	1	0	48.6	1.4	0	25	25	147713	147713	55647	55647	389235	389235	1.34	91.18	11
HyVt_138	NCBI	d__Archaea;p__Thermoproteota;c__Korarchaeia;o__Korarchaeales;f__Korarchaeaceae;g__;s__	62.9	68.9	0.9	0.8	0	50.3	0.8	0	198	198	3871	3871	4014	4014	10708	10708	1.96	92.1	11
HyVt_161	NCBI	d__Archaea;p__Thermoproteota;c__Korarchaeia;o__Korarchaeales;f__Korarchaeaceae;g__DRBY01;s__	56.9	46.4	5.8	2.3	15	64.2	0.8	0	329	329	2446	2446	2432	2432	6184	6184	2.37	92.64	11
HyVt_197	NCBI	d__Archaea;p__Thermoproteota;c__Korarchaeia;o__DRAE01;f__DRAE01;g__DRAE01;s__DRAE01 sp011041895	90.6	85.5	1.9	0	50	40.9	0.9	0	155	155	8413	8413	6052	6052	41072	41072	1.38	95.01	11
HyVt_231	NCBI	d__Archaea;p__Thermoproteota;c__Korarchaeia;o__Korarchaeales;f__QMVU01;g__QMVU01;s__	48.7	56.2	2.8	2	50	46	0.6	0	229	229	2808	2808	2734	2734	9732	9732	2.58	90.63	11
HyVt_239	NCBI	d__Archaea;p__Thermoproteota;c__Korarchaeia;o__Korarchaeales;f__Korarchaeaceae;g__DRBY01;s__DRBY01 sp011042755	72.5	69.2	1.9	4.1	67	57.1	1.1	0	148	148	8236	8236	7366	7366	27732	27732	1.96	91.94	11
MDKW	NCBI	d__Archaea;p__Thermoproteota;c__Korarchaeia;o__Korarchaeales;f__Korarchaeaceae;g__Methanodesulfokores;s__Methanodesulfokores washburnensis	93.9	98.3	2.8	5.4	0	42.9	2.9	1745	179	182	24753	24753	16436	16155	147482	147482	2.03	88.5	11
NM4	NCBI	d__Archaea;p__Thermoproteota;c__Korarchaeia;o__Korarchaeales;f__Korarchaeaceae;g__Methanodesulfokores;s__Methanodesulfokores washburnensis	85.5	82.8	1.9	0.8	67	43.4	1.4	383	122	122	13364	13363	11608	11605	49729	49715	1.51	90.79	11
NZ13_K	NCBI	d__Archaea;p__Thermoproteota;c__Korarchaeia;o__Korarchaeales;f__Korarchaeaceae;g__Korarchaeum;s__Korarchaeum sp003344655	88.3	96.5	0.9	0.3	0	56.7	1.4	0	148	148	14152	14152	9285	9285	107729	107729	2.66	90.97	11
OPF8	NCBI	d__Archaea;p__Thermoproteota;c__Korarchaeia;o__Korarchaeales;f__Korarchaeaceae;g__Korarchaeum;s__Korarchaeum cryptofilum	93.4	99.7	2.8	0.9	0	49	1.6	0	1	1	1590757	1590757	2E+06	2E+06	2E+06	1590757	0	91.31	11
S016_48_esom	NCBI	d__Archaea;p__Thermoproteota;c__Korarchaeia;o__Korarchaeales;f__Korarchaeaceae;g__;s__	92.5	92.4	1.0	0.7	50	41.8	1.5	0	63	63	36952	36952	23455	23455	122034	122034	2.07	87.1	11
S143_77_esom	NCBI	d__Archaea;p__Thermoproteota;c__Korarchaeia;o__Korarchaeales;f__Korarchaeaceae;g__;s__	89.7	86.7	4.2	2	17	56.9	1.6	0	233	233	9178	9178	6685	6685	33984	33984	2.46	91.94	11
SpSt_15	NCBI	d__Archaea;p__Thermoproteota;c__Korarchaeia;o__Korarchaeales;f__Korarchaeaceae;g__Korarchaeum;s__Korarchaeum sp011056255	92.4	94.7	2.8	1.3	0	47.9	1.5	596	103	103	25500	25497	14903	14898	133468	133446	1.4	90.32	11
SpSt_52	NCBI	d__Archaea;p__Thermoproteota;c__Korarchaeia;o__Korarchaeales;f__Korarchaeaceae;g__Korarchaeum;s__Korarchaeum sp011056255	33.5	35.6	0.0	0	0	47.5	0.4	13	182	182	2299	2299	2309	2309	6103	6103	1.69	93.06	11
SpSt_718	NCBI	d__Archaea;p__Thermoproteota;c__Korarchaeia;o__Korarchaeales;f__Korarchaeaceae;g__Korarchaeum;s__	67.9	71.1	0.9	0.9	100	47.7	1.1	0	362	362	3094	3094	2982	2982	15481	15481	1.62	91.85	11
SpSt_910	NCBI	d__Archaea;p__Thermoproteota;c__Korarchaeia;o__Korarchaeales;f__Korarchaeaceae;g__Korarchaeum;s__Korarchaeum cryptofilum	91.5	96.4	5.1	3.5	14	49.1	1.6	0	231	231	10949	10949	6883	6883	85597	85597	2.02	92.01	11
SpSt_967	NCBI	d__Archaea;p__Thermoproteota;c__Korarchaeia;o__Korarchaeales;f__Korarchaeaceae;g__Methanodesulfokores;s__Methanodesulfokores washburnensis	72.6	72.4	2.1	0.9	33	43.4	1.0	0	238	238	4676	4676	4061	4061	30763	30763	1.89	91.48	11
UWMA_0234	NCBI	d__Archaea;p__Thermoproteota;c__Korarchaeia;o__Korarchaeales;f__QMVU01;g__QMVU01;s__	74.8	72.2	6.6	3.1	9.1	43.6	1.7	0	373	373	5158	5158	4432	4432	19097	19097	2.37	88.87	11
WS	NCBI	d__Archaea;p__Thermoproteota;c__Korarchaeia;o__Korarchaeales;f__Korarchaeaceae;g__Korarchaeum;s__Korarchaeum cryptofilum	93.4	99.7	4.7	1.7	0	48.7	1.8	0	51	51	78545	78545	34697	34697	267458	267458	2.28	90.44	11
WYZ_LMO9	NCBI	d__Archaea;p__Thermoproteota;c__Korarchaeia;o__Korarchaeales;f__Korarchaeaceae;g__Methanodesulfokores;s__Methanodesulfokores washburnensis	90.2	92.6	0.5	2.2	100	43.2	2.2	592	301	301	9151	9150	7453	7451	49729	49715	2.07	89.82	11
ZAV_18	NCBI	d__Archaea;p__Thermoproteota;c__Korarchaeia;o__Korarchaeales;f__Korarchaeaceae;g__Methanodesulfokores;s__	88.3	79.1	0.9	2.8	0	44	1.3	0	358	358	4481	4481	3492	3492	22865	22865	1.84	91.48	11
12ROV10_HD34A_Bin_00018	This study	d__Archaea;p__Thermoproteota;c__Korarchaeia;o__Korarchaeales;f__Korarchaeaceae;g__;s__	94.3	99	1.9	3.6	0	45.1	1.8	0	58	58	80181	80181	30959	30959	239561	239561	2.98	88.16	11
12ROV10_HD34C_MAG_00048	This study	d__Archaea;p__Thermoproteota;c__Korarchaeia;o__Korarchaeales;f__Korarchaeaceae;g__;s__	84.7	69.5	0.0	0.1	0	43.8	1.0	0	184	184	5564	5564	5236	5236	26874	26874	2.33	91.52	11
12ROV10_HD34E_MAG_00031	This study	d__Archaea;p__Thermoproteota;c__Korarchaeia;o__Korarchaeales;f__Korarchaeaceae;g__;s__	89.7	93.8	1.9	1.6	0	44.5	1.4	0	177	177	11053	11053	8042	8042	51779	51779	2.9	89.54	11
16ROV9_HD21_MAG_00046	This study	d__Archaea;p__Thermoproteota;c__Korarchaeia;o__Korarchaeales;f__Korarchaeaceae;g__;s__	93.4	100	2.2	2.7	33	52.8	2.1	0	74	74	47741	47741	27772	27772	371086	371086	2.99	89.76	11
16ROV9_HD22_Bin_00127	This study	d__Archaea;p__Thermoproteota;c__Korarchaeia;o__Korarchaeales;f__Korarchaeaceae;g__;s__	56.6	42.5	1.9	1.3	0	53.6	0.7	0	466	466	1621	1621	1603	1603	4847	4847	2.71	92.17	11
16ROV9_HD24_Bin_00133	This study	d__Archaea;p__Thermoproteota;c__Korarchaeia;o__Korarchaeales;f__Korarchaeaceae;g__;s__	57.3	73.4	8.5	5.1	0	52.7	1.1	0	639	639	1842	1842	1778	1778	9923	9923	2.39	92.36	11
16ROV9_HD24_MAG_00031	This study	d__Archaea;p__Thermoproteota;c__Korarchaeia;o__Korarchaeales;f__Korarchaeaceae;g__;s__	93.4	100	1.9	1.7	0	53.2	1.6	0	84	84	32228	32228	19613	19613	126781	126781	1.57	90.36	11
17ROV19_HD25_MAG_00082	This study	d__Archaea;p__Thermoproteota;c__Korarchaeia;o__Korarchaeales;f__Korarchaeaceae;g__;s__	92.1	96.1	2.3	3.5	75	53.2	1.5	0	66	66	38932	38932	22972	22972	205234	205234	3.39	91.38	11
17ROV19_HD25_MAG_00106	This study	d__Archaea;p__Thermoproteota;c__Korarchaeia;o__Korarchaeales;f__Korarchaeaceae;g__DRBY01;s__	85.0	88.2	2.9	0.9	17	63.3	1.8	0	343	343	8963	8963	5357	5357	56416	56416	2.99	90.24	11
17ROV19_HD4_Bin_00097	This study	d__Archaea;p__Thermoproteota;c__Korarchaeia;o__Korarchaeales;f__Korarchaeaceae;g__;s__	80.5	63.9	7.9	2.6	0	54.8	1.3	0	630	630	2328	2328	2121	2121	17018	17018	4.93	91.13	11
Aegir_M25_B107	This study	d__Archaea;p__Thermoproteota;c__Korarchaeia;o__Korarchaeales;f__Korarchaeaceae;g__;s__	93.4	99.5	0.9	2.5	0	51.8	1.6	0	43	43	62726	62726	38151	38151	266625	266625	2.2	90.3	11
Aegir_M25_B147	This study	d__Archaea;p__Thermoproteota;c__Korarchaeia;o__Korarchaeales;f__Korarchaeaceae;g__DRBY01;s__	89.7	96.3	2.3	1.1	25	63.3	1.8	0	63	63	61089	61089	28306	28306	128413	128413	1.91	90.35	11

Aegir_M25_B233	This study	d__Archaea;p__Thermoproteota;c__Korarchaeia;o__Korarchaeales;f__Korarchaeaceae;g__;s__	93.4	98.4	1.9	1.9	0	53.2	1.7	0	62	62	48115	48115	27677	27677	158771	158771	1.64	90.21	11
Flange_M5_B13	This study	d__Archaea;p__Thermoproteota;c__Korarchaeia;o__Korarchaeales;f__QMVU01;g__QMVU01;s__	58.1	61.4	4.2	3	0	43.5	1.7	833	357	365	5039	5005	4852	4743	18529	18529	1.81	88.16	11
Flange_M5_B24	This study	d__Archaea;p__Thermoproteota;c__Korarchaeia;o__;f__;g__;s__	60.6	63.9	4.7	5.6	0	37.1	1.3	431	280	285	4788	4788	4688	4604	19457	19457	2.33	86.61	11
Flange_M5_B4	This study	d__Archaea;p__Thermoproteota;c__Korarchaeia;o__;f__;g__;s__	95.0	94.1	1.9	3.6	0	39.6	2.2	4055	223	250	12244	11079	9864	8783	49507	49507	1.36	82.02	11
GS19_ROV16_BS03_Bin_00077	This study	d__Archaea;p__Thermoproteota;c__Korarchaeia;o__;f__;g__;s__	50.9	46.9	0.9	0.9	0	39.6	0.8	0	210	210	3533	3533	3721	3721	11013	11013	1.92	82.63	11
INS_M11_B49	This study	d__Archaea;p__Thermoproteota;c__Korarchaeia;o__;f__;g__;s__	77.0	76.4	1.9	0.5	0	36.5	1.3	726	223	233	6273	6084	5668	5421	32838	32838	1.4	86.01	11
INS_M12_B43	This study	d__Archaea;p__Thermoproteota;c__Korarchaeia;o__Korarchaeales;f__Korarchaeaceae;g__DRBY01;s__	88.7	96.4	1.9	1.4	0	63.4	1.9	1256	17	24	161315	128541	114019	80711	367111	243834	1.66	88.78	11
INS_M12_B44	This study	d__Archaea;p__Thermoproteota;c__Korarchaeia;o__Korarchaeales;f__Korarchaeaceae;g__;s__	94.3	98.5	1.9	4.3	0	45.2	1.9	1725	72	84	43579	39721	26588	22769	213544	213544	2.27	87.79	11
INS_M12_B58	This study	d__Archaea;p__Thermoproteota;c__Korarchaeia;o__Korarchaeales;f__Korarchaeaceae;g__;s__	92.5	98.5	1.9	3.2	0	44.6	1.6	421	21	24	169643	169643	75579	66114	283314	283314	0.98	90.39	11
INS_M13_B5	This study	d__Archaea;p__Thermoproteota;c__Korarchaeia;o__Korarchaeales;f__Korarchaeaceae;g__;s__	93.4	99.7	1.9	3.8	0	44.8	1.8	1350	8	15	474797	239031	228953	122018	518194	518194	0.88	88.14	11
INS_M14_B79	This study	d__Archaea;p__Thermoproteota;c__Korarchaeia;o__Korarchaeales;f__Korarchaeaceae;g__Methanodesulfokores;s__	68.3	60	0.9	0.5	0	49.7	1.0	35	159	160	6870	6870	6223	6184	21570	21570	2.11	88.99	11
INS_M19_B79	This study	d__Archaea;p__Thermoproteota;c__Korarchaeia;o__Korarchaeales;f__Korarchaeaceae;g__;s__	93.4	99.6	2.8	3.2	0	44.7	1.6	0	19	19	186043	186043	86125	86125	240706	240706	1.35	89.3	11
INS_M20_B145	This study	d__Archaea;p__Thermoproteota;c__Korarchaeia;o__Korarchaeales;f__Korarchaeaceae;g__;s__	96.4	93.3	2.8	1.7	0	36.6	1.9	0	42	42	139070	139070	44480	44480	666448	666448	1.32	85.34	11
INS_M20_B155	This study	d__Archaea;p__Thermoproteota;c__Korarchaeia;o__;f__;g__;s__	90.2	90	7.5	2.3	17	39.5	2.0	0	386	386	6021	6021	5120	5120	27800	27800	1.72	82.23	11
INS_M20_B204	This study	d__Archaea;p__Thermoproteota;c__Korarchaeia;o__Korarchaeales;f__Korarchaeaceae;g__;s__	57.4	54.8	2.8	1.4	0	42.9	0.6	0	95	95	7746	7746	6456	6456	25529	25529	1.72	92.8	11
INS_M20_B56	This study	d__Archaea;p__Thermoproteota;c__Korarchaeia;o__Korarchaeales;f__Korarchaeaceae;g__;s__	88.4	95.7	1.9	7.3	0	45.2	1.8	0	162	162	19831	19831	11204	11204	55923	55923	2.39	88.62	11
INS_M20_B69	This study	d__Archaea;p__Thermoproteota;c__Korarchaeia;o__Korarchaeales;f__;g__;s__	84.6	84.9	7.0	5	44	45.9	1.9	0	79	79	38758	38758	24454	24454	121164	121164	2.47	89.63	11
INS_M20_B71	This study	d__Archaea;p__Thermoproteota;c__Korarchaeia;o__Korarchaeales;f__Korarchaeaceae;g__;s__	51.2	47.6	0.9	0.3	0	44.7	0.7	0	207	207	3817	3817	3593	3593	14186	14186	1.53	90.11	11
INS_M21_B147	This study	d__Archaea;p__Thermoproteota;c__Korarchaeia;o__;f__;g__;s__	52.8	31.4	0.0	0.2	0	45.9	0.3	0	13	13	40826	40826	21226	21226	67077	67077	1.24	89.14	11
INS_M21_B149	This study	d__Archaea;p__Thermoproteota;c__Korarchaeia;o__Korarchaeales;f__Korarchaeaceae;g__;s__	87.8	89	0.9	0.8	0	44.4	1.4	0	157	157	12062	12062	8736	8736	42654	42654	1.72	90.47	11
INS_M21_B166	This study	d__Archaea;p__Thermoproteota;c__Korarchaeia;o__Korarchaeales;f__;g__;s__	67.5	66.7	0.9	0.1	0	42.5	0.5	0	19	19	38542	38542	23997	23997	57150	57150	1.12	93.98	11
INS_M21_B183	This study	d__Archaea;p__Thermoproteota;c__Korarchaeia;o__Korarchaeales;f__Korarchaeaceae;g__;s__	87.5	91.2	0.9	0	0	65.5	1.1	0	4	4	687330	687330	279141	279141	687330	687330	1.1	93.58	11
INS_M21_B186	This study	d__Archaea;p__Thermoproteota;c__Korarchaeia;o__Korarchaeales;f__Korarchaeaceae;g__;s__	70.7	63.1	6.5	2.5	0	43.9	0.8	0	33	33	60568	60568	23448	23448	119497	119497	1.05	93.24	11
INS_M21_B208	This study	d__Archaea;p__Thermoproteota;c__Korarchaeia;o__Korarchaeales;f__Korarchaeaceae;g__Methanodesulfokores;s__	92.5	86.1	0.9	1.3	0	49.6	1.3	0	45	45	43098	43098	29599	29599	106628	106628	1.97	88.9	11
INS_M21_B31	This study	d__Archaea;p__Thermoproteota;c__Korarchaeia;o__;f__;g__;s__	77.7	71.4	0.9	1.1	0	41.1	1.4	0	293	293	5363	5363	4644	4644	20307	20307	2.16	87.16	11
INS_M21_B37	This study	d__Archaea;p__Thermoproteota;c__Korarchaeia;o__;f__;g__;s__	93.6	92.1	2.8	1.2	0	36.6	1.8	0	68	68	45825	45825	25780	25780	133320	133320	1.03	86.26	11
INS_M21_B77	This study	d__Archaea;p__Thermoproteota;c__Korarchaeia;o__Korarchaeales;f__Korarchaeaceae;g__;s__	62.5	61.6	3.7	6.4	0	43.4	1.0	0	106	106	17353	17353	9606	9606	50414	50414	1.5	91.25	11
INS_M22_B116	This study	d__Archaea;p__Thermoproteota;c__Korarchaeia;o__Korarchaeales;f__Korarchaeaceae;g__;s__	82.0	89.9	1.9	0.1	0	66.4	1.4	0	188	188	8998	8998	7228	7228	34205	34205	3.9	92.78	11
INS_M22_B125	This study	d__Archaea;p__Thermoproteota;c__Korarchaeia;o__Korarchaeales;f__Korarchaeaceae;g__;s__	91.2	95.5	0.9	7.1	0	45.1	1.8	0	114	114	31409	31409	15673	15673	165006	165006	2.35	89.17	11
INS_M22_B44	This study	d__Archaea;p__Thermoproteota;c__Korarchaeia;o__Korarchaeales;f__Korarchaeaceae;g__;s__	92.5	83.6	1.9	0.2	0	44.4	1.0	0	4	4	416280	416280	238327	238327	474743	474743	0.45	92.09	11
INS_M22_B49	This study	d__Archaea;p__Thermoproteota;c__Korarchaeia;o__;f__;g__;s__	50.2	37.1	2.8	1.4	20	36.7	0.8	0	255	255	3016	3016	3042	3042	8369	8369	1.81	85.57	11
INS_M22_B97	This study	d__Archaea;p__Thermoproteota;c__Korarchaeia;o__Korarchaeales;f__Korarchaeaceae;g__Methanodesulfokores;s__	93.5	86.5	0.9	1.4	0	49.6	1.3	0	45	45	42409	42409	29915	29915	105855	105855	1.64	88.89	11
INS_M22_B98	This study	d__Archaea;p__Thermoproteota;c__Korarchaeia;o__Korarchaeales;f__Korarchaeaceae;g__DRBY01;s__	60.8	66.2	0.9	10	100	61.5	1.2	0	43	43	43869	43869	27445	27445	116452	116452	1.03	91.98	11
INS_M29_B128	This study	d__Archaea;p__Thermoproteota;c__Korarchaeia;o__Korarchaeales;f__QMVU01;g__QMVU01;s__	59.6	54.3	0.0	0.2	0	44	1.2	0	305	305	4273	4273	3994	3994	13143	13143	2.02	87.63	11
INS_M34_B48	This study	d__Archaea;p__Thermoproteota;c__Korarchaeia;o__;f__;g__;s__	95.5	91	2.9	0.9	20	36.6	1.8	0	239	239	9416	9416	7356	7356	35229	35229	1.49	85.38	11
INS_M34_B71	This study	d__Archaea;p__Thermoproteota;c__Korarchaeia;o__Korarchaeales;f__Korarchaeaceae;g__Methanodesulfokores;s__	93.5	87.9	0.9	1	0	49.5	1.4	0	41	41	45985	45985	33094	33094	121065	121065	1.55	88.57	11
INS_M9_B137	This study	d__Archaea;p__Thermoproteota;c__Korarchaeia;o__Korarchaeales;f__Korarchaeaceae;g__DRBY01;s__	77.7	83.7	8.4	4.1	60	62.4	1.8	0	290	290	8334	8334	6225	6225	38203	38203	2.38	91.23	11
INS_M9_B20	This study	d__Archaea;p__Thermoproteota;c__Korarchaeia;o__Korarchaeales;f__Korarchaeaceae;g__;s__	85.9	70.7	0.0	0	0	44.4	0.7	0	3	3	456221	456221	217453	217453	456221	456221	0.3	92.15	11
