# Supplementary material for: Potential for homoacetogenesis via the Wood–Ljungdahl pathway in Korarchaeia lineages from marine hydrothermal vents
Source: Environ Microbiol Rep. 2023 May 22;15(6):698–707. doi: 10.1111/1758-2229.13168 (PMC10667645; doi:10.1111/1758-2229.13168)
Supplement: Supplementary file 11 — Table S2. List of genes used for buiding concatenated phylogenies with 42 and 115 markers. [file EMI4-15-698-s001.pdf]

Supplementary Table 2. List of genes used for buiding concatenated phylogenies with 42 and 115 markers.

42-markers set		
ar122_r202_all_PF00466.21	ar122_r202_all_TIGR00291	ar122_r202_all_TIGR01046
ar122_r202_all_PF00687.22	ar122_r202_all_TIGR00308	ar122_r202_all_TIGR02338
ar122_r202_all_PF00827.18	ar122_r202_all_TIGR00373	ar122_r202_all_TIGR02389
ar122_r202_all_PF00900.21	ar122_r202_all_TIGR00405	ar122_r202_all_TIGR02390
ar122_r202_all_PF01015.19	ar122_r202_all_TIGR00448	ar122_r202_all_TIGR03626
ar122_r202_all_PF01092.20	ar122_r202_all_TIGR00491	ar122_r202_all_TIGR03627
ar122_r202_all_PF01200.19	ar122_r202_all_TIGR00982	ar122_r202_all_TIGR03628
ar122_r202_all_PF01280.21	ar122_r202_all_TIGR01008	ar122_r202_all_TIGR03629
ar122_r202_all_PF01866.18	ar122_r202_all_TIGR01012	ar122_r202_all_TIGR03653
ar122_r202_all_PF07541.13	ar122_r202_all_TIGR01018	ar122_r202_all_TIGR03670
ar122_r202_all_PF13656.7	ar122_r202_all_TIGR01020	ar122_r202_all_TIGR03672
ar122_r202_all_TIGR00037	ar122_r202_all_TIGR01025	ar122_r202_all_TIGR03673
ar122_r202_all_TIGR00264	ar122_r202_all_TIGR01028	ar122_r202_all_TIGR03680
ar122_r202_all_TIGR00279	ar122_r202_all_TIGR01038	ar122_r202_all_TIGR03722

115-markers set		
ar122_r202_all_PF00368.19	ar122_r202_all_TIGR00111	ar122_r202_all_TIGR01018
ar122_r202_all_PF00410.20	ar122_r202_all_TIGR00134	ar122_r202_all_TIGR01020
ar122_r202_all_PF00466.21	ar122_r202_all_TIGR00240	ar122_r202_all_TIGR01025
ar122_r202_all_PF00687.22	ar122_r202_all_TIGR00264	ar122_r202_all_TIGR01028
ar122_r202_all_PF00827.18	ar122_r202_all_TIGR00270	ar122_r202_all_TIGR01038
ar122_r202_all_PF00900.21	ar122_r202_all_TIGR00279	ar122_r202_all_TIGR01046
ar122_r202_all_PF01000.27	ar122_r202_all_TIGR00283	ar122_r202_all_TIGR01052
ar122_r202_all_PF01015.19	ar122_r202_all_TIGR00291	ar122_r202_all_TIGR01060
ar122_r202_all_PF01090.20	ar122_r202_all_TIGR00293	ar122_r202_all_TIGR01077
ar122_r202_all_PF01092.20	ar122_r202_all_TIGR00307	ar122_r202_all_TIGR01080
ar122_r202_all_PF01157.19	ar122_r202_all_TIGR00308	ar122_r202_all_TIGR01213
ar122_r202_all_PF01191.20	ar122_r202_all_TIGR00323	ar122_r202_all_TIGR01309
ar122_r202_all_PF01194.18	ar122_r202_all_TIGR00324	ar122_r202_all_TIGR01952
ar122_r202_all_PF01198.20	ar122_r202_all_TIGR00335	ar122_r202_all_TIGR02076
ar122_r202_all_PF01200.19	ar122_r202_all_TIGR00336	ar122_r202_all_TIGR02153
ar122_r202_all_PF01269.18	ar122_r202_all_TIGR00337	ar122_r202_all_TIGR02236
ar122_r202_all_PF01280.21	ar122_r202_all_TIGR00373	ar122_r202_all_TIGR02258
ar122_r202_all_PF01282.20	ar122_r202_all_TIGR00389	ar122_r202_all_TIGR02338
ar122_r202_all_PF01496.20	ar122_r202_all_TIGR00405	ar122_r202_all_TIGR02389
ar122_r202_all_PF01655.19	ar122_r202_all_TIGR00408	ar122_r202_all_TIGR02390
ar122_r202_all_PF01798.19	ar122_r202_all_TIGR00425	ar122_r202_all_TIGR02651
ar122_r202_all_PF01864.18	ar122_r202_all_TIGR00432	ar122_r202_all_TIGR03626
ar122_r202_all_PF01866.18	ar122_r202_all_TIGR00442	ar122_r202_all_TIGR03627
ar122_r202_all_PF01868.17	ar122_r202_all_TIGR00448	ar122_r202_all_TIGR03628
ar122_r202_all_PF01984.21	ar122_r202_all_TIGR00456	ar122_r202_all_TIGR03629
ar122_r202_all_PF01990.18	ar122_r202_all_TIGR00463	ar122_r202_all_TIGR03636
ar122_r202_all_PF02006.17	ar122_r202_all_TIGR00468	ar122_r202_all_TIGR03653
ar122_r202_all_PF02978.20	ar122_r202_all_TIGR00471	ar122_r202_all_TIGR03665
ar122_r202_all_PF03874.17	ar122_r202_all_TIGR00490	ar122_r202_all_TIGR03670
ar122_r202_all_PF04019.13	ar122_r202_all_TIGR00491	ar122_r202_all_TIGR03671
ar122_r202_all_PF04104.15	ar122_r202_all_TIGR00501	ar122_r202_all_TIGR03672
ar122_r202_all_PF04919.13	ar122_r202_all_TIGR00521	ar122_r202_all_TIGR03673
ar122_r202_all_PF07541.13	ar122_r202_all_TIGR00522	ar122_r202_all_TIGR03674
ar122_r202_all_PF13656.7	ar122_r202_all_TIGR00549	ar122_r202_all_TIGR03677
ar122_r202_all_PF13685.7	ar122_r202_all_TIGR00729	ar122_r202_all_TIGR03680
ar122_r202_all_TIGR00021	ar122_r202_all_TIGR00936	ar122_r202_all_TIGR03684
ar122_r202_all_TIGR00037	ar122_r202_all_TIGR00982	ar122_r202_all_TIGR03722
ar122_r202_all_TIGR00042	ar122_r202_all_TIGR01008	
ar122_r202_all_TIGR00064	ar122_r202_all_TIGR01012	
