# Supplementary material for: Potential for homoacetogenesis via the Wood–Ljungdahl pathway in Korarchaeia lineages from marine hydrothermal vents
Source: Environ Microbiol Rep. 2023 May 22;15(6):698–707. doi: 10.1111/1758-2229.13168 (PMC10667645; doi:10.1111/1758-2229.13168)
Supplement: Supplementary file 12 — Table S3. List of genes accession numbers used to compile the survey presented in Figure 2, through the pipeline presented in Dombrowski et al. (2018). [file EMI4-15-698-s009.pdf]

**Supplementary Table 4.** List of genes accession numbers used to compile the survey presented in Figure 2, through the pipeline presented in Dombrowski et al., 2020.

| pathway | GeneID | Gene | Gene_long |
|---|---|---|---|
| Amino Acids degradation | K00812 | aspB | aspB; aspartate aminotransferase [EC:2.6.1.1] |
| Amino Acids degradation | K01915 | glnA | glutamine synthetase [EC:6.3.1.2] |
| Amino Acids degradation | K00260 | gudB | gudB, rocG; glutamate dehydrogenase [EC:1.4.1.2] |
| Amino Acids degradation | K01940 | argG | argG, ASS1; argininosuccinate synthase [EC:6.3.4.5] |
| Amino Acids degradation | K01755 | argH | argH, ASL; argininosuccinate lyase [EC:4.3.2.1] |
| Amino Acids degradation | K01939 | purA | purA, ADSS; adenylosuccinate synthase [EC:6.3.4.4] |
| Amino Acids degradation | K01756 | purB | purB, ADSL; adenylosuccinate lyase [EC:4.3.2.2] |
| Amino Acids degradation | K00282 | gcvPA | gcvPA; glycine dehydrogenase subunit 1 [EC:1.4.4.2] |
| Amino Acids degradation | K00605 | gcvT | gcvT, AMT; aminomethyltransferase [EC:2.1.2.10] |
| Amino Acids degradation | K00600 | glyA | glyA, SHMT; glycine hydroxymethyltransferase [EC:2.1.2.1] |
| Amino Acids degradation | K01754 | ilvA | E4.3.1.19, ilvA, tdcB; threonine dehydratase [EC:4.3.1.19] |
| Amino Acids degradation | K01473 | hyuA | hyuA; N-methylhydantoinase A [EC:3.5.2.14] |
| Amino Acids degradation | K01474 | hyuB | hyuB; N-methylhydantoinase B [EC:3.5.2.14] |
| Amino Acids degradation | K00302 | soxA | soxA; sarcosine oxidase, subunit alpha [EC:1.5.3.1] |
| Amino Acids degradation | K00303 | soxB | soxB; sarcosine oxidase, subunit beta [EC:1.5.3.1] |
| Amino Acids degradation | K15583 | oppD | oppD; oligopeptide transport system ATP-binding protein |
| Amino Acids degradation | K10036 | glnH | glnH; glutamine transport system substrate-binding protein |
| Amino Acids degradation | K10037 | glnP | glnP; glutamine transport system permease protein |
| Pentose-derived sugar metabolism | K18237 | K18237 | K18237; ribose 1,5-bisphosphate isomerase [EC:5.3.1.29] |
| Pentose-derived sugar metabolism | K18931 | ampp | ampp; AMP phosphorylase [EC:2.4.2.57] |
| Pentose-derived sugar metabolism | K01601 | rbcL | rbcL; ribulose-bisphosphate carboxylase large chain [EC:4.1.1.39] |
| Pentose-derived sugar metabolism | K01619 | deoC | deoC, DERA; deoxyribose-phosphate aldolase [EC:4.1.2.4] |
| Pentose-derived sugar metabolism | K00852 | rbsK | rbsK, RBKS; ribokinase [EC:2.7.1.15] |
| Pentose-derived sugar metabolism | K00948 | PRPS | PRPS, prsA; ribose-phosphate pyrophosphokinase [EC:2.7.6.1] |
| Pentose-derived sugar metabolism | K00134 | GAPDH | GAPDH, gapA; glyceraldehyde 3-phosphate dehydrogenase [EC:1.2.1.12] |
| Pentose-derived sugar metabolism | K00150 | gap2 | gap2; glyceraldehyde-3-phosphate dehydrogenase (NAD(P)) [EC:1.2.1.59] |
| Pentose-derived sugar metabolism | K00927 | PGK | PGK, pgk; phosphoglycerate kinase [EC:2.7.2.3] |
| Pentose-derived sugar metabolism | K15635 | apgM | 2,3-bisphosphoglycerate-independent phosphoglycerate mutase [EC:5.4.2.12] |
| Pentose-derived sugar metabolism | K01689 | ENO | ENO, eno; enolase [EC:4.2.1.11] |
| TCA and Pyruvate metabolism | K00239 | sdhA | sdhA, frdA; succinate dehydrogenase / fumarate reductase, flavoprotein subunit [EC:1.3.5.1 1.3.5.4] |
| TCA and Pyruvate metabolism | K01902 | sucD | sucD; succinyl-CoA synthetase alpha subunit [EC:6.2.1.5] |
| TCA and Pyruvate metabolism | K01903 | sucC | sucC; succinyl-CoA synthetase beta subunit [EC:6.2.1.5] |
| TCA and Pyruvate metabolism | K01677 | fumA | E4.2.1.2AA, fumA; fumarate hydratase subunit alpha [EC:4.2.1.2] |
| TCA and Pyruvate metabolism | K01678 | fumB | E4.2.1.2AB, fumB; fumarate hydratase subunit beta [EC:4.2.1.2] |
| TCA and Pyruvate metabolism | K00027 | ME2 | ME2, sfcA, maeA; malate dehydrogenase (oxaloacetate-decarboxylating) [EC:1.1.1.38] |
| TCA and Pyruvate metabolism | K01006 | ppdk | ppdK; pyruvate, orthophosphate dikinase [EC:2.7.9.1] |

| | | | |
|---|---|---|---|
| TCA and Pyruvate metabolism | K01007 | pps | pps, ppsA; pyruvate, water dikinase [EC:2.7.9.2] |
| TCA and Pyruvate metabolism | K01610 | pckA | E4.1.1.49, pckA; phosphoenolpyruvate carboxykinase (ATP) [EC:4.1.1.49] |
| TCA and Pyruvate metabolism | K01905 | acdA | E6.2.1.13; acetyl-CoA synthetase (ADP-forming) [EC:6.2.1.13] |
| Ferredoxin oxidoreductase | K03738 | AOR | aor; aldehyde:ferredoxin oxidoreductase [EC:1.2.7.5] |
| Ferredoxin oxidoreductase | K00174 | KOR | korA, oorA, oforA; 2-oxoglutarate/2-oxoacid ferredoxin oxidoreductase subunit alpha [EC:1.2.7.3 1.2.7.11] |
| Ferredoxin oxidoreductase | K00175 | KOR | korB, oorB, oforB; 2-oxoglutarate/2-oxoacid ferredoxin oxidoreductase subunit beta [EC:1.2.7.3 1.2.7.11] |
| Ferredoxin oxidoreductase | K00176 | KOR | korD, oorD; 2-oxoglutarate ferredoxin oxidoreductase subunit delta [EC:1.2.7.3] |
| Ferredoxin oxidoreductase | K00177 | KOR | korC, oorC; 2-oxoglutarate ferredoxin oxidoreductase subunit gamma [EC:1.2.7.3] |
| Ferredoxin oxidoreductase | K00169 | POR | porA; pyruvate ferredoxin oxidoreductase alpha subunit [EC:1.2.7.1] |
| Ferredoxin oxidoreductase | K00170 | POR | porB; pyruvate ferredoxin oxidoreductase beta subunit [EC:1.2.7.1] |
| Ferredoxin oxidoreductase | K00171 | POR | porD; pyruvate ferredoxin oxidoreductase delta subunit [EC:1.2.7.1] |
| Ferredoxin oxidoreductase | K00172 | POR | porG; pyruvate ferredoxin oxidoreductase gamma subunit [EC:1.2.7.1] |
| Methanogenesis | arCOG04857 | McrA | Methyl-coenzyme M reductase I subunit alpha |
| Methanogenesis | TIGR03256 | McrA | Methyl-coenzyme M reductase I subunit alpha |
| Methanogenesis | arCOG04860 | McrB | Methyl-coenzyme M reductase I subunit beta |
| Methanogenesis | TIGR03257 | McrB | Methyl-coenzyme M reductase I subunit beta |
| Methanogenesis | arCOG04858 | McrG | Methyl-coenzyme M reductase I subunit gamma |
| Methanogenesis | TIGR03259 | McrG | Methyl-coenzyme M reductase I subunit gamma |
| Methanogenesis | K00577 | mtrA | Methyl-H4M(S)PT:HS-CoM methyltransferase(Mtr) subunitA |
| Methanogenesis | K00578 | mtrB | tetrahydromethanopterin S-methyltransferase subunit B |
| Methanogenesis | K00579 | mtrC | tetrahydromethanopterin S-methyltransferase subunit C |
| Methanogenesis | K00580 | mtrD | tetrahydromethanopterin S-methyltransferase subunit D |
| Methanogenesis | K00581 | mtrE | tetrahydromethanopterin S-methyltransferase subunit E |
| Methanogenesis | K00582 | mtrF | tetrahydromethanopterin S-methyltransferase subunit F |
| Methanogenesis | K00583 | mtrG | tetrahydromethanopterin S-methyltransferase subunit G |
| Methanogenesis | K00584 | mtrH | tetrahydromethanopterin S-methyltransferase subunit H |
| Beta-oxidation | K00248 | ACADS | ACADS, bcd; butyryl-CoA dehydrogenase [EC:1.3.8.1], also acyl-CoA-DH |
| Beta-oxidation | K01715 | crt | crt; enoyl-CoA hydratase [EC:4.2.1.17] |
| Beta-oxidation | K15016 | K15016 | K15016; enoyl-CoA hydratase / 3-hydroxyacyl-CoA dehydrogenase [EC:4.2.1.17 1.1.1.35] |
| Beta-oxidation | K00626 | atoB | E2.3.1.9, atoB; acetyl-CoA C-acetyltransferase [EC:2.3.1.9] |
| Methyltransferases | TIGR01114 | MtoA | N5-methyltetrahydromethanopterin:coenzyme_M_methyltransferase_subunit_H |
| Methyltransferases | PF02007 | MtoA | N5-methyltetrahydromethanopterin:coenzyme_M_methyltransferase_subunit_H |
| Methyltransferases | arCOG04336 | MtoA | N5-methyltetrahydromethanopterin:coenzyme_M_methyltransferase_subunit_H |
| Methyltransferases | TIGR02370 | MtoC | methyltransferase_cognate_corrinoid_proteins,_Methanosarcina_family |
| Methyltransferases | PF02310 | MtoC | methyltransferase_cognate_corrinoid_proteins,_Methanosarcina_family |
| Methyltransferases | arCOG02028 | MtoC | methyltransferase_cognate_corrinoid_proteins,_Methanosarcina_family |
| Methyltransferases | TIGR04270 | MtoD | methylamine_methyltransferase_corrinoid_protein_reductive_activase |
| Methyltransferases | PF14574 | MtoD | methylamine_methyltransferase_corrinoid_protein_reductive_activase |
| Methyltransferases | arCOG02035 | MtoD | methylamine_methyltransferase_corrinoid_protein_reductive_activase |

| | | | |
|---|---|---|---|
| Methyltransferases | arCOG03323 | MtbA | Methylcobalamin:coenzyme M methyltransferase |
| Methyltransferases | K14080 | MtbA | Methylcobalamin:coenzyme M methyltransferase |
| Methyltransferases | arCOG02028 | MtbC1 | Methanogenic corrinoid protein MtbC1 |
| Methyltransferases | K14084 | MtbC1 | Methanogenic corrinoid protein MtbC1 |
| Methyltransferases | arCOG05143 | MtmB | Monomethylamine methyltransferase |
| Methyltransferases | K16176 | MtmB | Monomethylamine methyltransferase |
| Methyltransferases | arCOG03405 | MttB1 | Trimethylamine:corrinoid methyltransferase |
| Methyltransferases | K14083 | MttB1 | Trimethylamine:corrinoid methyltransferase |
| CODH | arCOG02428 | CdhA | Acetyl-CoA decarbonylase/synthase complex subunit alpha 1 |
| CODH | arCOG04408 | CdhB | Acetyl-CoA decarbonylase/synthase complex subunit epsilon 1 |
| CODH | TIGR00316 | CdhC | Acetyl-CoA decarbonylase/synthase complex subunit beta 2 |
| CODH | arCOG04360 | CdhC | Acetyl-CoA decarbonylase/synthase complex subunit beta |
| CODH | arCOG01980 | CdhD | Acetyl-CoA decarbonylase/synthase complex subunit delta |
| CODH | K00197 | cdhE | acetyl-CoA decarbonylase/synthase complex subunit gamma |
| WLP | K00320 | mer | Methylene-H4M(S)PT reductase(Mer) |
| WLP | K00319 | mtd | Methylene-H4M(S)PT dehydrogenase(Hmd) |
| WLP | K01499 | mch | Methenye-H4M(S)PT cyclohydrolase(Mch) |
| WLP | K00672 | ftr | Formyl-MFR:H4M(S)PT formyltransferase(Ftr) |
| WLP | K00200 | fwdA | Formyl-MFR dehydrogenase(Fmd) subunit A |
| WLP | K00201 | fwdB | Formyl-MFR dehydrogenase(Fmd) subunit B |
| WLP | K00202 | fwdC | Formyl-MFR dehydrogenase(Fmd) subunit C |
| WLP | K00203 | fwdD | Formyl-MFR dehydrogenase(Fmd) subunit D |
| WLP | K11261 | fwdE | formylmethanofuran dehydrogenase subunit E |
| WLP | K00205 | fwdF | 4Fe-4S ferredoxin |
| WLP | K11260 | fwdG | 4Fe-4S ferredoxin |
| WLP | K00204 | fwdH | 4Fe-4S ferredoxin |
| Energy conservation | K03388 | hdrA2 | heterodisulfide reductase subunit A2 |
| Energy conservation | K03389 | hdrB2 | heterodisulfide reductase subunit B2 |
| Energy conservation | K03390 | hdrC2 | heterodisulfide reductase subunit C2 |
| Energy conservation | K08264 | hdrD | heterodisulfide reductase subunit D |
| Energy conservation | K14127 | MvhD | F420-non-reducing hydrogenase subunit D |
| Energy conservation | K14126 | MvhA | F420-non-reducing hydrogenase subunit A |
| Energy conservation | K14128 | MvhG | F420-non-reducing hydrogenase subunit G |
| Energy conservation | arCOG01557 | NuoA/fpoB | NADH dehydrogenase, subunit A (Ubiquinone)/fpoB |
| Energy conservation | arCOG01554 | NuoB/fpoA | NADH dehydrogenase, subunit B (Ubiquinone)/fpoA |
| Energy conservation | arCOG01551 | NuoC/fpoC | NADH dehydrogenase, subunit B (Ubiquinone)/fpoC |
| Energy conservation | arCOG01548 | NuoC/D | NADH-quinone oxidoreductase subunit C/D |
| Energy conservation | K22161 | NuoD/fpoD | NADH dehydrogenase, subunit D (Ubiquinone)/fpoD |
| Energy conservation | arCOG01546 | NuoH/fpoH | F(420)H(2) dehydrogenase subunit H/fpoH |

| | | | |
|---|---|---|---|
| Energy conservation | arCOG01543 | NuoI/fpoI | F(420)H(2) dehydrogenase subunit I/fpoI |
| Energy conservation | arCOG04654 | NuoJ/fpoJ | F(420)H(2) dehydrogenase subunit J/fpoJ |
| Energy conservation | arCOG03073 | NuoK/fpoK | F(420)H(2) dehydrogenase subunit K/fpoK |
| Energy conservation | arCOG01539 | NuoL/fpoL | F(420)H(2) dehydrogenase subunit L/fpoL |
| Energy conservation | arCOG01538 | NuoM/fpoM | F(420)H(2) dehydrogenase subunit M/fpoM |
| Energy conservation | arCOG01540 | NuoN/fpoN | F(420)H(2) dehydrogenase subunit N/fpoN |
| Energy conservation | K22171 | fqoA | fqoA; F420H2:quinone oxidoreductase subunit A [EC:1.1.98.4] |
| Energy conservation | K22172 | fqoBC | fqoBC; F420H2:quinone oxidoreductase subunit B/C [EC:1.1.98.4] |
| Energy conservation | K22173 | fqoD | fqoD; F420H2:quinone oxidoreductase subunit D [EC:1.1.98.4] |
| Energy conservation | K22175 | fqoH | fqoH; F420H2:quinone oxidoreductase subunit H [EC:1.1.98.4] |
| Energy conservation | K22176 | fqoI | fqoI; F420H2:quinone oxidoreductase subunit I [EC:1.1.98.4] |
| Energy conservation | K22174 | fqoF | fqoF; F420H2:quinone oxidoreductase subunit F |
| Energy conservation | K00125 | FrhB | frhB; coenzyme F420 hydrogenase subunit beta [EC:1.12.98.1] |
| Energy conservation | arCOG00868 | AtpA | V-type ATP synthase alpha chain |
| Energy conservation | arCOG00865 | AtpB | V-type ATP synthase beta chain |
| Energy conservation | arCOG02459 | atpC | V-type ATP synthase subunit C |
| Energy conservation | arCOG04101 | AtpD | V-type ATP synthase subunit D |
| Energy conservation | arCOG00869 | AtpE | V-type ATP synthase subunit E |
| Energy conservation | arCOG04102 | AtpF | V-type ATP synthase subunit F |
| Energy conservation | arCOG03363 | AtpH | A-type ATP synthase subunit H |
| Energy conservation | arCOG04138 | AtpI | V-type ATP synthase subunit I |
| Energy conservation | arCOG02455 | AtpK | ATP synthase subunit K (AtpK) |
