# Supplementary material for: Potential for homoacetogenesis via the Wood–Ljungdahl pathway in Korarchaeia lineages from marine hydrothermal vents
Source: Environ Microbiol Rep. 2023 May 22;15(6):698–707. doi: 10.1111/1758-2229.13168 (PMC10667645; doi:10.1111/1758-2229.13168)
Supplement: Supplementary file 13 — Table S4. List of genes of the WLP identified in the genomes of deep‐branching Korarchaeia. Top hits in the NCBI_COG, arCOG, KO, PFAM, TIGR databases and related e‐values are given for all translated sequences. NCBI taxonomic affiliation is also indicated. [file EMI4-15-698-s007.pdf]

Supplementary Table 4. List of genes of the WLP identified in the genomes of deep-branching Korarchaeia. Top hits in the NCBI_COG, arCOG, KO, PFAM, TIGR databases and related e-values are given for all translated sequences. NCBI taxonomic affiliation is also indicated.

	BinID	ProteinLength	NCBI_COG	NCBI_COG_evalu	arcogs	arcogs_geneID	arcogs_evalue	KO_hmm	e_value	bit_score	bit_score_cutoff	PFAM_hmm	Pfam_Evalue	TIGR	EC	TIGR_Evalue	TaxID	TaxString
	INS_M21_B166	246	COG1152	2.60E-15	arCOG02428	CdhA	2.80E-20	K00192	1.40E-11	46.4	377.2	PF13534	1.10E-07	TIGR01945	-	7.70E-11	-	-
	INS_M29_B128	239	COG1152	3.50E-17	arCOG02428	CdhA	1.80E-22	K00192	1.40E-11	46.4	377.2	PF13237	3.40E-07	TIGR01945	-	9.30E-10	-	-
	3300014913_21	777	COG1152	6.80E-136	arCOG02428	CdhA	0	K00192	0	1259	377.2	PF03063	4.00E-106	TIGR00314	1.2.99.2	0	2056630	Candidatus_Korarchaeota_archaeon
	3300024423_7	239	COG4656	5.90E-16	arCOG02428	CdhA	4.00E-22	K03390	2.70E-12	50.3	116.5	PF13187	6.50E-08	TIGR01945	-	1.90E-10	-	-
	3300024423_7	780	COG1152	2.50E-137	arCOG02428	CdhA	0	K00192	0	1253	377.2	PF03063	8.00E-105	TIGR00314	1.2.99.2	0	2056630	Candidatus_Korarchaeota_archaeon
	B14_G2	781	COG1152	4.40E-132	arCOG02428	CdhA	0	K00192	0	1230	377.2	PF03063	2.20E-99	TIGR00314	1.2.99.2	0	2056630	Candidatus_Korarchaeota_archaeon
	B15_G15	775	COG1152	5.40E-138	arCOG02428	CdhA	0	K00192	0	1254	377.2	PF03063	1.10E-104	TIGR00314	1.2.99.2	0	2056630	Candidatus_Korarchaeota_archaeon
	B15_G15	239	COG1152	1.40E-15	arCOG02428	CdhA	4.10E-22	K00192	3.10E-11	45.2	377.2	PF13237	2.00E-07	TIGR01945	-	8.80E-10	2056630	Candidatus_Korarchaeota_archaeon
	B41_G2	780	COG1152	1.90E-137	arCOG02428	CdhA	0	K00192	0	1256	377.2	PF03063	2.90E-106	TIGR00314	1.2.99.2	0	2056630	Candidatus_Korarchaeota_archaeon
	B41_G2	239	COG1152	1.10E-15	arCOG02428	CdhA	8.60E-22	K00192	3.10E-11	45.2	377.2	PF13237	2.00E-07	TIGR01945	-	8.80E-10	2056630	Candidatus_Korarchaeota_archaeon
	B51_G1	239	COG1152	1.10E-15	arCOG02428	CdhA	8.60E-22	K00192	3.10E-11	45.2	377.2	PF13237	2.00E-07	TIGR01945	-	8.80E-10	2056630	Candidatus_Korarchaeota_archaeon
	B51_G1	780	COG1152	1.90E-137	arCOG02428	CdhA	0	K00192	0	1255	377.2	PF03063	1.50E-105	TIGR00314	1.2.99.2	0	2056630	Candidatus_Korarchaeota_archaeon
	B8_G17	775	COG1152	6.30E-137	arCOG02428	CdhA	0	K00192	0	1261	377.2	PF03063	4.90E-108	TIGR00314	1.2.99.2	0	2056630	Candidatus_Korarchaeota_archaeon
	Flange_M5_B13	780	COG1152	3.30E-137	arCOG02428	CdhA	0	K00192	0	1263	377.2	PF03063	3.10E-108	TIGR00314	1.2.99.2	0	-	-
	Flange_M5_B13	239	COG1152	8.40E-16	arCOG02428	CdhA	3.50E-22	K00192	2.70E-11	45.4	377.2	PF13237	1.40E-07	TIGR01945	-	6.00E-10	2056630	Candidatus_Korarchaeota_archaeon
	Flange_M5_B24	240	COG4656	8.60E-17	arCOG02428	CdhA	7.90E-19	K03615	1.90E-14	56.4	127.9	PF13534	3.00E-07	TIGR01945	-	4.40E-12	1906666	Thermoplasmata_archaeon
	Flange_M5_B4	244	COG1152	4.50E-14	arCOG02428	CdhA	2.20E-19	K00192	1.10E-12	50	377.2	PF13534	2.50E-07	TIGR00314	1.2.99.2	1.60E-10	-	-
cdhA	Flange_M5_B4	781	COG1152	1.70E-131	arCOG02428	CdhA	0	K00192	0	1252	377.2	PF03063	5.30E-101	TIGR00314	1.2.99.2	0	1779368	Candidatus_Bathyarchaeota_archaeon_B24
	INS_M11_B49	129	COG1152	0.00099	arCOG02428	CdhA	2.00E-48	K00192	3.10E-51	177.5	377.2	-	-	TIGR00314	1.2.99.2	4.30E-47	-	-
	INS_M11_B49	206	COG4656	2.30E-17	arCOG02428	CdhA	5.30E-19	K03615	1.20E-14	57.1	127.9	PF13187	1.80E-07	TIGR01945	-	3.40E-12	1906666	Thermoplasmata_archaeon
	INS_M20_B145	781	COG1152	9.80E-127	arCOG02428	CdhA	0	K00192	0	1243	377.2	PF03063	6.20E-101	TIGR00314	1.2.99.2	0	2026714	Candidatus_Bathyarchaeota_archaeon
	INS_M20_B145	240	COG4656	8.60E-17	arCOG02428	CdhA	7.90E-19	K03615	1.90E-14	56.4	127.9	PF13534	3.00E-07	TIGR01945	-	4.40E-12	1906666	Thermoplasmata_archaeon
	INS_M20_B155	153	COG1152	5.40E-15	arCOG02428	CdhA	2.40E-19	K00192	5.80E-13	50.9	377.2	PF13187	1.60E-08	TIGR00314	1.2.99.2	4.70E-11	-	-
	INS_M20_B155	646	COG1152	1.90E-119	arCOG02428	CdhA	3.10E-286	K00192	0	1040	377.2	PF03063	2.40E-97	TIGR00314	1.2.99.2	0	1779368	Candidatus_Bathyarchaeota_archaeon_B24
	INS_M20_B69	246	COG1152	2.60E-15	arCOG02428	CdhA	2.80E-20	K00192	1.40E-11	46.4	377.2	PF13534	1.10E-07	TIGR01945	-	7.70E-11	-	-
	INS_M20_B69	777	COG1152	5.50E-133	arCOG02428	CdhA	0	K00192	0	1245	377.2	PF03063	9.40E-105	TIGR00314	1.2.99.2	0	2056630	Candidatus_Korarchaeota_archaeon
	INS_M21_B31	246	COG1152	2.40E-15	arCOG02428	CdhA	1.50E-20	K00192	2.50E-13	52.2	377.2	PF13237	3.60E-07	TIGR00314	1.2.99.2	1.40E-11	2056630	Candidatus_Korarchaeota_archaeon
	INS_M21_B37	688	COG1152	9.70E-127	arCOG02428	CdhA	1.20E-302	K00192	0	1102	377.2	PF03063	1.30E-89	TIGR00314	1.2.99.2	0	2026714	Candidatus_Bathyarchaeota_archaeon
	INS_M21_B37	240	COG4656	3.30E-17	arCOG02428	CdhA	7.10E-19	K03615	1.00E-14	57.3	127.9	PF13534	3.10E-07	TIGR01945	-	4.50E-12	1906666	Thermoplasmata_archaeon
	INS_M22_B49	240	COG4656	5.80E-17	arCOG02428	CdhA	7.20E-19	K03615	1.60E-14	56.6	127.9	PF13534	3.10E-07	TIGR01945	-	4.50E-12	1906666	Thermoplasmata_archaeon
	INS_M34_B48	781	COG1152	9.80E-127	arCOG02428	CdhA	0	K00192	0	1243	377.2	PF03063	6.20E-101	TIGR00314	1.2.99.2	0	2026714	Candidatus_Bathyarchaeota_archaeon
	INS_M34_B48	240	COG4656	5.80E-17	arCOG02428	CdhA	7.20E-19	K03615	1.60E-14	56.6	127.9	PF13534	3.10E-07	TIGR01945	-	4.50E-12	1906666	Thermoplasmata_archaeon
	UWMA_0234	775	COG1152	5.80E-138	arCOG02428	CdhA	0	K00192	0	1261	377.2	PF03063	1.10E-106	TIGR00314	1.2.99.2	0	-	-
	UWMA_0234	239	COG1152	3.00E-16	arCOG02428	CdhA	1.60E-22	K00192	1.20E-11	46.6	377.2	PF13187	5.00E-07	TIGR01945	-	9.50E-10	-	-
	3300014913_21	175	COG1880	9.20E-28	arCOG04408	CdhB	8.80E-39	K00195	7.10E-44	152.9	104.9	PF02552	6.80E-21	TIGR00315	1.2.99.2	9.30E-30	2056630	Candidatus_Korarchaeota_archaeon
	3300024423_7	179	COG1880	1.40E-30	arCOG04408	CdhB	1.10E-45	K00195	1.60E-50	174.6	104.9	PF02552	2.60E-29	TIGR00315	1.2.99.2	1.50E-35	2056630	Candidatus_Korarchaeota_archaeon
cdhB	B14_G2	177	COG1880	4.10E-34	arCOG04408	CdhB	3.50E-46	K00195	3.80E-51	176.6	104.9	PF02552	2.20E-23	TIGR00315	1.2.99.2	2.40E-35	2056630	Candidatus_Korarchaeota_archaeon
	B15_G15	179	COG1880	1.80E-30	arCOG04408	CdhB	1.20E-45	K00195	6.80E-51	175.7	104.9	PF02552	8.80E-30	TIGR00315	1.2.99.2	1.00E-35	2056630	Candidatus_Korarchaeota_archaeon
	B41_G2	179	COG1880	1.50E-31	arCOG04408	CdhB	1.10E-45	K00195	2.30E-51	177.3	104.9	PF02552	1.20E-30	TIGR00315	1.2.99.2	2.50E-36	2056630	Candidatus_Korarchaeota_archaeon
	B51_G1	179	COG1880	1.10E-31	arCOG04408	CdhB	7.00E-46	K00195	1.70E-51	177.7	104.9	PF02552	7.00E-31	TIGR00315	1.2.99.2	1.80E-36	2056630	Candidatus_Korarchaeota_archaeon

	B8_G17	178	COG1880	1.50E-30	arCOG04408	CdhB	1.60E-44	K00195	1.10E-49	171.8	104.9	PF02552	2.30E-28	TIGR00315	1.2.99.2	1.40E-34	2056630	Candidatus_Korarchaeota_archaeon
	Flange_M5_B13	178	COG1880	8.10E-32	arCOG04408	CdhB	4.90E-48	K00195	5.10E-53	182.6	104.9	PF02552	1.20E-29	TIGR00315	1.2.99.2	8.00E-39	-	-
	Flange_M5_B4	179	COG1880	2.60E-39	arCOG04408	CdhB	9.50E-56	K00195	3.70E-63	215.7	104.9	PF02552	3.00E-32	TIGR00315	1.2.99.2	1.80E-45	2012512	Candidatus_Bathyarchaeota_archaeon_ex4484_231
	INS_M11_B49	173	COG1880	1.50E-35	arCOG04408	CdhB	1.90E-50	K00195	5.20E-54	185.9	104.9	PF02552	1.60E-29	TIGR00315	1.2.99.2	4.80E-40	-	-
	INS_M20_B145	173	COG1880	1.50E-35	arCOG04408	CdhB	1.30E-50	K00195	4.70E-54	186	104.9	PF02552	1.60E-29	TIGR00315	1.2.99.2	3.40E-40	-	-
	INS_M20_B69	175	COG1880	4.80E-28	arCOG04408	CdhB	6.20E-39	K00195	6.00E-44	153.1	104.9	PF02552	3.40E-21	TIGR00315	1.2.99.2	2.60E-30	2056630	Candidatus_Korarchaeota_archaeon
	INS_M21_B37	173	COG1880	1.50E-35	arCOG04408	CdhB	1.90E-50	K00195	5.20E-54	185.9	104.9	PF02552	1.60E-29	TIGR00315	1.2.99.2	4.80E-40	-	-
	INS_M34_B48	173	COG1880	1.50E-35	arCOG04408	CdhB	1.30E-50	K00195	4.70E-54	186	104.9	PF02552	1.60E-29	TIGR00315	1.2.99.2	3.40E-40	-	-
	UWMA_0234	177	COG1880	1.10E-32	arCOG04408	CdhB	2.10E-47	K00195	5.20E-52	179.4	104.9	PF02552	3.40E-29	TIGR00315	1.2.99.2	2.20E-38	-	-

	INS_M29_B128	462	COG1614	4.60E-195	arCOG04360	CdhC	6.30E-196	K00193	3.50E-205	685.2	592.2	PF03598	1.20E-65	TIGR00316	1.2.99.2 2.3	5.20E-196	2056630	Candidatus_Korarchaeota_archaeon
	3300014913_21	462	COG1614	5.20E-190	arCOG04360	CdhC	1.80E-192	K00193	5.40E-202	674.7	592.2	PF03598	5.70E-62	TIGR00316	1.2.99.2 2.3	2.40E-190	-	-
	3300024423_7	463	COG1614	2.20E-194	arCOG04360	CdhC	5.60E-196	K00193	1.20E-205	686.8	592.2	PF03598	5.30E-65	TIGR00316	1.2.99.2 2.3	7.90E-197	2056630	Candidatus_Korarchaeota_archaeon
	B10_G17	234	COG1614	2.10E-67	arCOG04360	CdhC	5.10E-74	K00193	4.20E-77	263	592.2	-	-	TIGR00316	1.2.99.2 2.3	9.70E-79	2056630	Candidatus_Korarchaeota_archaeon
	B14_G2	492	COG1614	6.50E-190	arCOG04360	CdhC	8.10E-193	K00193	4.30E-199	665.1	592.2	PF03598	1.20E-60	TIGR00316	1.2.99.2 2.3	4.40E-195	2056630	Candidatus_Korarchaeota_archaeon
	B15_G15	463	COG1614	1.30E-194	arCOG04360	CdhC	3.70E-196	K00193	1.00E-205	687	592.2	PF03598	2.10E-65	TIGR00316	1.2.99.2 2.3	2.20E-196	2056630	Candidatus_Korarchaeota_archaeon
	B41_G2	463	COG1614	2.10E-195	arCOG04360	CdhC	1.10E-196	K00193	8.00E-206	687.3	592.2	PF03598	3.20E-66	TIGR00316	1.2.99.2 2.3	4.70E-196	2056630	Candidatus_Korarchaeota_archaeon
	B51_G1	79	-	-	arCOG04360	CdhC	5.60E-08	K00193	9.90E-08	34.4	592.2	-	-	TIGR00316	1.2.99.2 2.3	0.00022	2056630	Candidatus_Korarchaeota_archaeon
cdhC	Flange_M5_B4	484	COG1614	4.50E-192	arCOG04360	CdhC	1.00E-192	K00193	3.40E-202	675.3	592.2	PF03598	4.00E-63	TIGR00316	1.2.99.2 2.3	2.20E-196	1779368	Candidatus_Bathyarchaeota_archaeon_B24
	GS19_ROV16_BS03_Bin_00077	484	COG1614	1.50E-192	arCOG04360	CdhC	1.20E-193	K00193	8.90E-203	677.2	592.2	PF03598	2.80E-63	TIGR00316	1.2.99.2 2.3	3.80E-197	1779368	Candidatus_Bathyarchaeota_archaeon_B24
	INS_M11_B49	471	COG1614	3.00E-194	arCOG04360	CdhC	1.10E-195	K00193	4.20E-207	691.5	592.2	PF03598	1.80E-65	TIGR00316	1.2.99.2 2.3	1.70E-204	2250277	Thermoprotei_archaeon
	INS_M20_B145	471	COG1614	1.80E-193	arCOG04360	CdhC	3.90E-195	K00193	1.90E-206	689.3	592.2	PF03598	3.90E-65	TIGR00316	1.2.99.2 2.3	6.70E-204	2250277	Thermoprotei_archaeon
	INS_M20_B155	484	COG1614	1.60E-192	arCOG04360	CdhC	1.40E-194	K00193	3.70E-203	678.5	592.2	PF03598	3.60E-63	TIGR00316	1.2.99.2 2.3	3.60E-198	1779368	Candidatus_Bathyarchaeota_archaeon_B24
	INS_M20_B69	462	COG1614	4.00E-190	arCOG04360	CdhC	3.50E-192	K00193	1.20E-201	673.5	592.2	PF03598	4.60E-61	TIGR00316	1.2.99.2 2.3	3.30E-191	-	-
	INS_M21_B37	471	COG1614	3.00E-194	arCOG04360	CdhC	1.10E-195	K00193	4.20E-207	691.5	592.2	PF03598	1.80E-65	TIGR00316	1.2.99.2 2.3	1.70E-204	2250277	Thermoprotei_archaeon
	INS_M34_B48	208	COG1614	1.00E-64	arCOG04360	CdhC	2.10E-69	K00193	1.40E-76	261.3	592.2	-	-	TIGR00316	1.2.99.2 2.3	8.20E-77	-	-
	UWMA_0234	277	COG1614	6.70E-106	arCOG04360	CdhC	5.60E-106	K00193	8.10E-115	387.3	592.2	-	-	TIGR00316	1.2.99.2 2.3	1.80E-112	-	-
	3300014913_21	409	COG2069	7.30E-111	arCOG01980	CdhD	1.00E-129	K00194	1.80E-119	401.7	174.5	PF03599	4.80E-55	TIGR00381	1.2.99.2	2.60E-119	2056630	Candidatus_Korarchaeota_archaeon
	3300024423_7	404	COG2069	1.20E-112	arCOG01980	CdhD	8.50E-130	K00194	9.90E-121	405.8	174.5	PF03599	3.40E-57	TIGR00381	1.2.99.2	4.20E-127	2056630	Candidatus_Korarchaeota_archaeon
	B10_G17	403	COG2069	7.80E-118	arCOG01980	CdhD	1.30E-134	K00194	4.00E-126	423.5	174.5	PF03599	3.50E-59	TIGR00381	1.2.99.2	3.00E-130	2056630	Candidatus_Korarchaeota_archaeon
	B14_G2	403	COG2069	7.80E-118	arCOG01980	CdhD	1.30E-134	K00194	4.00E-126	423.5	174.5	PF03599	3.50E-59	TIGR00381	1.2.99.2	3.00E-130	2056630	Candidatus_Korarchaeota_archaeon
	B15_G15	404	COG2069	1.30E-113	arCOG01980	CdhD	7.90E-132	K00194	4.10E-122	410.4	174.5	PF03599	2.20E-59	TIGR00381	1.2.99.2	1.10E-129	2056630	Candidatus_Korarchaeota_archaeon
	B41_G2	404	COG2069	2.10E-113	arCOG01980	CdhD	8.80E-130	K00194	4.90E-122	410.1	174.5	PF03599	1.50E-59	TIGR00381	1.2.99.2	5.70E-128	2056630	Candidatus_Korarchaeota_archaeon
	B51_G1	404	COG2069	2.10E-113	arCOG01980	CdhD	8.80E-130	K00194	4.90E-122	410.1	174.5	PF03599	1.50E-59	TIGR00381	1.2.99.2	5.70E-128	2056630	Candidatus_Korarchaeota_archaeon
	Flange_M5_B13	144	COG2069	1.60E-44	arCOG01980	CdhD	1.20E-49	K00194	9.60E-50	172.5	174.5	PF03599	1.90E-20	TIGR00381	1.2.99.2	2.40E-48	-	-
cdhD	Flange_M5_B4	412	COG2069	1.00E-110	arCOG01980	CdhD	2.10E-136	K00194	1.80E-124	418.1	174.5	PF03599	1.60E-59	TIGR00381	1.2.99.2	2.60E-136	2026714	Candidatus_Bathyarchaeota_archaeon
	Flange_M5_B4	339	COG2069	2.50E-88	arCOG01980	CdhD	4.40E-67	K00194	2.50E-91	309.1	174.5	PF03599	1.30E-40	TIGR00381	1.2.99.2	2.10E-75	-	-
	GS19_ROV16_BS03_Bin_00077	412	COG2069	2.00E-110	arCOG01980	CdhD	1.60E-136	K00194	4.10E-124	416.9	174.5	PF03599	2.10E-59	TIGR00381	1.2.99.2	2.90E-136	2026714	Candidatus_Bathyarchaeota_archaeon
	INS_M11_B49	335	COG2069	2.20E-84	arCOG01980	CdhD	1.90E-63	K00194	2.60E-86	292.7	174.5	PF03599	3.60E-39	TIGR00381	1.2.99.2	6.20E-70	-	-
	INS_M11_B49	396	COG2069	2.40E-113	arCOG01980	CdhD	7.70E-140	K00194	2.30E-127	427.6	174.5	PF03599	8.60E-63	TIGR00381	1.2.99.2	5.40E-138	2026714	Candidatus_Bathyarchaeota_archaeon
	INS_M20_B145	335	COG2069	4.80E-84	arCOG01980	CdhD	3.50E-63	K00194	5.30E-86	291.7	174.5	PF03599	3.10E-38	TIGR00381	1.2.99.2	6.70E-69	-	-
	INS_M20_B145	396	COG2069	8.10E-114	arCOG01980	CdhD	5.40E-140	K00194	1.40E-127	428.3	174.5	PF03599	3.70E-62	TIGR00381	1.2.99.2	4.00E-138	2026714	Candidatus_Bathyarchaeota_archaeon
	INS_M20_B155	339	COG2069	1.20E-86	arCOG01980	CdhD	5.60E-66	K00194	1.20E-89	303.7	174.5	PF03599	1.10E-40	TIGR00381	1.2.99.2	2.50E-75	-	-
	INS_M20_B155	344	COG2069	3.50E-109	arCOG01980	CdhD	2.90E-131	K00194	3.90E-124	417	174.5	PF03599	1.20E-59	TIGR00381	1.2.99.2	2.00E-133	-	-
	INS_M20_B155	116	COG2069	7.30E-27	arCOG01980	CdhD	2.30E-17	K00194	5.60E-27	97.7	174.5	PF03599	5.30E-09	TIGR00381	1.2.99.2	2.10E-17	2026714	Candidatus_Bathyarchaeota_archaeon

INS_M20_B69	408	COG2069	1.70E-112	arCOG01980	CdhD	2.20E-130	K00194	3.40E-120	404	174.5	PF03599	2.40E-55	TIGR00381	1.2.99.2	2.50E-120	2056630	Candidatus_Korarchaeota_archaeon
INS_M21_B31	354	COG2069	3.30E-118	arCOG01980	CdhD	3.30E-133	K00194	9.60E-128	428.9	174.5	PF03599	3.40E-71	TIGR00381	1.2.99.2	4.80E-140	2026714	Candidatus_Bathyarchaeota_archaeon
INS_M21_B37	396	COG2069	2.40E-113	arCOG01980	CdhD	7.70E-140	K00194	2.30E-127	427.6	174.5	PF03599	8.60E-63	TIGR00381	1.2.99.2	5.40E-138	2026714	Candidatus_Bathyarchaeota_archaeon
INS_M21_B37	335	COG2069	2.20E-84	arCOG01980	CdhD	1.90E-63	K00194	2.60E-86	292.7	174.5	PF03599	3.60E-39	TIGR00381	1.2.99.2	6.20E-70	-	-
INS_M22_B49	335	COG2069	2.70E-84	arCOG01980	CdhD	1.20E-63	K00194	2.40E-86	292.8	174.5	PF03599	3.20E-39	TIGR00381	1.2.99.2	5.30E-70	-	-
INS_M29_B128	404	COG2069	2.00E-114	arCOG01980	CdhD	1.30E-132	K00194	1.30E-123	415.2	174.5	PF03599	2.00E-60	TIGR00381	1.2.99.2	1.10E-129	2056630	Candidatus_Korarchaeota_archaeon
INS_M34_B48	335	COG2069	1.80E-84	arCOG01980	CdhD	9.00E-64	K00194	1.20E-86	293.7	174.5	PF03599	1.60E-38	TIGR00381	1.2.99.2	8.90E-70	-	-
INS_M34_B48	396	COG2069	8.10E-114	arCOG01980	CdhD	5.40E-140	K00194	1.40E-127	428.3	174.5	PF03599	3.70E-62	TIGR00381	1.2.99.2	4.00E-138	2026714	Candidatus_Bathyarchaeota_archaeon
UWMA_0234	403	COG2069	1.00E-112	arCOG01980	CdhD	2.20E-131	K00194	4.90E-121	406.8	174.5	PF03599	1.80E-57	TIGR00381	1.2.99.2	2.30E-125	-	-
INS_M29_B128	470	COG1456	1.40E-99	arCOG01979	CdhE	1.10E-184	K00197	1.90E-174	584	181	PF03599	1.40E-131	TIGR00381	1.2.99.2	6.00E-08	2056630	Candidatus_Korarchaeota_archaeon
INS_M29_B128	124	COG4871	6.90E-28	arCOG00297	-	5.40E-18	K00197	2.70E-13	52.9	181	PF04060	9.20E-12	TIGR01944	-	1.00E-05	2056630	Candidatus_Korarchaeota_archaeon
3300014887_59	215	-	-	-	-	-	K00197	5.90E-05	25.4	181	-	-	-	-	-	2056630	Candidatus_Korarchaeota_archaeon
3300014887_59	297	COG1456	3.10E-67	arCOG01979	CdhE	1.00E-113	K00197	2.90E-107	362.6	181	PF03599	1.20E-99	-	-	-	2056630	Candidatus_Korarchaeota_archaeon
3300014913_21	468	COG1456	2.00E-90	arCOG01979	CdhE	1.60E-168	K00197	5.00E-166	556.3	181	PF03599	3.50E-122	TIGR01944	-	0.00011	-	-
3300014913_21	191	COG4871	3.60E-26	arCOG00297	-	1.00E-19	K00197	2.00E-15	59.9	181	PF04060	4.10E-12	TIGR01944	-	0.00045	2250277	Thermoprotei_archaeon
3300014913_21	186	COG4871	2.00E-48	arCOG00297	-	1.70E-40	K00197	4.30E-16	62.1	181	PF04060	1.60E-12	TIGR01944	-	0.00013	2011131	ANME-1_cluster_archaeon_ex4572_4
3300014914_102	215	-	-	-	-	-	K00197	3.60E-05	26.1	181	-	-	-	-	-	2056630	Candidatus_Korarchaeota_archaeon
3300024423_7	218	COG4871	2.50E-30	arCOG00297	-	2.70E-23	K00197	1.50E-16	63.6	181	PF04060	3.80E-13	-	-	-	2056630	Candidatus_Korarchaeota_archaeon
3300024423_7	197	COG4871	1.20E-37	arCOG00297	-	8.50E-29	K00197	3.10E-13	52.7	181	PF04060	1.00E-10	TIGR01944	-	3.90E-06	2056630	Candidatus_Korarchaeota_archaeon

cdhE

B10_G17	215	-	-	-	-	-	K00197	5.90E-05	25.4	181	-	-	-	-	-	2056630	Candidatus_Korarchaeota_archaeon
B10_G17	297	COG1456	3.10E-67	arCOG01979	CdhE	1.00E-113	K00197	2.90E-107	362.6	181	PF03599	1.20E-99	-	-	-	2056630	Candidatus_Korarchaeota_archaeon
B14_G2	215	-	-	-	-	-	K00197	3.60E-05	26.1	181	-	-	-	-	-	2056630	Candidatus_Korarchaeota_archaeon
B14_G2	472	COG1456	3.20E-96	arCOG01979	CdhE	1.90E-184	K00197	5.50E-173	579.2	181	PF03599	1.00E-135	TIGR00381	1.2.99.2	1.20E-08	2056630	Candidatus_Korarchaeota_archaeon
B14_G2	192	COG4871	4.10E-34	arCOG00297	-	1.50E-22	K00197	3.70E-13	52.4	181	PF04060	1.50E-13	TIGR01944	-	2.40E-05	2056630	Candidatus_Korarchaeota_archaeon
B15_G15	130	COG1456	2.30E-51	arCOG01979	CdhE	1.50E-51	K00197	3.00E-55	191.1	181	PF03599	2.20E-41	-	-	-	2056630	Candidatus_Korarchaeota_archaeon
B15_G15	193	COG4871	5.50E-30	arCOG00297	-	1.80E-20	K00197	5.10E-15	58.6	181	PF04060	7.90E-11	-	-	-	2056630	Candidatus_Korarchaeota_archaeon
B15_G15	130	COG4871	7.30E-26	arCOG00297	-	2.40E-18	K00197	1.10E-12	50.8	181	PF04060	5.30E-11	TIGR01944	-	3.60E-06	2056630	Candidatus_Korarchaeota_archaeon
B41_G2	470	COG1456	1.30E-100	arCOG01979	CdhE	1.80E-185	K00197	6.50E-177	592.2	181	PF03599	5.60E-131	TIGR00381	1.2.99.2	2.80E-08	2056630	Candidatus_Korarchaeota_archaeon
B41_G2	197	COG4871	5.60E-36	arCOG00297	-	5.30E-29	K00197	2.60E-13	52.9	181	PF04060	1.00E-10	TIGR01944	-	6.10E-06	2056630	Candidatus_Korarchaeota_archaeon
B51_G1	409	COG1456	9.70E-76	arCOG01979	CdhE	1.30E-155	K00197	2.70E-151	507.7	181	PF03599	5.10E-131	TIGR00381	1.2.99.2	4.10E-08	2056630	Candidatus_Korarchaeota_archaeon
B51_G1	37	COG4871	1.70E-14	arCOG01979	CdhE	1.60E-13	K00197	1.20E-13	54	181	PF04060	2.00E-11	-	-	-	2056630	Candidatus_Korarchaeota_archaeon
B8_G17	78	COG1456	3.00E-22	arCOG01979	CdhE	3.60E-22	K00197	3.40E-25	92.1	181	PF03599	4.60E-14	-	-	-	2056630	Candidatus_Korarchaeota_archaeon
B8_G17	197	COG4871	1.50E-38	arCOG00297	-	2.10E-30	K00197	2.50E-13	53	181	PF04060	1.00E-10	TIGR01944	-	5.80E-06	2056630	Candidatus_Korarchaeota_archaeon
Flange_M5_B13	470	COG1456	2.80E-94	arCOG01979	CdhE	4.90E-177	K00197	7.90E-173	578.7	181	PF03599	1.50E-128	TIGR00381	1.2.99.2	5.60E-06	-	-
Flange_M5_B13	197	COG4871	4.70E-36	arCOG00297	-	6.30E-29	K00197	1.60E-12	50.4	181	PF04060	2.40E-11	TIGR01944	-	6.70E-06	2056630	Candidatus_Korarchaeota_archaeon
Flange_M5_B24	286	COG1456	9.20E-56	arCOG01979	CdhE	4.90E-80	K00197	9.70E-93	314.7	181	PF03599	1.10E-69	-	-	-	1779368	Candidatus_Bathyarchaeota_archaeon_B24
Flange_M5_B24	180	COG4871	5.80E-34	arCOG00297	-	1.20E-20	K00197	1.10E-16	64.1	181	PF04060	9.00E-15	TIGR01944	-	0.00021	2056630	Candidatus_Korarchaeota_archaeon
Flange_M5_B4	180	COG4871	2.90E-31	arCOG00297	-	1.00E-22	K00197	8.90E-08	34.7	181	PF04060	8.20E-06	-	-	-	-	-
Flange_M5_B4	468	COG1456	1.60E-92	arCOG01979	CdhE	2.00E-187	K00197	1.10E-172	578.2	181	PF03599	1.20E-132	TIGR00381	1.2.99.2	1.90E-07	1779368	Candidatus_Bathyarchaeota_archaeon_B24
Flange_M5_B4	451	COG1456	7.40E-92	arCOG01979	CdhE	8.00E-132	K00197	2.00E-158	531.2	181	PF03599	1.80E-105	TIGR01944	-	4.30E-05	1779368	Candidatus_Bathyarchaeota_archaeon_B24
GS19_ROV16_BS03_Bin_00077	190	COG4871	1.10E-31	arCOG00297	-	2.10E-23	K00197	8.40E-14	54.5	181	PF04060	5.60E-13	TIGR01944	-	0.00032	2056630	Candidatus_Korarchaeota_archaeon
GS19_ROV16_BS03_Bin_00077	423	COG1456	1.60E-73	arCOG01979	CdhE	3.70E-115	K00197	5.10E-141	473.9	181	PF03599	2.30E-104	-	-	-	2026714	Candidatus_Bathyarchaeota_archaeon
INS_M11_B49	456	COG1456	7.10E-90	arCOG01979	CdhE	3.30E-138	K00197	1.20E-159	535.3	181	PF03599	5.50E-99	TIGR01944	-	0.00012	1779368	Candidatus_Bathyarchaeota_archaeon_B24
INS_M11_B49	464	COG1456	3.40E-93	arCOG01979	CdhE	1.80E-180	K00197	2.90E-171	573.6	181	PF03599	9.40E-132	TIGR00381	1.2.99.2	1.30E-07	-	-
INS_M20_B145	180	COG4871	5.70E-34	arCOG00297	-	1.10E-20	K00197	1.30E-16	63.8	181	PF04060	8.80E-15	TIGR01944	-	0.00024	2026714	Candidatus_Bathyarchaeota_archaeon

	INS_M20_B145	456	COG1456	9.50E-91	arCOG01979	CdhE	4.70E-139	K00197	1.90E-160	537.9	181	PF03599	2.00E-99	TIGR01944	-	0.00012	1779368	Candidatus_Bathyarchaeota_archaeon_B24
	INS_M20_B145	464	COG1456	1.80E-93	arCOG01979	CdhE	6.50E-181	K00197	1.20E-171	574.8	181	PF03599	1.00E-131	TIGR00381	1.2.99.2	2.60E-07	-	-
	INS_M20_B155	451	COG1456	2.50E-91	arCOG01979	CdhE	3.30E-132	K00197	1.70E-158	531.5	181	PF03599	5.70E-105	TIGR01944	-	4.60E-05	1779368	Candidatus_Bathyarchaeota_archaeon_B24
	INS_M20_B155	468	COG1456	3.10E-91	arCOG01979	CdhE	2.50E-186	K00197	6.80E-172	575.6	181	PF03599	5.60E-132	TIGR00381	1.2.99.2	2.10E-07	1779368	Candidatus_Bathyarchaeota_archaeon_B24
	INS_M20_B155	379	COG1456	1.30E-65	arCOG06909	CdhE	2.80E-38	K00197	4.00E-35	124.8	181	PF03599	2.30E-17	TIGR02700	-	3.20E-07	-	-
	INS_M20_B155	452	COG1456	9.00E-98	arCOG01979	CdhE	8.50E-144	K00197	1.60E-166	557.9	181	PF03599	2.90E-111	TIGR01944	-	5.90E-05	1779368	Candidatus_Bathyarchaeota_archaeon_B24
	INS_M20_B69	468	COG1456	4.10E-90	arCOG01979	CdhE	3.40E-166	K00197	3.80E-165	553.4	181	PF03599	1.80E-119	TIGR01944	-	0.00011	2056630	Candidatus_Korarchaeota_archaeon
	INS_M20_B69	197	COG4871	7.40E-26	arCOG00297	-	3.00E-21	K00197	2.40E-15	59.6	181	PF04060	4.60E-12	TIGR01944	-	9.00E-06	2250277	Thermoprotei_archaeon
	INS_M21_B31	60	COG1456	8.00E-19	arCOG01979	CdhE	3.20E-18	K00197	3.00E-20	75.8	181	PF03599	2.00E-12	-	-	-	-	-
	INS_M21_B37	180	COG4871	5.80E-34	arCOG00297	-	1.20E-20	K00197	1.10E-16	64.1	181	PF04060	9.00E-15	TIGR01944	-	0.00021	2056630	Candidatus_Korarchaeota_archaeon
	INS_M21_B37	464	COG1456	3.30E-93	arCOG01979	CdhE	1.30E-180	K00197	2.60E-171	573.7	181	PF03599	8.30E-132	TIGR00381	1.2.99.2	1.30E-07	-	-
	INS_M21_B37	456	COG1456	7.10E-90	arCOG01979	CdhE	3.30E-138	K00197	1.20E-159	535.3	181	PF03599	5.50E-99	TIGR01944	-	0.00012	1779368	Candidatus_Bathyarchaeota_archaeon_B24
	INS_M22_B49	378	COG1456	1.70E-70	arCOG01979	CdhE	7.70E-148	K00197	2.70E-139	468.2	181	PF03599	3.30E-126	TIGR00284	-	0.00021	1700836	Candidatus_Bathyarchaeota_archaeon_B24
	INS_M34_B48	456	COG1456	1.90E-90	arCOG01979	CdhE	1.80E-138	K00197	3.20E-160	537.2	181	PF03599	2.50E-99	TIGR01944	-	0.00013	1779368	Candidatus_Bathyarchaeota_archaeon_B24
	INS_M34_B48	464	COG1456	3.60E-93	arCOG01979	CdhE	1.10E-180	K00197	2.20E-171	574	181	PF03599	1.50E-131	TIGR00381	1.2.99.2	2.60E-07	-	-
	INS_M34_B48	180	COG4871	5.80E-34	arCOG00297	-	1.20E-20	K00197	1.10E-16	64.1	181	PF04060	9.00E-15	TIGR01944	-	0.00021	2056630	Candidatus_Korarchaeota_archaeon
	UWMA_0234	470	COG1456	6.20E-95	arCOG01979	CdhE	7.30E-179	K00197	5.40E-173	579.3	181	PF03599	3.30E-131	TIGR00381	1.2.99.2	3.80E-07	-	-
	INS_M29_B128	333	-	-	arCOG02410	Mer	6.90E-64	K00320	8.20E-124	416.7	218.3	PF00296	1.10E-67	TIGR03555	1.5.98.2	1.00E-123	-	-
	3300014913_21	329	-	-	arCOG02410	Mer	2.20E-65	K00320	2.10E-122	412.1	218.3	PF00296	9.20E-64	TIGR03555	1.5.98.2	9.50E-123	-	-
	3300014887_59	332	-	-	arCOG02410	Mer	1.80E-75	K00320	2.70E-133	447.9	218.3	PF00296	6.10E-69	TIGR03555	1.5.98.2	5.30E-141	2056630	Candidatus_Korarchaeota_archaeon
	3300024423_7	332	-	-	arCOG02410	Mer	4.80E-64	K00320	6.60E-125	420.3	218.3	PF00296	9.90E-69	TIGR03555	1.5.98.2	1.00E-125	2056630	Candidatus_Korarchaeota_archaeon
	B10_G17	332	-	-	arCOG02410	Mer	1.80E-75	K00320	2.70E-133	447.9	218.3	PF00296	6.10E-69	TIGR03555	1.5.98.2	5.30E-141	2056630	Candidatus_Korarchaeota_archaeon
	B15_G15	346	-	-	arCOG02410	Mer	2.60E-64	K00320	1.90E-124	418.8	218.3	PF00296	1.70E-66	TIGR03555	1.5.98.2	1.40E-124	2056630	Candidatus_Korarchaeota_archaeon
	B51_G1	89	-	-	arCOG02410	Mer	2.90E-06	K00320	1.10E-26	97.4	218.3	PF00296	2.00E-05	TIGR03555	1.5.98.2	1.20E-28	2056630	Candidatus_Korarchaeota_archaeon
	B8_G17	333	-	-	arCOG02410	Mer	6.70E-64	K00320	1.30E-126	425.9	218.3	PF00296	2.60E-68	TIGR03555	1.5.98.2	2.40E-125	2056630	Candidatus_Korarchaeota_archaeon
	Flange_M5_B13	274	-	-	arCOG02410	Mer	1.10E-49	K00320	8.80E-104	350.8	218.3	PF00296	3.50E-54	TIGR03555	1.5.98.2	3.30E-104	-	-
	Flange_M5_B24	333	-	-	arCOG02410	Mer	1.90E-63	K00320	8.30E-131	439.7	218.3	PF00296	2.30E-62	TIGR03555	1.5.98.2	3.70E-138	1698273	candidate_division_MSBL1_archaeon_SCGC-AAA261D19
	Flange_M5_B24	327	-	-	arCOG02410	Mer	6.90E-69	K00320	1.20E-122	412.9	218.3	PF00296	5.80E-63	TIGR03555	1.5.98.2	4.50E-126	-	-
	Flange_M5_B4	327	-	-	arCOG02410	Mer	3.60E-67	K00320	2.90E-123	414.9	218.3	PF00296	6.30E-66	TIGR03555	1.5.98.2	1.30E-126	2012530	Candidatus_Geothermarchaeota_archaeon_ex4572_27
	INS_M11_B49	188	-	-	arCOG02410	Mer	3.50E-27	K00320	6.20E-62	213.3	218.3	PF00296	7.60E-22	TIGR03555	1.5.98.2	1.60E-64	-	-
mer	INS_M11_B49	128	-	-	arCOG02410	Mer	1.50E-28	K00320	4.60E-47	164.4	218.3	PF00296	1.20E-27	TIGR03555	1.5.98.2	8.30E-48	2012530	Candidatus_Geothermarchaeota_archaeon_ex4572_27

	INS_M20_B145	327	-	-	arCOG02410	Mer	6.90E-69	K00320	1.20E-122	412.9	218.3	PF00296	5.80E-63	TIGR03555	1.5.98.2	4.50E-126	-	-
	INS_M20_B155	327	-	-	arCOG02410	Mer	3.60E-67	K00320	2.90E-123	414.9	218.3	PF00296	6.30E-66	TIGR03555	1.5.98.2	1.30E-126	2012530	Candidatus_Geothermarchaeota_archaeon_ex4572_27
	INS_M20_B69	325	-	-	arCOG02410	Mer	1.20E-49	K00320	4.40E-91	309.1	218.3	PF00296	3.50E-53	TIGR03555	1.5.98.2	1.60E-70	2026795	Thaumarchaeota_archaeon
	INS_M20_B69	329	-	-	arCOG02410	Mer	2.90E-64	K00320	1.40E-123	415.9	218.3	PF00296	1.20E-62	TIGR03555	1.5.98.2	8.60E-126	-	-
	INS_M21_B31	250	-	-	arCOG02410	Mer	5.70E-41	K00320	3.10E-93	316.2	218.3	PF00296	3.00E-39	TIGR03555	1.5.98.2	6.40E-99	-	-
	INS_M21_B37	327	-	-	arCOG02410	Mer	6.90E-69	K00320	1.20E-122	412.9	218.3	PF00296	5.80E-63	TIGR03555	1.5.98.2	4.50E-126	-	-
	INS_M34_B48	327	-	-	arCOG02410	Mer	6.90E-69	K00320	1.20E-122	412.9	218.3	PF00296	5.80E-63	TIGR03555	1.5.98.2	4.50E-126	-	-
	UWMA_0234	333	-	-	arCOG02410	Mer	3.40E-66	K00320	9.40E-128	429.6	218.3	PF00296	6.90E-69	TIGR03555	1.5.98.2	3.30E-127	-	-
	INS_M29_B128	309	COG1927	5.10E-119	arCOG04382	Mtd	3.90E-108	K00319	3.30E-121	407.3	252.8	PF01993	2.80E-120	-	-	-	-	-
	3300014913_21	98	COG1927	1.40E-37	arCOG04382	Mtd	1.50E-33	K00319	2.00E-38	135.7	252.8	PF01993	3.30E-39	-	-	-	1685127	miscellaneous_Crenarchaeota_group-15_archaeon_DG-45
mtd	3300024423_7	278	COG1927	4.60E-119	arCOG04382	Mtd	5.60E-108	K00319	7.80E-121	406	252.8	PF01993	3.50E-120	-	-	-	2056630	Candidatus_Korarchaeota_archaeon
	B10_G17	282	COG1927	9.00E-112	arCOG04382	Mtd	4.90E-99	K00319	3.00E-114	384.5	252.8	PF01993	2.10E-114	-	-	-	2056630	Candidatus_Korarchaeota_archaeon
	B15_G15	278	COG1927	1.40E-120	arCOG04382	Mtd	2.50E-110	K00319	2.80E-122	410.8	252.8	PF01993	1.00E-121	-	-	-	2056630	Candidatus_Korarchaeota_archaeon
	B41_G2	278	COG1927	1.40E-120	arCOG04382	Mtd	2.50E-110	K00319	2.80E-122	410.8	252.8	PF01993	1.00E-121	-	-	-	2056630	Candidatus_Korarchaeota_archaeon

	B41_G2	280	COG1927	1.10E-120	arCOG04382	Mtd	4.60E-109	K00319	2.90E-122	410.8	252.8	PF01993	1.50E-122	-	-	-	2056630	Candidatus_Korarchaeota_archaeon
	B8_G17	284	COG1927	1.90E-120	arCOG04382	Mtd	1.20E-108	K00319	4.50E-122	410.1	252.8	PF01993	2.80E-122	-	-	-	2056630	Candidatus_Korarchaeota_archaeon
	Flange_M5_B13	277	COG1927	6.90E-120	arCOG04382	Mtd	4.40E-108	K00319	1.60E-121	408.3	252.8	PF01993	2.40E-120	-	-	-	2056630	Candidatus_Korarchaeota_archaeon
	Flange_M5_B4	277	COG1927	1.80E-113	arCOG04382	Mtd	1.40E-99	K00319	7.40E-116	389.7	252.8	PF01993	8.00E-116	-	-	-	-	-
	HyVt-231	284	COG1927	1.90E-120	arCOG04382	Mtd	1.20E-108	K00319	4.50E-122	410.1	252.8	PF01993	2.80E-122	-	-	-	2056630	Candidatus_Korarchaeota_archaeon
	INS_M11_B49	279	COG1927	1.10E-107	arCOG04382	Mtd	1.40E-95	K00319	1.70E-110	372.2	252.8	PF01993	6.30E-110	-	-	-	2056630	Candidatus_Korarchaeota_archaeon
	INS_M20_B145	279	COG1927	7.40E-108	arCOG04382	Mtd	1.40E-95	K00319	1.00E-110	372.8	252.8	PF01993	4.50E-110	-	-	-	2056630	Candidatus_Korarchaeota_archaeon
	INS_M20_B155	277	COG1927	1.80E-113	arCOG04382	Mtd	1.40E-99	K00319	7.40E-116	389.7	252.8	PF01993	8.00E-116	-	-	-	-	-
	INS_M20_B69	276	COG1927	2.20E-112	arCOG04382	Mtd	4.40E-100	K00319	1.50E-114	385.4	252.8	PF01993	3.30E-114	-	-	-	2056630	Candidatus_Korarchaeota_archaeon
	INS_M21_B31	276	COG1927	6.00E-113	arCOG04382	Mtd	7.70E-101	K00319	2.10E-115	388.2	252.8	PF01993	4.30E-117	-	-	-	2056630	Candidatus_Korarchaeota_archaeon
	INS_M21_B37	279	COG1927	7.40E-108	arCOG04382	Mtd	1.40E-95	K00319	1.00E-110	372.8	252.8	PF01993	4.50E-110	-	-	-	2056630	Candidatus_Korarchaeota_archaeon
	INS_M22_B49	336	COG1927	1.20E-107	arCOG04382	Mtd	2.40E-95	K00319	1.60E-110	372.3	252.8	PF01993	8.70E-110	-	-	-	2056630	Candidatus_Korarchaeota_archaeon
	INS_M34_B48	336	COG1927	1.20E-107	arCOG04382	Mtd	2.40E-95	K00319	1.60E-110	372.3	252.8	PF01993	8.70E-110	-	-	-	2056630	Candidatus_Korarchaeota_archaeon
	UWMA_0234	278	COG1927	2.00E-118	arCOG04382	Mtd	3.60E-107	K00319	5.40E-121	406.6	252.8	PF01993	7.30E-120	-	-	-	-	-
	3300014913_21	318	COG3252	1.50E-105	arCOG02675	Mch	7.00E-98	K01499	4.60E-102	344.9	284.6	PF02289	4.20E-108	TIGR03120	3.5.4.27	5.00E-105	2056630	Candidatus_Korarchaeota_archaeon
	3300024423_7	319	COG3252	2.00E-105	arCOG02675	Mch	1.40E-96	K01499	7.40E-102	344.2	284.6	PF02289	4.70E-105	TIGR03120	3.5.4.27	7.10E-100	2056630	Candidatus_Korarchaeota_archaeon
	B10_G17	313	COG3252	2.30E-117	arCOG02675	Mch	1.70E-105	K01499	6.80E-113	380.5	284.6	PF02289	5.60E-118	TIGR03120	3.5.4.27	2.00E-118	2056630	Candidatus_Korarchaeota_archaeon
	B14_G2	313	COG3252	2.30E-117	arCOG02675	Mch	1.70E-105	K01499	6.80E-113	380.5	284.6	PF02289	5.60E-118	TIGR03120	3.5.4.27	2.00E-118	2056630	Candidatus_Korarchaeota_archaeon
	B15_G15	348	COG3252	2.00E-55	arCOG02675	Mch	6.60E-47	K01499	5.20E-53	183.6	284.6	PF02289	2.30E-58	TIGR03120	3.5.4.27	1.80E-58	2056630	Candidatus_Korarchaeota_archaeon
	B15_G15	319	COG3252	8.30E-105	arCOG02675	Mch	2.30E-96	K01499	3.60E-101	341.9	284.6	PF02289	5.60E-105	TIGR03120	3.5.4.27	3.10E-99	2056630	Candidatus_Korarchaeota_archaeon
	B15_G15	48	COG3252	0.00022	-	-	-	K01499	0.00052	22.6	284.6	PF02289	2.10E-05	TIGR03120	3.5.4.27	0.00013	2056630	Candidatus_Korarchaeota_archaeon
	B41_G2	319	COG3252	1.10E-103	arCOG02675	Mch	1.00E-96	K01499	7.90E-100	337.5	284.6	PF02289	4.30E-103	TIGR03120	3.5.4.27	1.40E-98	2056630	Candidatus_Korarchaeota_archaeon
	B51_G1	174	COG3252	2.20E-49	arCOG02675	Mch	1.90E-45	K01499	3.20E-48	167.9	284.6	PF02289	1.90E-49	TIGR03120	3.5.4.27	4.10E-47	2056630	Candidatus_Korarchaeota_archaeon
	B51_G1	122	COG3252	1.50E-37	arCOG02675	Mch	1.30E-33	K01499	1.50E-35	126.2	284.6	PF02289	1.40E-36	TIGR03120	3.5.4.27	1.40E-33	2056630	Candidatus_Korarchaeota_archaeon
	B51_G1	348	COG3252	1.20E-53	arCOG02675	Mch	8.90E-43	K01499	2.90E-51	177.9	284.6	PF02289	8.60E-57	TIGR03120	3.5.4.27	1.30E-57	2056630	Candidatus_Korarchaeota_archaeon
	B8_G17	319	COG3252	6.80E-106	arCOG02675	Mch	1.90E-97	K01499	3.30E-101	342.1	284.6	PF02289	4.10E-105	TIGR03120	3.5.4.27	5.10E-100	2056630	Candidatus_Korarchaeota_archaeon
mch	Flange_M5_B13	129	COG3252	8.70E-31	arCOG02675	Mch	6.90E-26	K01499	1.60E-29	106.5	284.6	PF02289	8.80E-31	TIGR03120	3.5.4.27	1.20E-28	2056630	Candidatus_Korarchaeota_archaeon
	Flange_M5_B4	318	COG3252	8.50E-109	arCOG02675	Mch	4.10E-96	K01499	5.60E-106	357.7	284.6	PF02289	5.00E-110	TIGR03120	3.5.4.27	3.30E-106	2026714	Candidatus_Bathyarchaeota_archaeon
	HyVt-231	174	COG3252	2.50E-51	arCOG02675	Mch	2.50E-45	K01499	3.40E-49	171.1	284.6	PF02289	1.60E-50	TIGR03120	3.5.4.27	9.90E-48	-	-
	INS_M11_B49	320	COG3252	5.00E-107	arCOG02675	Mch	2.90E-97	K01499	3.90E-103	348.4	284.6	PF02289	2.00E-108	TIGR03120	3.5.4.27	1.70E-106	2026714	Candidatus_Bathyarchaeota_archaeon
	INS_M20_B145	320	COG3252	4.50E-107	arCOG02675	Mch	5.90E-97	K01499	4.20E-103	348.3	284.6	PF02289	2.30E-108	TIGR03120	3.5.4.27	2.40E-106	2026714	Candidatus_Bathyarchaeota_archaeon
	INS_M20_B155	318	COG3252	8.50E-109	arCOG02675	Mch	4.10E-96	K01499	5.60E-106	357.7	284.6	PF02289	5.00E-110	TIGR03120	3.5.4.27	3.30E-106	2026714	Candidatus_Bathyarchaeota_archaeon
	INS_M20_B69	318	COG3252	1.70E-103	arCOG02675	Mch	2.10E-95	K01499	4.50E-101	341.6	284.6	PF02289	8.70E-106	TIGR03120	3.5.4.27	2.00E-100	2056630	Candidatus_Korarchaeota_archaeon
	INS_M21_B31	319	COG3252	4.80E-111	arCOG02675	Mch	2.20E-101	K01499	1.30E-107	363.1	284.6	PF02289	5.10E-113	TIGR03120	3.5.4.27	1.40E-107	2026714	Candidatus_Bathyarchaeota_archaeon
	INS_M21_B37	320	COG3252	5.00E-107	arCOG02675	Mch	2.90E-97	K01499	3.90E-103	348.4	284.6	PF02289	2.00E-108	TIGR03120	3.5.4.27	1.70E-106	2026714	Candidatus_Bathyarchaeota_archaeon
	INS_M22_B49	320	COG3252	5.00E-107	arCOG02675	Mch	2.90E-97	K01499	3.90E-103	348.4	284.6	PF02289	2.00E-108	TIGR03120	3.5.4.27	1.70E-106	2026714	Candidatus_Bathyarchaeota_archaeon
	INS_M29_B128	318	COG3252	1.70E-106	arCOG02675	Mch	6.00E-98	K01499	1.60E-101	343.1	284.6	PF02289	6.90E-105	TIGR03120	3.5.4.27	4.90E-99	2056630	Candidatus_Korarchaeota_archaeon
	UWMA_0234	228	COG3252	7.00E-76	arCOG02675	Mch	1.70E-72	K01499	2.80E-73	250.2	284.6	PF02289	6.00E-77	TIGR03120	3.5.4.27	4.00E-75	-	-
	UWMA_0234	296	COG3252	3.00E-41	arCOG02675	Mch	5.70E-33	K01499	5.10E-39	137.6	284.6	PF02289	3.20E-44	TIGR03120	3.5.4.27	5.40E-45	-	-
	INS_M29_B128	249	COG2037	8.10E-97	arCOG02695	Ftr	1.90E-79	K00672	1.40E-95	323.4	315.6	PF02741	7.80E-59	TIGR03119	2.3.1.101	6.80E-94	-	-
	3300014887_59	299	COG2037	8.20E-124	arCOG02695	Ftr	1.50E-114	K00672	4.10E-124	417.1	315.6	PF02741	2.30E-60	TIGR03119	2.3.1.101	9.20E-121	2056630	Candidatus_Korarchaeota_archaeon

	3300014914_102	299	COG2037	8.20E-124	arCOG02695	Ftr	1.50E-114	K00672	4.10E-124	417.1	315.6	PF02741	2.30E-60	TIGR03119	2.3.1.101	9.20E-121	2056630	Candidatus_Korarchaeota_archaeon
ftr	3300024423_7	307	COG2037	4.60E-126	arCOG02695	Ftr	1.40E-108	K00672	1.80E-124	418.2	315.6	PF02741	1.20E-60	TIGR03119	2.3.1.101	3.50E-120	2056630	Candidatus_Korarchaeota_archaeon
	B10_G17	299	COG2037	8.20E-124	arCOG02695	Ftr	1.50E-114	K00672	4.10E-124	417.1	315.6	PF02741	2.30E-60	TIGR03119	2.3.1.101	9.20E-121	2056630	Candidatus_Korarchaeota_archaeon
	B14_G2	299	COG2037	8.20E-124	arCOG02695	Ftr	1.50E-114	K00672	4.10E-124	417.1	315.6	PF02741	2.30E-60	TIGR03119	2.3.1.101	9.20E-121	2056630	Candidatus_Korarchaeota_archaeon

	B15_G15	124	COG2037	8.10E-16	arCOG02695	Ftr	8.50E-12	K00672	9.20E-15	58.1	315.6	PF02741	7.10E-17	TIGR03119	2.3.1.101	2.80E-14	2056630	Candidatus_Korarchaeota_archaeon
	B15_G15	307	COG2037	4.20E-126	arCOG02695	Ftr	2.10E-108	K00672	1.80E-124	418.3	315.6	PF02741	1.20E-60	TIGR03119	2.3.1.101	2.50E-120	2056630	Candidatus_Korarchaeota_archaeon
	B41_G2	97	COG2037	3.20E-30	arCOG02695	Ftr	1.90E-27	K00672	3.00E-29	105.6	315.6	PF02741	4.60E-31	TIGR03119	2.3.1.101	4.70E-27	2056630	Candidatus_Korarchaeota_archaeon
	B51_G1	307	COG2037	1.00E-125	arCOG02695	Ftr	9.60E-109	K00672	7.20E-124	416.3	315.6	PF02741	9.30E-61	TIGR03119	2.3.1.101	6.40E-120	2056630	Candidatus_Korarchaeota_archaeon
	B8_G17	100	COG2037	3.00E-30	arCOG02695	Ftr	4.50E-27	K00672	3.00E-29	105.6	315.6	PF02741	4.60E-31	TIGR03119	2.3.1.101	2.00E-26	2056630	Candidatus_Korarchaeota_archaeon
	Flange_M5_B13	290	COG2037	3.00E-117	arCOG02695	Ftr	1.20E-100	K00672	9.80E-116	389.6	315.6	PF02741	1.00E-60	TIGR03119	2.3.1.101	6.20E-114	2056630	Candidatus_Korarchaeota_archaeon
	Flange_M5_B4	302	COG2037	5.40E-137	arCOG02695	Ftr	7.90E-109	K00672	1.60E-134	451.2	315.6	PF02741	1.60E-62	TIGR03119	2.3.1.101	1.40E-129	2012484	Hadesarchaea_archaeon_B3_Hades
	INS_M20_B145	302	COG2037	7.00E-134	arCOG02695	Ftr	2.30E-106	K00672	1.50E-131	441.5	315.6	PF02741	1.10E-62	TIGR03119	2.3.1.101	1.60E-127	2012484	Hadesarchaea_archaeon_B3_Hades
	INS_M20_B69	306	COG2037	6.50E-119	arCOG02695	Ftr	3.70E-102	K00672	1.00E-117	396.1	315.6	PF02741	1.00E-54	TIGR03119	2.3.1.101	1.10E-112	2056630	Candidatus_Korarchaeota_archaeon
	INS_M21_B31	282	COG2037	3.00E-113	arCOG02695	Ftr	5.70E-99	K00672	3.60E-113	381.1	315.6	PF02741	1.20E-59	TIGR03119	2.3.1.101	2.90E-110	2026714	Candidatus_Bathyarchaeota_archaeon
	INS_M21_B37	302	COG2037	7.00E-134	arCOG02695	Ftr	2.30E-106	K00672	1.50E-131	441.5	315.6	PF02741	1.10E-62	TIGR03119	2.3.1.101	1.60E-127	2012484	Hadesarchaea_archaeon_B3_Hades
	INS_M29_B128	56	COG2037	1.50E-18	arCOG02695	Ftr	1.40E-17	K00672	2.20E-18	70	315.6	PF01913	2.40E-20	TIGR03119	2.3.1.101	3.60E-16	2056630	Candidatus_Korarchaeota_archaeon
	INS_M34_B48	37	COG2037	1.80E-10	arCOG02695	Ftr	5.70E-10	K00672	1.60E-10	44.1	315.6	PF02741	2.40E-10	TIGR03119	2.3.1.101	1.00E-10	2026714	Candidatus_Bathyarchaeota_archaeon
	UWMA_0234	307	COG2037	2.30E-125	arCOG02695	Ftr	2.70E-108	K00672	8.10E-124	416.1	315.6	PF02741	3.50E-60	TIGR03119	2.3.1.101	1.80E-119	-	-
	INS_M29_B128	566	COG1229	4.60E-219	arCOG04461	FwdA	4.40E-218	K00200	9.70E-223	743.9	316.7	PF07969	3.90E-77	TIGR03121	1.2.99.5	4.10E-230	-	-
	3300014887_59	566	COG1229	1.30E-238	arCOG04461	FwdA	9.40E-235	K00200	1.80E-240	802.4	316.7	PF07969	8.10E-77	TIGR03121	1.2.99.5	2.80E-248	2056630	Candidatus_Korarchaeota_archaeon
	3300014913_21	558	COG1229	1.10E-201	arCOG04461	FwdA	3.90E-202	K00200	2.50E-209	699.6	316.7	PF07969	4.20E-67	TIGR03121	1.2.99.5	4.90E-216	2056630	Candidatus_Korarchaeota_archaeon
	3300014914_102	274	COG1229	1.00E-107	arCOG04461	FwdA	3.80E-103	K00200	1.00E-107	364.3	316.7	PF07969	6.30E-34	TIGR03121	1.2.99.5	3.80E-111	2056630	Candidatus_Korarchaeota_archaeon
	3300024423_7	566	COG1229	6.00E-221	arCOG04461	FwdA	1.70E-218	K00200	5.40E-224	748	316.7	PF07969	2.90E-78	TIGR03121	1.2.99.5	6.50E-232	2056630	Candidatus_Korarchaeota_archaeon
	B10_G17	566	COG1229	1.30E-238	arCOG04461	FwdA	9.40E-235	K00200	1.80E-240	802.4	316.7	PF07969	8.10E-77	TIGR03121	1.2.99.5	2.80E-248	2056630	Candidatus_Korarchaeota_archaeon
	B14_G2	274	COG1229	1.00E-107	arCOG04461	FwdA	3.80E-103	K00200	1.00E-107	364.3	316.7	PF07969	6.30E-34	TIGR03121	1.2.99.5	3.80E-111	2056630	Candidatus_Korarchaeota_archaeon
	B15_G15	566	COG1229	1.60E-221	arCOG04461	FwdA	3.40E-219	K00200	1.30E-224	750.1	316.7	PF07969	3.90E-78	TIGR03121	1.2.99.5	2.70E-232	2056630	Candidatus_Korarchaeota_archaeon
	B41_G2	225	COG1229	1.00E-81	arCOG04461	FwdA	6.60E-82	K00200	2.40E-82	280.5	316.7	PF07969	2.90E-27	TIGR03121	1.2.99.5	2.60E-85	2056630	Candidatus_Korarchaeota_archaeon
	B51_G1	566	COG1229	9.90E-224	arCOG04461	FwdA	5.00E-220	K00200	3.10E-225	752.1	316.7	PF07969	1.90E-76	TIGR03121	1.2.99.5	1.40E-232	2056630	Candidatus_Korarchaeota_archaeon
fwdA	B8_G17	566	COG1229	4.40E-223	arCOG04461	FwdA	5.20E-222	K00200	6.50E-226	754.4	316.7	PF07969	5.10E-79	TIGR03121	1.2.99.5	7.30E-235	2056630	Candidatus_Korarchaeota_archaeon
	Flange_M5_B13	566	COG1229	2.00E-224	arCOG04461	FwdA	1.50E-221	K00200	5.40E-226	754.6	316.7	PF07969	5.40E-76	TIGR03121	1.2.99.5	1.50E-233	2056630	Candidatus_Korarchaeota_archaeon
	Flange_M5_B24	565	COG1229	3.50E-235	arCOG04461	FwdA	8.40E-240	K00200	8.10E-239	797	316.7	PF07969	1.40E-79	TIGR03121	1.2.99.5	1.60E-244	-	-
	Flange_M5_B4	564	COG1229	2.40E-229	arCOG04461	FwdA	1.80E-230	K00200	5.50E-231	771.1	316.7	PF07969	6.90E-78	TIGR03121	1.2.99.5	2.40E-237	2026714	Candidatus_Bathyarchaeota_archaeon
	INS_M20_B145	564	COG1229	2.40E-230	arCOG04461	FwdA	1.20E-230	K00200	2.90E-232	775.3	316.7	PF07969	2.20E-74	TIGR03121	1.2.99.5	1.40E-241	-	-
	INS_M20_B155	564	COG1229	1.30E-229	arCOG04461	FwdA	5.50E-231	K00200	3.10E-231	771.9	316.7	PF07969	7.60E-78	TIGR03121	1.2.99.5	9.30E-238	2026714	Candidatus_Bathyarchaeota_archaeon
	INS_M20_B69	558	COG1229	5.80E-202	arCOG04461	FwdA	1.50E-201	K00200	2.40E-209	699.7	316.7	PF07969	1.00E-64	TIGR03121	1.2.99.5	4.60E-215	2056630	Candidatus_Korarchaeota_archaeon
	INS_M21_B31	456	COG1229	2.40E-186	arCOG04461	FwdA	1.90E-193	K00200	1.30E-192	644.5	316.7	PF07969	3.60E-60	TIGR03121	1.2.99.5	1.60E-197	-	-
	INS_M21_B37	564	COG1229	1.90E-230	arCOG04461	FwdA	3.50E-231	K00200	1.90E-232	775.9	316.7	PF07969	2.20E-74	TIGR03121	1.2.99.5	6.60E-242	-	-
	INS_M22_B49	564	COG1229	2.10E-230	arCOG04461	FwdA	3.70E-231	K00200	2.80E-232	775.4	316.7	PF07969	9.10E-75	TIGR03121	1.2.99.5	5.60E-242	-	-
	INS_M34_B48	541	COG1229	2.80E-219	arCOG04461	FwdA	3.00E-220	K00200	5.40E-222	741.4	316.7	PF07969	1.90E-74	TIGR03121	1.2.99.5	3.90E-232	-	-
	UWMA_0234	566	COG1229	4.60E-218	arCOG04461	FwdA	2.60E-217	K00200	2.50E-221	739.2	316.7	PF07969	1.40E-74	TIGR03121	1.2.99.5	3.60E-228	-	-
	INS_M29_B128	99	COG1153	1.10E-30	arCOG01499	FwdB	7.00E-27	K00201	1.40E-31	113	156.1	-	-	TIGR03129	1.2.99.5	9.70E-30	2056630	Candidatus_Korarchaeota_archaeon
	3300014913_21	246	COG1153	2.50E-76	arCOG01499	FwdB	1.40E-67	K00201	3.20E-85	289.8	156.1	-	-	TIGR03129	1.2.99.5	2.40E-83	2250277	Thermoprotei_archaeon
	3300014887_59	420	COG1153	9.60E-75	arCOG01499	FwdB	4.70E-79	K00201	1.20E-107	363.8	156.1	PF00384	7.30E-09	TIGR03129	1.2.99.5	7.10E-111	2056630	Candidatus_Korarchaeota_archaeon
	3300014887_59	432	COG1153	9.30E-148	arCOG01499	FwdB	1.80E-161	K00201	7.40E-181	605	156.1	PF00384	8.90E-18	TIGR03129	1.2.99.5	5.50E-181	2056630	Candidatus_Korarchaeota_archaeon
	3300014914_102	432	COG1153	9.30E-148	arCOG01499	FwdB	1.80E-161	K00201	7.40E-181	605	156.1	PF00384	8.90E-18	TIGR03129	1.2.99.5	5.50E-181	2056630	Candidatus_Korarchaeota_archaeon
	3300024423_7	581	COG1153	1.40E-167	arCOG01499	FwdB	9.10E-141	K00201	3.30E-164	550.1	156.1	PF00384	3.60E-14	TIGR03129	1.2.99.5	1.30E-164	2056630	Candidatus_Korarchaeota_archaeon
	B10_G17	420	COG1153	9.60E-75	arCOG01499	FwdB	4.70E-79	K00201	1.20E-107	363.8	156.1	PF00384	7.30E-09	TIGR03129	1.2.99.5	7.10E-111	2056630	Candidatus_Korarchaeota_archaeon
	B10_G17	432	COG1153	9.30E-148	arCOG01499	FwdB	1.80E-161	K00201	7.40E-181	605	156.1	PF00384	8.90E-18	TIGR03129	1.2.99.5	5.50E-181	2056630	Candidatus_Korarchaeota_archaeon
	B14_G2	420	COG1153	6.70E-75	arCOG01499	FwdB	5.60E-79	K00201	1.10E-107	363.8	156.1	PF00384	7.30E-09	TIGR03129	1.2.99.5	1.00E-110	2056630	Candidatus_Korarchaeota_archaeon
fwdB	B14_G2	432	COG1153	9.30E-148	arCOG01499	FwdB	1.80E-161	K00201	7.40E-181	605	156.1	PF00384	8.90E-18	TIGR03129	1.2.99.5	5.50E-181	2056630	Candidatus_Korarchaeota_archaeon

	B15_G15	581	COG1153	1.20E-163	arCOG01499	FwdB	6.30E-140	K00201	1.30E-161	541.5	156.1	PF00384	1.90E-15	TIGR03129	1.2.99.5	2.90E-163	2056630	Candidatus_Korarchaeota_archaeon
	B41_G2	581	COG1153	8.20E-167	arCOG01499	FwdB	4.90E-143	K00201	4.10E-164	549.8	156.1	PF00384	5.60E-16	TIGR03129	1.2.99.5	5.80E-166	2056630	Candidatus_Korarchaeota_archaeon
	B51_G1	581	COG1153	1.80E-167	arCOG01499	FwdB	3.50E-143	K00201	1.80E-165	554.2	156.1	PF00384	1.10E-16	TIGR03129	1.2.99.5	4.10E-166	2056630	Candidatus_Korarchaeota_archaeon
	B8_G17	580	COG1153	1.10E-167	arCOG01499	FwdB	3.60E-149	K00201	1.80E-168	564.2	156.1	PF00384	1.20E-15	TIGR03129	1.2.99.5	3.90E-170	2056630	Candidatus_Korarchaeota_archaeon
	Flange_M5_B13	581	COG1153	2.00E-167	arCOG01499	FwdB	7.70E-142	K00201	8.70E-164	548.7	156.1	PF00384	1.10E-17	TIGR03129	1.2.99.5	4.50E-166	2056630	Candidatus_Korarchaeota_archaeon
	Flange_M5_B4	432	COG1153	3.50E-126	arCOG01499	FwdB	9.10E-142	K00201	1.20E-168	564.8	156.1	PF00384	1.20E-12	TIGR03129	1.2.99.5	9.80E-170	2026714	Candidatus_Bathyarchaeota_archaeon
	INS_M11_B49	430	COG1153	1.00E-121	arCOG01499	FwdB	1.80E-139	K00201	9.10E-170	568.4	156.1	PF00384	2.30E-10	TIGR03129	1.2.99.5	1.80E-170	2026714	Candidatus_Bathyarchaeota_archaeon
	INS_M20_B145	430	COG1153	2.60E-122	arCOG01499	FwdB	2.60E-141	K00201	9.20E-171	571.7	156.1	PF00384	1.10E-10	TIGR03129	1.2.99.5	1.20E-171	2026714	Candidatus_Bathyarchaeota_archaeon
	INS_M20_B155	432	COG1153	3.50E-126	arCOG01499	FwdB	9.10E-142	K00201	1.20E-168	564.8	156.1	PF00384	1.20E-12	TIGR03129	1.2.99.5	9.80E-170	2026714	Candidatus_Bathyarchaeota_archaeon
	INS_M20_B69	569	COG1153	4.70E-159	arCOG01499	FwdB	7.10E-131	K00201	7.20E-163	545.7	156.1	PF01568	1.10E-13	TIGR03129	1.2.99.5	6.40E-157	2250277	Thermoprotei_archaeon
	INS_M21_B31	431	COG1153	2.60E-131	arCOG01499	FwdB	1.10E-154	K00201	2.30E-172	577	156.1	PF00384	4.00E-14	TIGR03129	1.2.99.5	6.80E-175	-	-
	INS_M21_B31	422	COG1153	2.40E-79	arCOG01498	FwdB	9.00E-75	K00201	1.90E-110	373	156.1	-	-	TIGR03129	1.2.99.5	2.30E-116	2056630	Candidatus_Korarchaeota_archaeon
	INS_M21_B37	430	COG1153	2.60E-122	arCOG01499	FwdB	2.60E-141	K00201	9.20E-171	571.7	156.1	PF00384	1.10E-10	TIGR03129	1.2.99.5	1.20E-171	2026714	Candidatus_Bathyarchaeota_archaeon
	INS_M22_B49	258	COG1153	8.40E-84	arCOG01499	FwdB	4.40E-84	K00201	1.10E-97	330.9	156.1	-	-	TIGR03129	1.2.99.5	1.60E-102	-	-
	INS_M34_B48	430	COG1153	2.60E-122	arCOG01499	FwdB	2.60E-141	K00201	9.20E-171	571.7	156.1	PF00384	1.10E-10	TIGR03129	1.2.99.5	1.20E-171	2026714	Candidatus_Bathyarchaeota_archaeon
	UWMA_0234	131	COG1153	1.80E-38	arCOG01499	FwdB	9.80E-35	K00201	2.70E-43	151.7	156.1	-	-	TIGR03129	1.2.99.5	7.20E-41	2056630	Candidatus_Korarchaeota_archaeon

fwdC	INS_M29_B128	286	COG2218	3.00E-73	arCOG00097	FwdC	1.20E-78	K00202	1.10E-74	254.4	102.5	PF01493	3.10E-13	TIGR03122	1.2.99.5	1.50E-69	-	-
	3300014887_59	276	COG2218	1.30E-75	arCOG00097	FwdC	6.60E-86	K00202	1.20E-79	270.6	102.5	PF01493	1.70E-11	TIGR03122	1.2.99.5	6.10E-76	2056630	Candidatus_Korarchaeota_archaeon
	3300014913_21	278	COG2218	1.60E-70	arCOG00097	FwdC	1.80E-67	K00202	2.70E-70	240	102.5	PF01493	8.30E-15	TIGR03122	1.2.99.5	4.10E-65	2056630	Candidatus_Korarchaeota_archaeon
	3300014914_102	276	COG2218	1.30E-75	arCOG00097	FwdC	6.60E-86	K00202	1.20E-79	270.6	102.5	PF01493	1.70E-11	TIGR03122	1.2.99.5	6.10E-76	2056630	Candidatus_Korarchaeota_archaeon
	3300024423_7	280	COG2218	2.30E-72	arCOG00097	FwdC	3.20E-77	K00202	1.30E-72	247.6	102.5	PF01493	7.90E-13	TIGR03122	1.2.99.5	1.10E-69	2056630	Candidatus_Korarchaeota_archaeon
	B10_G17	276	COG2218	1.30E-75	arCOG00097	FwdC	6.60E-86	K00202	1.20E-79	270.6	102.5	PF01493	1.70E-11	TIGR03122	1.2.99.5	6.10E-76	2056630	Candidatus_Korarchaeota_archaeon
	B14_G2	276	COG2218	1.30E-75	arCOG00097	FwdC	6.60E-86	K00202	1.20E-79	270.6	102.5	PF01493	1.70E-11	TIGR03122	1.2.99.5	6.10E-76	2056630	Candidatus_Korarchaeota_archaeon
	B15_G15	280	COG2218	4.40E-72	arCOG00097	FwdC	4.20E-77	K00202	2.00E-72	247	102.5	PF01493	2.00E-12	TIGR03122	1.2.99.5	5.00E-70	2056630	Candidatus_Korarchaeota_archaeon
	B41_G2	280	COG2218	1.70E-71	arCOG00097	FwdC	4.40E-76	K00202	1.80E-72	247.2	102.5	PF01493	2.10E-12	TIGR03122	1.2.99.5	3.40E-69	2056630	Candidatus_Korarchaeota_archaeon
	B51_G1	280	COG2218	1.40E-71	arCOG00097	FwdC	1.30E-76	K00202	1.60E-72	247.3	102.5	PF01493	2.80E-12	TIGR03122	1.2.99.5	3.70E-69	2056630	Candidatus_Korarchaeota_archaeon
	B8_G17	280	COG2218	7.20E-73	arCOG00097	FwdC	2.90E-79	K00202	6.30E-74	251.9	102.5	PF01493	5.90E-13	TIGR03122	1.2.99.5	2.80E-68	2056630	Candidatus_Korarchaeota_archaeon
	Flange_M5_B13	280	COG2218	7.40E-70	arCOG00097	FwdC	1.50E-73	K00202	1.70E-71	243.9	102.5	PF01493	6.70E-12	TIGR03122	1.2.99.5	2.90E-67	2056630	Candidatus_Korarchaeota_archaeon
	Flange_M5_B24	275	COG2218	4.40E-76	arCOG00097	FwdC	1.10E-76	K00202	1.20E-78	267.3	102.5	PF01493	3.40E-16	TIGR03122	1.2.99.5	1.10E-78	-	-
	Flange_M5_B4	279	COG2218	8.30E-90	arCOG00097	FwdC	5.90E-84	K00202	6.20E-88	297.8	102.5	PF01493	1.50E-17	TIGR03122	1.2.99.5	2.00E-84	-	-
	INS_M20_B145	276	COG2218	3.60E-85	arCOG00097	FwdC	6.10E-77	K00202	4.30E-83	281.9	102.5	PF01493	5.90E-10	TIGR03122	1.2.99.5	6.20E-79	2053489	Candidatus_Lokiarchaeota_archaeon
	INS_M20_B155	279	COG2218	1.00E-89	arCOG00097	FwdC	6.30E-84	K00202	6.50E-88	297.7	102.5	PF01493	1.50E-17	TIGR03122	1.2.99.5	2.10E-84	-	-
	INS_M20_B69	278	COG2218	9.90E-70	arCOG00097	FwdC	1.80E-67	K00202	3.10E-70	239.8	102.5	PF01493	5.00E-16	TIGR03122	1.2.99.5	1.80E-64	2056630	Candidatus_Korarchaeota_archaeon
	INS_M21_B31	275	COG2218	4.40E-76	arCOG00097	FwdC	1.10E-76	K00202	1.20E-78	267.3	102.5	PF01493	3.40E-16	TIGR03122	1.2.99.5	1.10E-78	-	-
	INS_M21_B37	276	COG2218	1.10E-84	arCOG00097	FwdC	4.50E-77	K00202	9.80E-83	280.8	102.5	PF01493	1.20E-10	TIGR03122	1.2.99.5	1.20E-78	2053489	Candidatus_Lokiarchaeota_archaeon
	INS_M34_B48	276	COG2218	1.10E-84	arCOG00097	FwdC	4.50E-77	K00202	9.80E-83	280.8	102.5	PF01493	1.20E-10	TIGR03122	1.2.99.5	1.20E-78	2053489	Candidatus_Lokiarchaeota_archaeon
	UWMA_0234	286	COG2218	1.10E-73	arCOG00097	FwdC	3.60E-78	K00202	1.30E-74	254.1	102.5	PF01493	1.20E-12	TIGR03122	1.2.99.5	9.20E-70	-	-
fwdD	3300014887_59	154	COG1153	5.70E-33	arCOG02674	FwdD	9.20E-28	K00203	1.80E-37	131.5	123.9	PF01568	5.40E-14	TIGR01591	1.2.1.2	9.90E-07	2056630	Candidatus_Korarchaeota_archaeon

wdE

	3300014887_59	126	COG1153	1.30E-21	arCOG02674	FwdD	1.60E-23	K00203	4.80E-27	97.7	123.9	PF01568	3.90E-16	-	-	-	2056630	Candidatus_Korarchaeota_archaeon
	3300014914_102	129	COG1153	3.10E-33	arCOG02674	FwdD	5.10E-28	K00203	6.10E-38	133	123.9	PF01568	3.20E-14	TIGR01591	1.2.1.2	9.10E-07	2056630	Candidatus_Korarchaeota_archaeon
	B10_G17	154	COG1153	5.70E-33	arCOG02674	FwdD	9.20E-28	K00203	1.80E-37	131.5	123.9	PF01568	5.40E-14	TIGR01591	1.2.1.2	9.90E-07	2056630	Candidatus_Korarchaeota_archaeon
	B10_G17	129	COG1153	1.40E-21	arCOG02674	FwdD	1.50E-23	K00203	4.90E-27	97.7	123.9	PF01568	4.20E-16	-	-	-	2056630	Candidatus_Korarchaeota_archaeon
	B14_G2	129	COG1153	2.20E-21	arCOG02674	FwdD	2.40E-23	K00203	9.20E-27	96.8	123.9	PF01568	5.00E-16	-	-	-	2056630	Candidatus_Korarchaeota_archaeon
	B14_G2	129	COG1153	3.10E-33	arCOG02674	FwdD	5.10E-28	K00203	6.10E-38	133	123.9	PF01568	3.20E-14	TIGR01591	1.2.1.2	9.10E-07	2056630	Candidatus_Korarchaeota_archaeon
	Flange_M5_B4	131	COG1153	1.70E-32	arCOG02674	FwdD	3.60E-27	K00203	1.20E-36	128.8	123.9	PF01568	1.60E-17	TIGR01591	1.2.1.2	1.40E-07	2026714	Candidatus_Bathyarchaeota_archaeon
	INS_M11_B49	128	COG1153	8.10E-34	arCOG02674	FwdD	1.10E-27	K00203	1.70E-38	134.8	123.9	PF01568	1.50E-16	TIGR01591	1.2.1.2	1.40E-07	-	-
	INS_M20_B145	128	COG1153	9.00E-34	arCOG02674	FwdD	3.60E-28	K00203	2.50E-38	134.2	123.9	PF01568	1.40E-16	TIGR01591	1.2.1.2	2.20E-07	-	-
	INS_M20_B155	131	COG1153	1.70E-32	arCOG02674	FwdD	3.60E-27	K00203	1.20E-36	128.8	123.9	PF01568	1.60E-17	TIGR01591	1.2.1.2	1.40E-07	2026714	Candidatus_Bathyarchaeota_archaeon
	INS_M21_B31	130	COG1153	3.10E-37	arCOG02674	FwdD	6.20E-34	K00203	2.90E-46	159.9	123.9	PF01568	5.30E-15	TIGR01591	1.2.1.2	1.90E-08	-	-
	INS_M21_B31	129	COG1153	1.40E-26	arCOG02674	FwdD	9.90E-25	K00203	1.60E-29	105.8	123.9	PF01568	1.30E-17	TIGR02693	1.20.9.1	4.30E-09	2056630	Candidatus_Korarchaeota_archaeon
	INS_M21_B37	128	COG1153	8.10E-34	arCOG02674	FwdD	1.10E-27	K00203	1.70E-38	134.8	123.9	PF01568	1.50E-16	TIGR01591	1.2.1.2	1.40E-07	-	-
	INS_M22_B49	128	COG1153	4.60E-34	arCOG02674	FwdD	4.80E-28	K00203	1.00E-38	135.5	123.9	PF01568	8.90E-17	TIGR01591	1.2.1.2	9.30E-08	-	-
	INS_M34_B48	128	COG1153	8.10E-34	arCOG02674	FwdD	1.10E-27	K00203	1.70E-38	134.8	123.9	PF01568	1.50E-16	TIGR01591	1.2.1.2	1.40E-07	-	-
	3300014913_21	131	COG2191	5.10E-24	arCOG00764	FwdE	2.50E-26	K11261	4.80E-21	78.5	45.1	PF02663	7.20E-23	-	-	-	-	-
	3300024423_7	132	COG2191	2.20E-21	arCOG00764	FwdE	7.80E-22	K11261	1.10E-17	67.6	45.1	PF02663	4.30E-19	-	-	-	2056630	Candidatus_Korarchaeota_archaeon
	B10_G17	151	COG2191	1.10E-16	arCOG00764	FwdE	2.50E-22	K11261	3.30E-12	49.6	45.1	PF02663	1.40E-15	-	-	-	2056630	Candidatus_Korarchaeota_archaeon
	B14_G2	151	COG2191	1.10E-16	arCOG00764	FwdE	2.20E-22	K11261	3.40E-12	49.6	45.1	PF02663	1.40E-15	-	-	-	2056630	Candidatus_Korarchaeota_archaeon
	B41_G2	132	COG2191	1.20E-20	arCOG00764	FwdE	1.20E-22	K11261	6.60E-17	65	45.1	PF02663	4.50E-19	-	-	-	2056630	Candidatus_Korarchaeota_archaeon
	B51_G1	93	COG2191	7.80E-08	arCOG00764	FwdE	3.50E-12	K11261	2.50E-06	30.4	45.1	PF02663	1.60E-08	-	-	-	2056630	Candidatus_Korarchaeota_archaeon
	B8_G17	132	COG2191	7.50E-23	arCOG00764	FwdE	8.10E-24	K11261	1.10E-17	67.6	45.1	PF02663	1.40E-20	-	-	-	2056630	Candidatus_Korarchaeota_archaeon
	Flange_M5_B13	132	COG2191	4.30E-21	arCOG00764	FwdE	1.20E-22	K11261	5.60E-18	68.5	45.1	PF02663	4.70E-19	-	-	-	2056630	Candidatus_Korarchaeota_archaeon
	Flange_M5_B24	141	COG2191	1.20E-19	arCOG00764	FwdE	5.00E-17	K11261	9.60E-17	64.4	45.1	PF02663	4.00E-17	-	-	-	-	-
	INS_M20_B145	141	COG2191	9.90E-20	arCOG00764	FwdE	3.70E-17	K11261	6.70E-17	65	45.1	PF02663	5.60E-17	-	-	-	-	-
	INS_M20_B69	130	COG2191	4.10E-23	arCOG00764	FwdE	1.50E-25	K11261	3.40E-21	79	45.1	PF02663	2.30E-22	-	-	-	2250277	Thermoprotei_archaeon
	INS_M21_B37	133	COG2191	1.90E-19	arCOG00764	FwdE	4.00E-17	K11261	1.10E-16	64.3	45.1	PF02663	4.70E-17	-	-	-	-	-
	INS_M34_B48	141	COG2191	1.00E-19	arCOG00764	FwdE	3.70E-17	K11261	6.70E-17	65	45.1	PF02663	5.60E-17	-	-	-	-	-
	UWMA_0234	132	COG2191	1.40E-21	arCOG00764	FwdE	6.70E-22	K11261	4.00E-16	62.4	45.1	PF02663	6.60E-19	-	-	-	-	-
fwdF	3300014887_59	139	COG2221	1.10E-16	arCOG02180	NapF	1.40E-15	K00205	1.30E-21	80.1	215.8	PF00037	3.50E-14	TIGR01971	-	3.10E-15	2056630	Candidatus_Korarchaeota_archaeon
fwdF	3300014887_59	453	COG1145	3.00E-48	arCOG02180	NapF	4.50E-81	K00205	8.70E-91	307.7	215.8	PF00037	1.40E-32	TIGR01971	-	3.90E-33	2056630	Candidatus_Korarchaeota_archaeon
fwdF	INS_M21_B31	454	COG1149	2.40E-49	arCOG02180	NapF	3.10E-83	K00205	1.60E-94	320	215.8	PF00037	7.60E-34	TIGR01971	-	1.50E-36	2026714	Candidatus_Bathyarchaeota_archaeon
fwdF	3300014914_102	139	COG2221	1.10E-16	arCOG02180	NapF	1.40E-15	K00205	1.30E-21	80.1	215.8	PF00037	3.50E-14	TIGR01971	-	3.10E-15	2056630	Candidatus_Korarchaeota_archaeon
fwdF	B14_G2	453	COG1145	3.00E-48	arCOG02180	NapF	4.50E-81	K00205	8.70E-91	307.7	215.8	PF00037	1.40E-32	TIGR01971	-	3.90E-33	2056630	Candidatus_Korarchaeota_archaeon
fwdF	B14_G2	139	COG2221	1.10E-16	arCOG02180	NapF	1.40E-15	K00205	1.30E-21	80.1	215.8	PF00037	3.50E-14	TIGR01971	-	3.10E-15	2056630	Candidatus_Korarchaeota_archaeon
fwdF	B10_G17	139	COG2221	1.10E-16	arCOG02180	NapF	1.40E-15	K00205	1.30E-21	80.1	215.8	PF00037	3.50E-14	TIGR01971	-	3.10E-15	2056630	Candidatus_Korarchaeota_archaeon
fwdF	B10_G17	453	COG1145	3.00E-48	arCOG02180	NapF	4.50E-81	K00205	8.70E-91	307.7	215.8	PF00037	1.40E-32	TIGR01971	-	3.90E-33	2056630	Candidatus_Korarchaeota_archaeon
fwdG	3300014887_59	66	-	-	arCOG00292	NapF	4.50E-10	K11260	2.60E-11	47	76.63	-	-	-	-	-	2056630	Candidatus_Korarchaeota_archaeon
fwdG	INS_M21_B31	151	COG4871	4.00E-19	arCOG00292	NapF	2.40E-28	K11260	2.70E-35	124.8	76.63	PF04060	4.30E-12	TIGR01944	-	1.10E-07	2056630	Candidatus_Korarchaeota_archaeon
fwdG	B14_G2	146	COG4871	6.70E-18	arCOG00292	NapF	2.30E-27	K11260	1.50E-37	132.1	76.63	PF04060	8.50E-12	TIGR01944	-	1.90E-07	2056630	Candidatus_Korarchaeota_archaeon
fwdG	B10_G17	66	-	-	arCOG00292	NapF	4.50E-10	K11260	2.60E-11	47	76.63	-	-	-	-	-	2056630	Candidatus_Korarchaeota_archaeon
fwdH	INS_M21_B31	68	COG1149	1.10E-10	arCOG04074	NapF	6.00E-10	K00204	1.60E-08	37.8	157	PF00037	1.20E-05	TIGR02951	-	1.90E-05	-	-
fwdH	Flange_M5_B13	56	COG1149	4.50E-09	arCOG02189	NapF	1.20E-06	K00204	7.60E-07	32.4	157	PF14697	6.90E-06	TIGR01944	-	0.00032	-	-
fwdH	B8_G17	153	COG2221	3.00E-17	arCOG02189	NapF	2.50E-19	K00204	4.70E-11	46	157	PF00037	7.20E-14	TIGR01944	-	1.60E-06	2056630	Candidatus_Korarchaeota_archaeon
fwdH	INS_M20_B155	66	COG1149	1.80E-16	arCOG04074	NapF	4.50E-12	K00204	1.80E-12	50.6	157	PF00037	7.40E-14	TIGR02179	-	7.50E-11	2056630	Candidatus_Korarchaeota_archaeon
fwdH	B14_G2	58	COG2768	3.70E-14	arCOG00958	NapF	4.10E-12	K00204	7.00E-09	39	157	PF00037	1.90E-11	TIGR02700	-	3.20E-08	2056630	Candidatus_Korarchaeota_archaeon
fwdH	INS_M34_B48	75	COG1145	3.70E-16	arCOG00289	NfnB	2.30E-12	K00204	2.00E-12	50.4	157	PF00037	4.00E-12	TIGR04395	1.97.-.-	2.90E-09	2026714	Candidatus_Bathyarchaeota_archaeon
fwdH	INS_M34_B48	87	COG1149	6.40E-16	arCOG00296	NapF	6.50E-12	K00204	3.60E-11	46.4	157	PF00037	7.80E-10	TIGR01944	-	2.00E-08	2803863	Clostridium_sp._YIM_B02565
fwdH	INS_M20_B69	492	COG2221	8.80E-30	arCOG02187	NapF	1.20E-41	K00204	3.40E-20	75.7	157	PF00037	5.10E-16	TIGR04041	1.97.1.-	8.60E-15	-	-

INS_M22_B49	75	COG1145	4.20E-16	arCOG00289	NfnB	1.60E-12	K00204	2.00E-12	50.5	157	PF00037	4.00E-12	TIGR04395	1.97.-.-	4.10E-09	2026714	Candidatus_Bathyarchaeota_archaeon
B41_G2	322	COG2221	5.20E-17	arCOG02189	NapF	1.80E-30	K00204	7.10E-11	45.4	157	PF00037	5.00E-10	TIGR01944	-	1.50E-08	2056630	Candidatus_Korarchaeota_archaeon
INS_M20_B145	75	COG1145	4.40E-16	arCOG00289	NfnB	3.60E-12	K00204	2.00E-12	50.5	157	PF00037	4.00E-12	TIGR04395	1.97.-.-	2.30E-09	2026714	Candidatus_Bathyarchaeota_archaeon
INS_M20_B145	87	COG1149	1.40E-15	arCOG00296	NapF	2.10E-11	K00204	1.20E-10	44.8	157	PF00037	2.00E-09	TIGR01944	-	5.10E-08	349931	Clostridium_cavendishii
Flange_M5_B4	66	COG1149	1.80E-16	arCOG04074	NapF	4.50E-12	K00204	1.80E-12	50.6	157	PF00037	7.40E-14	TIGR02179	-	7.50E-11	2056630	Candidatus_Korarchaeota_archaeon
B51_G1	66	COG1145	1.70E-13	arCOG00289	NfnB	5.10E-10	K00204	6.90E-11	45.5	157	PF12837	1.00E-11	TIGR02179	-	2.20E-06	2056630	Candidatus_Korarchaeota_archaeon
B51_G1	322	COG2221	2.90E-16	arCOG02189	NapF	3.10E-30	K00204	4.30E-10	42.9	157	PF00037	3.30E-09	TIGR01944	-	1.10E-07	2056630	Candidatus_Korarchaeota_archaeon
B51_G1	71	COG2768	5.70E-17	arCOG02460	NapF	1.30E-10	K00204	4.50E-13	52.6	157	PF00037	7.30E-14	TIGR04105	1.12.-.-	2.20E-08	2056630	Candidatus_Korarchaeota_archaeon
B15_G15	71	COG2768	2.60E-16	arCOG02460	NapF	7.20E-10	K00204	3.70E-12	49.6	157	PF00037	1.40E-12	TIGR02912	1.8.-.-	1.70E-07	2056630	Candidatus_Korarchaeota_archaeon
B15_G15	66	COG1145	1.70E-13	arCOG00289	NfnB	5.10E-10	K00204	6.90E-11	45.5	157	PF12837	1.00E-11	TIGR02179	-	2.20E-06	2056630	Candidatus_Korarchaeota_archaeon
3300024423_7	67	COG1145	3.00E-14	arCOG00289	NfnB	3.60E-10	K00204	3.60E-11	46.4	157	PF00037	1.60E-11	TIGR02179	-	1.70E-06	2056630	Candidatus_Korarchaeota_archaeon
GS19_ROV16_BS03_Bin_00077	66	COG1149	2.00E-16	arCOG00289	NfnB	2.70E-12	K00204	2.30E-12	50.3	157	PF00037	2.50E-13	TIGR02179	-	1.20E-10	2026714	Candidatus_Bathyarchaeota_archaeon
INS_M21_B37	75	COG1145	4.20E-16	arCOG00289	NfnB	1.60E-12	K00204	2.00E-12	50.5	157	PF00037	4.00E-12	TIGR04395	1.97.-.-	4.10E-09	2026714	Candidatus_Bathyarchaeota_archaeon
INS_M21_B37	87	COG2768	9.00E-16	arCOG00296	NapF	1.10E-11	K00204	6.20E-11	45.6	157	PF12838	2.30E-09	TIGR01944	-	2.50E-08	349931	Clostridium_cavendishii
Flange_M5_B24	75	COG1145	3.70E-16	arCOG00289	NfnB	2.30E-12	K00204	2.00E-12	50.4	157	PF00037	4.00E-12	TIGR04395	1.97.-.-	2.90E-09	2026714	Candidatus_Bathyarchaeota_archaeon
Flange_M5_B24	87	COG1149	6.40E-16	arCOG00296	NapF	6.50E-12	K00204	3.60E-11	46.4	157	PF00037	7.80E-10	TIGR01944	-	2.00E-08	2803863	Clostridium_sp._YIM_B02565
